# Clients’ perceptions and experiences of targeted digital communication accessible via mobile devices for reproductive, maternal, newborn, child, and adolescent health: a qualitative evidence synthesis

**DOI:** 10.1002/14651858.CD013447

**Published:** 2019-10-14

**Authors:** Heather MR Ames, Claire Glenton, Simon Lewin, Tigest Tamrat, Eliud Akama, Natalie Leon

**Affiliations:** Norwegian Institute of Public HealthPostboks 222 SkøyenOsloNorway0213; South African Medical Research CouncilHealth Systems Research UnitPO Box 19070Cape TownSouth Africa7505; World Health OrganizationDepartment of Reproductive Health and Research20 Avenue AppiaGenevaSwitzerlandCH‐1211; University of WashingtonSeattleWashingtonUSA

## Abstract

**Background:**

Governments and health systems are increasingly using mobile devices to communicate with patients and the public. Targeted digital client communication is when the health system transmits information to particular individuals or groups of people, based on their health or demographic status. Common types of targeted client communication are text messages that remind people to go to appointments or take their medicines. Other types include phone calls, interactive voice response, or multimedia messages that offer healthcare information, advice, monitoring, and support.

**Objectives:**

To explore clients' perceptions and experiences of targeted digital communication via mobile devices on topics related to reproductive, maternal, newborn, child, or adolescent health (RMNCAH).

**Search methods:**

We searched MEDLINE (OvidSP), MEDLINE In‐Process & Other Non‐Indexed Citations (OvidSP), Embase (Ovid), World Health Organization Global Health Library, and POPLINE databases for eligible studies from inception to 3‐6 July 2017 dependant on the database (See appendix 2).

**Selection criteria:**

We included studies that used qualitative methods for data collection and analysis; that explored clients' perceptions and experiences of targeted digital communication via mobile device in the areas of RMNCAH; and were from any setting globally.

**Data collection and analysis:**

We used maximum variation purposive sampling for data synthesis, employing a three‐step sampling frame. We conducted a framework thematic analysis using the Supporting the Use of Research Evidence (SURE) framework as our starting point. We assessed our confidence in the findings using the GRADE‐CERQual (Confidence in the Evidence from Reviews of Qualitative research) approach. We used a matrix approach to explore whether potential implementation barriers identified in our synthesis had been addressed in the trials included in the related Cochrane Reviews of effectiveness.

**Main results:**

We included 35 studies, from a wide range of countries on six continents. Nineteen studies were conducted in low‐ and middle‐income settings and sixteen in high‐income settings. Some of the studies explored the views of people who had experienced the interventions, whereas others were hypothetical in nature, asking what people felt they would like from a digital health intervention. The studies covered a range of digital targeted client communication, for example medication or appointment reminders, prenatal health information, support for smoking cessation while pregnant, or general sexual health information.

Our synthesis showed that clients' experiences of these types of programmes were mixed. Some felt that these programmes provided them with feelings of support and connectedness, as they felt that someone was taking the time to send them messages (moderate confidence in the evidence). They also described sharing the messages with their friends and family (moderate confidence).

However, clients also pointed to problems when using these programmes. Some clients had poor access to cell networks and to the internet (high confidence). Others had no phone, had lost or broken their phone, could not afford airtime, or had changed their phone number (moderate confidence). Some clients, particularly women and teenagers, had their access to phones controlled by others (moderate confidence). The cost of messages could also be a problem, and many thought that messages should be free of charge (high confidence). Language issues as well as skills in reading, writing, and using mobile phones could also be a problem (moderate confidence).

Clients dealing with stigmatised or personal health conditions such as HIV, family planning, or abortion care were also concerned about privacy and confidentiality (high confidence). Some clients suggested strategies to deal with these issues, such as using neutral language and tailoring the content, timing, and frequency of messages (high confidence).

Clients wanted messages at a time and frequency that was convenient for them (moderate confidence). They had preferences for different delivery channels (e.g. short message service (SMS) or interactive voice response) (moderate confidence). They also had preferences about message content, including new knowledge, reminders, solutions, and suggestions about health issues (moderate confidence). Clients' views about who sent the digital health communication could influence their views of the programme (moderate confidence).

For an overview of the findings and our confidence in the evidence, please see the 'Summary of qualitative findings' tables.

Our matrix shows that many of the trials assessing these types of programmes did not try to address the problems we identified, although this may have been a reporting issue.

**Authors' conclusions:**

Our synthesis identified several factors that can influence the successful implementation of targeted client communication programmes using mobile devices. These include barriers to use that have equity implications. Programme planners should take these factors into account when designing and implementing programmes. Future trial authors also need to actively address these factors and to report their efforts in their trial publications.

## Plain language summary

**What are clients' experiences and perceptions of receiving health information via their mobile phones?**

**What is the aim of this synthesis?**

The aim of this Cochrane qualitative evidence synthesis was to explore clients' views and experiences of being communicated with by the health system through their mobile phone. Our synthesis looked at communication about pregnancy, newborn, and child health, sexual health, and family planning. By synthesis we mean the bringing together and synthesising of results from primary qualitative studies into a larger whole. We collected all relevant studies and included 35 studies in the synthesis.

This synthesis links to other Cochrane Reviews that assess the effectiveness of this type of targeted digital health communication.

**Key messages**

Many clients like receiving messages from the health services by mobile phone. However, some clients have problems receiving messages due to lack of network access, internet, or phone, or language, reading, or privacy issues. Clients' experiences are also influenced by message timing, frequency, content, and sender.

**What did we study in the synthesis?**

Governments and health systems are starting to use mobile phones to communicate with clients. When the information is targeted at particular people or groups of people, and when the health system decides when and what to communicate, this is called 'digital targeted client communication.' Common types of digital targeted client communication are text messages that remind people to go to appointments or to take their medicines. Other types include phone calls or video messages that offer healthcare information, advice, monitoring, and support.

We looked for studies of clients' views and experiences of targeted communication by mobile phone. We focused on communication with pregnant women and parents of young children, and with adults and teenagers about sexual health and family planning.

**What are the main findings of the synthesis?**

We included 35 studies from around the world. These studies showed that clients' experiences of these types of programmes were mixed. Some felt that these programmes provided them with feelings of support and connectedness, as they felt that someone was taking the time to send them messages (moderate confidence in the evidence). Others also described sharing the messages with their friends and family (moderate confidence).

However, clients also pointed to problems when using these programmes. Some clients had poor access to cell networks and to the internet (high confidence). Others had no phone, had lost or broken their phone, could not afford airtime, or had changed their phone number (moderate confidence). Some clients, particularly women and teenagers, had their access to phones controlled by others (moderate confidence). The cost of messages could also be a problem, and many thought that messages should be free of charge (high confidence). Languages issues as well as clients' skills in reading, writing, and using mobile phones could also be a problem (moderate confidence).

Clients dealing with stigmatised or personal health conditions such as HIV, family planning, or abortion care were concerned about privacy and confidentiality (high confidence). Some suggested strategies to deal with these issues, such as using neutral language and tailoring the content, timing, and frequency of messages (high confidence).

Clients wanted messages at a time and frequency that was convenient for them (moderate confidence). They had preferences for different delivery channels (e.g. short message service (SMS) or interactive voice response) (moderate confidence). They also had preferences about message content, including new knowledge, reminders, solutions, and suggestions about health issues (moderate confidence). Clients' views about who sent the digital health communication could influence their views of the programme, and many people wanted a sender that they knew and trusted (moderate confidence).

**How up‐to‐date is the synthesis?**

We searched for studies published before July 2017.

## Background

'Digital health' is an overarching term for the use of information and communication technology (ICT) for health purposes. The term has more recently been used as “a broad umbrella term encompassing eHealth, mHealth, as well as emerging areas such as the use of advanced computing sciences, 'big data,' genomics and artificial intelligence” (WHO 2018).

The use of digital technology for health has emerged as an important innovation with the potential to strengthen health systems in many settings. This potential to address health system challenges and to improve the delivery of services has propelled significant investments into digital health, particularly in low‐ and middle‐income countries (LMICs). Governments have access to a broad range of digital health tools, but there are gaps in the evidence on the effectiveness, feasibility, and acceptability of digital health interventions (Aranda‐Jan 2014; Gurol‐Urganci 2013; Vervloet 2012).

Digital health interventions have shown potential for improving the efficiency and effectiveness of health service delivery and health system functioning, the latter referring to digital tools for strengthening key health systems functions such as leadership and governance, finance, human resource, and health information systems, as well as equipment and medicine supply systems. This includes a wide range of applications for electronic monitoring and evaluation, clinical support decision‐making tools, electronic diagnostics and prescribing systems, increased access to health services in remote areas, co‐ordination and knowledge exchange between different cadres and levels of health workers, electronic management and administration systems, and for improving health service responsiveness and patient‐orientated change interventions aimed at improved patient self‐care and health awareness (Catalani 2013, Naghizadeh 2017).

Among the most common areas for digital health interventions (perhaps due to the high prevalence of mobile phone use globally, including in low‐resource settings) is patient‐orientated change aimed at improved self‐care and self‐management of health and illness, through for instance digital health reminders of appointment and general health promotion messaging (Gurol‐Urganci 2013). Although there is increasing evidence that digital targeted client communication may improve patient adherence behaviour, less is known about the acceptability, relevance, and usefulness of these interventions from the perspective of the client population. This information is needed to inform practice and policy on optimising the design, implementation, and improvement of digital targeted client communication interventions.

### Description of the topic of interest

Within the field of digital health, there are a variety of ways digital technologies may be used for public health purposes. This review focused on digital targeted client communication (DTCC). Digital targeted client communication may be used to transmit health event alerts to specific population groups; deliver health information based on a known health status or demographic; alert and remind about a particular health behaviour; or transmit diagnostic results to clients (WHO 2018). Targeted communication can also be further customised according to an individual’s specific needs, resulting in 'tailored client communication,' whereby message content, timing, and frequency are matched to the needs and preferences of an individual (Hawkins 2008). The communication can be unidirectional and bidirectional, but initial contact is from the health system, as opposed to on‐demand information service and telemedicine, where the client initiates the first contact with the health system (WHO 2018). The purpose of the DTCC would be to improve health and well‐being, healthcare services, and/or the functioning of the health system. Typical interventions include sending brief text messages as a reminder to adhere to health visits and medical treatment, to provide clients with health information, to monitor their progress, and/or to provide medical advice and support.

### Why it is important to do this review

Through the World Health Assembly Resolution on Digital Health, Ministries of Health recognised that digital technologies can potentially bring value to the health system, but called for a better understanding of best practices and the promotion of evidence‐based digital health interventions and standards (WHO 2018a). This resolution also highlighted the need to ensure that “digital health solutions complement and enhance existing health service delivery models, strengthen integrated, people‐centred health services and contribute to improved population health, and health equity, including gender equality” and noted the lack of evidence on the impact of digital health in these respects (WHO May 2018).

To address this need, the World Health Organization (WHO) embarked on developing evidence‐based guidelines to inform government‐led investments in digital health interventions for health system strengthening, including mechanisms to bolster access to reproductive, maternal, newborn, child, or adolescent health (RMNCAH) services. This qualitative evidence synthesis is among a series of systematic reviews informing the WHO guidelines on digital interventions for health system strengthening. The scope of this synthesis reflects the WHO’s assessment of global intervention priorities in this area. In addition to contributing to the WHO guideline, the findings of this review will be of interest more generally to programme planners and policymakers when deciding if and how to implement DTCC via mobile device in their setting. This review will complement the two WHO‐commissioned reviews that focus on the effectiveness of targeted digital communication via mobile device (Palmer Ongoing a, Palmer Ongoing).

Researchers in this field have also suggested that to better understand barriers and facilitators of successful implementation of digital interventions, clients’ and healthcare providers’ perceptions of the safety of the interventions, potential harms, and adverse effects should be assessed and explored (Gurol‐Urganci 2013). Barriers may include privacy concerns (Ahmed 2017), poor access to reliable network coverage, and poor integration into existing health systems (Aranda‐Jan 2014). Perceptions that the technology empowers the user and improves communication may serve as facilitators to successful implementation of digital health interventions (Ahmed 2017). Reviewing and synthesising the qualitative evidence on perceptions and experiences of clients will not only complement the evidence emerging from the effectiveness reviews, but may also enhance our understanding of broader contextual, organisational, technical, social, and individual factors that may be shaping the development, implementation, and responses to targeted digital communication.

## Objectives

To explore clients' perceptions and experiences of targeted digital communication via mobile devices on topics related to reproductive, maternal, newborn, child, or adolescent health (RMNCAH).

## Methods

Criteria for considering studies for this review

### Topic of interest

We focused on clients’ perceptions and experiences of digital targeted client communication (DTCC) via mobile devices in the areas of reproductive, maternal, newborn, child, or adolescent health (RMNCAH).

### Types of studies

We included primary studies that used qualitative study designs such as ethnography, phenomenology, case studies, and grounded theory as well as qualitative process evaluations. We included primary studies that used qualitative methods for data collection (e.g. individual interviews, focus group discussions, diaries, document analysis, open‐ended survey questions, and observation) and that used qualitative methods for data analysis (e.g. thematic analysis, framework analysis or grounded theory). We excluded primary studies that collected data using qualitative methods but did not perform a qualitative analysis (e.g. open‐ended survey questions where the responses are analysed using descriptive statistics only). We included mixed‐methods studies when it was possible to extract data that were collected and analysed using qualitative methods. We included studies regardless of whether they had been carried out alongside studies of the effectiveness of digital health interventions.

### Types of interventions

We included studies exploring clients’ experiences and perceptions of targeted digital communication (e.g. text messages and interactive voice response) accessible via mobile devices (see Table 1). This could include perceptions and experiences of the content of the message, the delivery mechanism itself, the sender, or other aspects tied to this form of communication.

We defined 'digital targeted client communication' (DTCC) as the transmission of targeted health content to a specified population, or to individuals within a predefined health or demographic group. This transmitted information can fall along a continuum of tailored (personalised to an individual person’s condition) to standard, general, untailored communication. It can include the transmission of individualised notifications according to a specific individual’s clinical care plan as well as the transmission of predetermined content developed for the identified population group (Hawkins 2008). Eligible individuals need to be identified and subscribed into a system that allows the transmission of the health information via digital device to a number they have requested. Additionally, the timing and content of the transmitted information should be determined by the health system, and not by a client seeking information on‐demand.

Examples of targeted client communication could include:

- providing targeted health education, promotion, or information to clients based on known health or demographic characteristics;
- providing alerts, notifications, and reminders to a client based on a clinical care plan or protocol, such as in the case of medication adherence and appointments to see a healthcare provider.

In contrast, untargeted client communication is the transmission of health promotion content to the general population or an undefined target population.

By mobile devices, we mean mobile phones or handheld mobile devices of any kind (but not analogue landline telephones), as well as tablets, personal digital assistants, and smartphones that facilitate communication to a targeted group of clients via different channels including short message service (SMS), voice, interactive voice response, multimedia messages, and social media when used for instant messaging purposes. For a specific list of included and excluded types of delivery mechanisms see Table 1 below.

#### Table 1: List of included and excluded mobile devices and platforms

**Table:** 

**Included**	**Excluded**
Mobile text messaging (including SMS and USSD) Interactive voice response (IVR) Voice calls and callbacks WhatsApp and other instant messaging services (such as Facebook Messenger) Multimedia messages, including video and audiovisual messages Applications that provide notifications to the client Communication in which the content and timing are predefined Web‐based intervention, if content development is optimised for mobile delivery or training and implementation support is based on the use of mobile devices Applications (apps) that provide targeted client communication, such as notifications to the client	Web portals, applications, and websites that do not have a targeted communication component to notify clients Emails alone that did not explicitly state transmission to mobile devices Social media websites such as Facebook, Baidu, and Twitter, unless there is explicit mention of the use of targeted communication or messaging services to individuals

We included targeted client communication that aimed to remind or recall; inform and educate; or provide support (Hill 2011; Kaufman 2017; Willis 2013).We included targeted client communication that focused on the health issues identified in Appendix 1. We derived this list of health issues from two key resources by the World Health Organization on Essential Interventions for Reproductive, Maternal, Newborn, Child, and Adolescent Health (RMNCAH) and Family Planning, Safe Abortion Care, Maternal, Newborn, and Child Health (PMNCH) (Partnership for Maternal Newborn Child Health 2011).We included studies where the message was initiated by a governmental or non‐governmental, private, or public organisation and was targeted at individuals or groups.

We included communication that was one‐way (e.g. triggered by a system to the defined population groups) or two‐way (e.g. allows for discussion or question and answer between the targeted population and the health system). Two‐way or bidrectional communication was included if the first communication was initiated by the health system or healthcare provider to a client's mobile device. Studies of bidirectional communication initiated by clients to contact the health system were included in another review related to telemedicine and client‐to‐provider consultations (Gonçalves‐Bradley 2018, WHO 2019).

We included studies where the digital component of the intervention was delivered as part of a wider package, or if we judged it to be the major component of the intervention. The focus of the study needed to be on one of the intervention areas listed in Table 1.

### Types of participants

The review focused on the following population groups as defined in relation to the WHO guideline for which this review was commissioned (WHO 2019).

We included studies that focused on the perceptions and experiences of clients. We define clients as “an individual who is a potential or current user of health services; may also be referred to as patient or non‐patient who uses health information and services” (WHO 2019).

We included studies that focused on the perceptions and experiences of clients in one or more of the following groups.

- Adolescent and youth populations (ages 10 to 24 years) that were users/potential users of sexual and reproductive health (SRH) services. Studies that included other population groups were included if participants’ age had been disaggregated or where it was explicitly mentioned that a minimum of 70% of participants were between the ages of 10 and 24 years.
- Adult users/potential users of SRH (age 18+). Studies that explicitly stated that they also included population groups under 18 years of age were included where it was explicitly mentioned that a minimum of 70% of the participants were above the age of 18 years.
- Pregnant and postpartum women up to six weeks' postpartum and their partners or others who support them.
- Pregnant and postpartum women living with HIV up to six weeks' postpartum and their partners or others who support them*,* with the exception of breastfeeding, for which it was six months' postpartum.
- Parents and caregivers of children under five years of age.

### Search methods for identification of studies

#### Electronic searches

Information Specialist John Eyers developed the search strategies in consultation with the review authors. We searched the following electronic databases for eligible studies between 3 and 6 July 2017, dependant on the data base (see Appendix 2).

- MEDLINE (OvidSP)
- MEDLINE In‐Process & Other Non‐Indexed Citations (OvidSP)
- Embase (Ovid)
- World Health Organization Global Health Library
- POPLINE

Using guidelines developed by the Cochrane Qualitative Research Methods Group for searching for qualitative evidence (Booth 2011, Harris 2018, Noyes 2015), as well as modified versions of the search for the associated or 'sister' effectiveness reviews (Palmer Ongoing a, Palmer Ongoing), we developed search strategies for each database. There were no language or geographic restrictions on the search. We used 1993 as the cut‐off date for the search, as the first commercial SMS message was sent in December 1992. A similar approach was taken in the related effectiveness reviews (Palmer Ongoing a, Palmer Ongoing).

#### Searching other resources

We asked the Guideline Development Group network for the WHO guideline on Digital Health Guidelines for Health System Strengthening to identify and send in any studies that fit the inclusion criteria on 28 June 2017 (WHO 2019).

We sent a public call for papers to global listservs, including Global Digital Health Network and Implementing Best Practices (IBP).We handsearched the database www.mHealthEvidence.org for any studies that met our inclusion criteria on 17 August 2017. This database is designed to bring together literature on digital health from a global perspective to help stakeholders quickly access up‐to‐date, relevant evidence.

We searched PubMed for all studies linked to the trials included in the related effectiveness reviews in September and October 2018.

### Data collection, management, and synthesis

#### Selection of studies

We collated records identified from different sources into Covidence, a systematic review screening tool (Covidence). We identified duplicates and removed them. Three review authors then independently assessed the titles and abstracts of the identified records to determine potential eligibility, discarding those that were clearly irrelevant to the topic. Review authors HA and EA screened all titles and abstracts, and TT resolved any conflicts.

We obtained the the full text of all the papers identified as potentially relevant, and two review authors (TT, EA or HA) independently assessed these for inclusion in the review. NL resolved disagreements. See Characteristics of excluded studies for a list of the excluded studies and the main reasons for exclusion.

#### Translation of studies in languages other than English

Although we searched for languages spoken by at least one member of the review team (French, English, Scandinavian languages), all of the identified or included studies were in the English language.

#### Sampling of studies

As qualitative evidence synthesis aims for variation in concepts rather than an exhaustive sample, and because large numbers of studies can impair the quality of the analysis, we purposefully sampled from the 52 articles that met our inclusion criteria.

We developed a sampling frame that took into consideration the population group, data richness, and closeness of the study data to the review objective.

Firstly, we divided the studies that met our inclusion criteria by client group as listed above in the inclusion criteria. As there were a limited number of included studies for pregnant and postpartum women (up to six weeks) (seven studies) and for pregnant and postpartum women (up to six weeks) living with HIV (two studies), all studies were included.

Secondly, we assessed the included studies within each client group for data richness, using a scale of 1 to 5 (see Appendix 3), and also looked at how closely the data from the study matched the review objectives. Studies with 'thin data' or that did not provide a close match to our review objective were not sampled.

In total, we sampled 35 studies to be included in the analysis (see Table 2 below).

##### Table 2: Sampled studies included in the synthesis per client group

**Table:** 

Adolescent and youth populations as potential users of SRH services	Adult populations as potential users of SRH services	Pregnant and postpartum women (up to 6 weeks)	Pregnant and postpartum women (up to 6 weeks) living with HIV	Parents and other caregivers of children under 5 years of age
12	10	7	2	4

#### Data extraction

We performed data extraction using a data extraction form designed specifically for this synthesis. We used the form to extract key themes and categories relevant to the synthesis objective using the Supporting the Use of Research Evidence (SURE) framework (SURE Collaboration 2011). We used a second form to extract information about first author, date of publication, language, country of study, context (urban, rural), and participant group to which the intervention was directed. We also extracted information on research method and if theoretical or conceptual frameworks were used.

HA extracted data from all the sampled studies. EA double‐checked the data extraction and verified that all relevant data were extracted.

#### Assessment of the methodological limitations of included studies

To assess the methodological quality of the included studies, we applied a quality appraisal framework to each study. We used an adaptation of the Critical Appraisal Skills Programme (CASP) quality assessment tool for qualitative studies (CASP 2018). Other reviews of qualitative evidence have also used this tool (Ames 2017; Glenton 2013; Gopinathan 2014; Lewin 2010). The adapted tool that we used included the following eight questions.

1. Are the setting/s and context described adequately?
2. Is the sampling strategy described, and is this appropriate?
3. Is the data collection strategy described and justified?
4. Is the data analysis described, and is this appropriate?
5. Are the claims made/findings supported by sufficient evidence?
6. Is there evidence of reflexivity?
7. Does the study demonstrate sensitivity to ethical concerns?
8. Any other concerns?

HA conducted the initial assessment, and NL and TT reviewed the assessments. We accept that there is no ‘gold standard’ approach for assessing the methodological quality of primary qualitative studies, but believe that this adapted CASP checklist fit our needs in the context of this synthesis.

We did not exclude any studies based on our assessment of methodological limitations, but used this information to assess our confidence in the synthesis findings, as part of the GRADE‐CERQual (Confidence in the Evidence from Reviews of Qualitative research) approach (Lewin 2018).

#### Data management and synthesis

For our synthesis, we first grouped articles according to client group as defined above. However, since there were only two articles focusing on pregnant and postpartum women living with HIV, we combined these with the other studies focusing on pregnant and postpartum women.

We conducted an initial framework analysis using the SURE framework to identify themes in the data. We did this within each of the population groups and then looked across population groups. The SURE framework has been used as an analysis framework in other studies and reviews (Glenton 2013; Glenton 2016; Gopinathan 2014; Lewin 2010; Muloliwa Forthcoming; Oku 2017). We used the headings and subheadings from the SURE framework as a starting point for the analysis and then adapted them through an iterative process. Next, within each section of the framework we did a thematic analysis of the extracted data to identify our synthesis findings. For example, data included in the framework under 'health systems constraints ‐ accessibility of care' were thematically synthesised, and findings around access to digital devices and interventions were identified (see Findings 5 to 9 in the Results). Another example is around the framework area of knowledge and skills. Here we adapted the category to look at knowledge and skills in relationship to using a mobile device. A final example of adaptation is under health systems constraints, relationships with norms and standards. We adapted this category to address issues related to privacy and confidentiality. Some areas of the framework were left empty and discarded. Once findings were identified, HA read through all of the sampled studies again to double‐check data extraction. We also went through the findings and identified those where the contributing studies were only/predominantly from high‐income (HIC) or LMIC settings. The same was done for the different client groups. We have indicated this in the detailed description of the relevant findings.

We then thematically analysed the 25 identified findings in order to group them into six related overarching categories to provide a narrative for the Findings section. Some categories reflect those within the SURE framework, whereas others have been reorganised to address different issues raised by clients. The six overarching categories related to the general acceptability of and preferences around DTCC; the varying degrees of access to network services, phones, and messages; communication delivery and format preferences; communication content preferences; privacy and confidentiality regarding personal health information; and the perceptions of intervention impact.

To create the summary of findings for the Abstract we took all of the findings with moderate or high confidence in the evidence and worked them together into a clear story line.

#### Appraisal of confidence in the review findings

Four review authors (HA, CG, SL, NL) used GRADE‐CERQual to assess the confidence that can be placed in each review finding (Lewin 2018). Each finding was assessed by at least two review authors.

The GRADE‐CERQual approach assesses confidence in the evidence based on the following four components (Lewin 2018a).

1. Methodological limitations of included studies: the extent to which there are concerns about the design or conduct of the primary studies that contributed evidence to an individual review finding.
2. Coherence of the review finding: an assessment of how clear and cogent the fit is between the data from the primary studies and a review finding that synthesises that data.
3. Adequacy of the data contributing to a review finding: an overall determination of the degree of richness and quantity of data supporting a review finding.
4. Relevance of the included studies to the review question: the extent to which the body of evidence from the primary studies supporting a review finding is applicable to the phenomenon of interest (perspective or population, context, setting) specified in the review question.

After assessing each of the four components, we made a judgement about the overall confidence in the evidence supporting each review finding. We judged confidence as high, moderate, low, or very low. The final assessment was based on consensus among the review authors. All findings started as high confidence and were then graded down if there were important concerns regarding any of the CERQual components (Lewin 2018).

#### 'Summary of qualitative findings' tables

We concluded the appraisal of confidence in each review finding by drafting a 'Summary of qualitative findings' table that presents the findings and our assessment of confidence in these findings, as well as an explanation of this assessment, based on the GRADE‐CERQual approach.

#### Supplementing the related Cochrane effectiveness reviews with synthesised qualitative findings

We explored how the findings from our synthesis related to, and could help to inform, the findings of the two related Cochrane reviews of effectiveness of DTCC (Palmer Ongoing a; Palmer Ongoing). To do this we utilised a matrix approach similar to the one used previously by Candy 2011, Ames 2017, and Munabi‐Babigumira 2017. This approach has also been described by Harden 2018. Our matrix explored whether potential implementation barriers that we identified in our synthesis had been addressed in the programmes evaluated in the related reviews of effectiveness.

To create the matrix we undertook the following steps: firstly, we selected the synthesis findings that we had assessed as having high or moderate confidence and that presented potential barriers to the implementation of targeted client communication programmes. Secondly, we created 10 questions reflecting these potential barriers, and placed these in a table. Finally, we assessed whether any attempt had been made to address these implementation barriers in the trials that were included in the two related Cochrane Reviews of effectiveness.

To carry out this assessment, we examined the publications included in the two Cochrane Reviews of effectiveness (Palmer Ongoing a; Palmer Ongoing). We also performed a further search for additional publications that could be related to the trials. We did this by (1) examining the reference lists of the main trial publication; and (2) searching for each trial in PubMed, and doing an advanced search for 'Similar articles'. The advanced search for ‘Similar articles’ used the first author of the trial to identify possible related studies that had this author as a co‐author, and selected any that appeared to be related to the trial.

#### Researchers’ reflexivity

Within qualitative research, researchers are expected to reflect on their own background and position, and how it will affect the design, analysis, and reporting of their research. Throughout the data synthesis, the authors were aware of their own positions and reflected on how these could influence the data synthesis and study design. Several of the authors have both primary and evidence synthesis research experience in digital health (reporting positive, negative, and neutral findings), and they considered themselves to be agnostic as to the outcome of this evidence synthesis.

## Results

### Included studies

We screened 9531 abstracts and assessed 142 full‐text articles. Fifty‐two studies met our inclusion criteria. From these 52 studies, we sampled 35 studies for analysis (Figure 1). The 17 studies that met the inclusion criteria but were not sampled into the synthesis can be found in Table 1.

**1:**
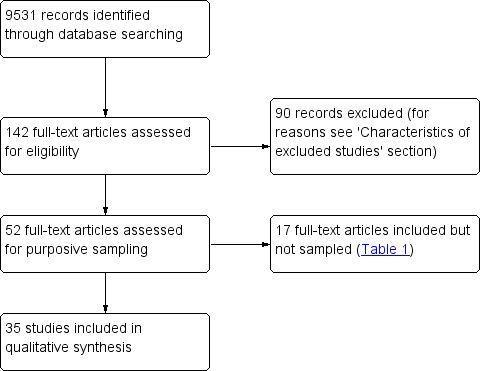
PRISMA flow diagram.

**1:** Studies that were included but not sampled

**Study ID**	**Reason not sampled**
Adetunji 2017	Thin data
Ahlers‐Schmidt 2012	Thin data
Ahlers‐Schmidt 2013	Thin data
Ahlers‐Schmidt 2014	Thin data
Anand 2017	Thin data
Atukunda 2017	Thin data
Baranoski 2014	Thin data
Cornelius 2012	Formative study not as close to the research objective as other included studies
Datta 2014	Thin data
George 2012	Thin data in comparison to other studies from the same setting
Graham 2015	Thin data
Harris 2013	Thin data
Martin 2016	Thin data
Montoya 2014	Thin data
Montoya 2015	Thin data
Smillie 2014 (Kenya)	Thin data
Swendeman 2015	Thin data

All of the sampled studies were published between 2009 and 2017. All of the included studies were published in English. Sixteen of the sampled studies were from high‐income countries: Australia (1), Canada (2), the UK (4), and the USA (9). Nineteen of the sampled studies were from low‐ or middle‐income countries: Cambodia (1), Cameroon (2), Ghana (1), India (2), Kenya (2), Lesotho (1), Nigeria (2), Peru (3), Sierra Leone (1), South Africa (2), and Uganda (2).

Client populations were adolescents and youth (12 studies); adult users/potential users of reproductive health services (10); pregnant and postpartum women (including those living with HIV) (9); and parents and caregivers of children under five years of age (4).

The included studies explored seven different methods or combinations of methods for delivering DTCC: app (2), interactive voice response (IVR) (1), IVR + SMS (1), SMS + voice call (1), SMS (27), mobile phone messaging (2), and mobile phones in general (1).

### Methodological limitations of the included qualitative studies

There was poor reporting of the participant voice in some of the included studies. For example, many studies included limited first‐order constructs or data extracts, and these were often not labelled with an identifier of the participant. We also found poor reporting of researcher reflexivity across many of the studies, which limited transparency regarding the role of the researcher. All studies gave some description, even if very brief, of the context, participants, sampling, methods, and analysis.

### Confidence in the findings

Based on our CERQual assessments, we had high confidence in four findings and moderate confidence in nine findings, indicating that the studies were a good representation of the phenomenon of interest. We had several findings where we had low (nine) or very low confidence (three), indicating that the studies were a weaker fit with the representation of the phenomenon of interest. Our main concerns were connected to the methodological limitations of the studies and the relevance and adequacy of the data. Common methodological limitations included a lack of researcher reflexivity as well as poor reporting of ethical considerations, sampling, and representation of the participant’s voice in the findings. The data were often assessed as being only partially relevant, mainly because the included studies represented few regions; had a focus on a certain target population (e.g. youth) or a specific topic (HIV/AIDS); or because many of the included studies explored participants’ perceptions of hypothetical situations or digital health interventions, or both. Finally, our concerns about adequacy were mainly tied to the limited number of studies included in some findings and the thinness of the data contributing to some findings.

The GRADE‐CERQual evidence profile tables supporting the assessment of confidence in each finding can be found in Appendix 4. We start each section of the findings with a link to the 'CERQual summary of qualitative findings' table where a summary assessment of the findings from that section is presented.

### Findings and categories identified in the data

From our synthesis, we developed a set of individual findings, and then organised these findings into six overarching categories related to (1) the general acceptability of and preferences around digital health interventions; (2) the varying degrees of access to network services, phones, and messages; (3) communication delivery and format preferences; (4) communication content preferences; (5) privacy and confidentiality regarding personal health information; and (6) perceptions of intervention impact. Unless specifically addressed in the detailed finding, the data were not specific to HIC or LMIC setting or to a specific client group. For a description of the context and client group in each study contributing to a finding, please refer to the evidence profiles in Appendix 4.

#### General acceptability of and preferences around digital targeted client communication

('Summary of qualitative findings' table for Findings 1 to 4 is shown in Table 2.)

**2:** 'Summary of qualitative findings' table for findings related to general acceptability of and preferences around digital health interventions

**Finding**		**Overall CERQual assessment**	**Explanation for assessment**	**Contributing studies**
**1**	Overall, participants had a range of views regarding acceptance of the idea of receiving health information through their mobile devices. This was due to factors such as familiarity with the technology, convenience, control, being able to save and re‐read messages later, cost, seeing it as a simple way of providing a reminder for medication or appointments, and the sense that someone was thinking about them and cared enough to send a message	Low confidence	Due to moderate concerns regarding methodological limitations and relevance	Akinfaderin‐Agarau 2012; Brown 2014; Calderón 2017; Cates 2015; Cornelius 2009; Curioso 2009; Evans 2016; French 2016; Gold 2010; Greaney 2014; Hirsch‐Moverman 2017; Jalloh‐Vos 2014; Jennings 2013; Lau 2014; Mbuagbaw 2012; Mbuagbaw 2014; Menacho 2013; Missal 2016; Munro 2017; Naughton 2013; Odeny 2014; Perry 2012; Rana 2015; Rodrigues 2015; Sloan 2017; Smillie 2014; Smith 2017; Willoughby 2017; Wright 2011
**2**	In discussing the pros and cons of digital targeted client communication compared to in‐person meetings with a healthcare provider, some participants perceived interacting with a healthcare provider as preferable, warmer, and something to which they were accustomed. Others also felt that people could receive a faster response using digital communication and that the messages were more convenient and less judgemental. However, some liked having direct access to both healthcare providers and digital targeted client communication	Very low confidence	Due to minor concerns regarding methodological limitations and serious concerns regarding adequacy and relevance	Calderón 2017; Nachega 2016; Naughton 2013; Sloan 2017; Smillie 2014
**3**	Participants said that they liked two‐way digital communication, as this allowed them to engage directly with a healthcare provider, which they trusted more; to receive answers to their questions and have opportunities for discussion; and to receive a more immediate response. However, some participants felt that for some topics they would feel uncomfortable talking to a healthcare provider through a digital channel due to issues related to shyness and privacy, and would prefer to use SMS	Very low confidence	Due to moderate concerns regarding methodological limitations and adequacy and serious concerns regarding relevance	Akinfaderin‐Agarau 2012; Calderón 2017; Cates 2015; Jennings 2013; Rana 2015; Rodrigues 2015; Smillie 2014; Smith 2017; Willoughby 2017
**4**	Some participants expressed a concern that some people might view digital targeted communication from healthcare providers as a replacement for seeking appropriate medical assistance, which might have adverse impacts. While some saw digital health as a way to increase access to care, others noted that text messaging might be seen by poorer people as a cheaper or sufficient healthcare option, which might decrease appropriate health‐seeking behaviour	Very low confidence	Due to serious concerns regarding relevance and adequacy	Willoughby 2017

**Finding 1: Overall, participants had a range of views regarding acceptance of the idea of receiving health information through their mobile devices. This was due to factors such as familiarity with the technology, convenience, control, being able to save and re‐read messages later, cost, seeing it as a simple way of providing a reminder for medication or appointments, and the sense that someone was thinking about them and cared enough to send a message (low confidence in the evidence).**

Many studies from a variety of contexts and client groups presented data related to the range of participants’ views regarding the acceptance of DTCC and the factors that influenced this acceptance (Akinfaderin‐Agarau 2012; Brown 2014; Calderón 2017; Cates 2015; Cornelius 2009; Curioso 2009; Evans 2016; French 2016; Gold 2010; Greaney 2014; Hirsch‐Moverman 2017; Jalloh‐Vos 2014; Jennings 2013;Lau 2014; Mbuagbaw 2012; Mbuagbaw 2014; Menacho 2013; Missal 2016; Munro 2017; Naughton 2013; Odeny 2014; Perry 2012; Rana 2015; Rodrigues 2015; Sloan 2017; Smillie 2014; Smith 2017; Willoughby 2017; Wright 2011). Many participants had not used mobile devices to access health information previously, but were open to and interested in the idea of digital health interventions being used to deliver up‐to‐date knowledge and information or reminders for appointments or medication (Akinfaderin‐Agarau 2012; Cates 2015; Evans 2016; Gold 2010; Greaney 2014; Hirsch‐Moverman 2017; Jennings 2013; Lau 2014; Mbuagbaw 2014; Odeny 2014). Some participants felt that it was more personal than other methods of delivering health information such as posters and billboards (Gold 2010). Others perceived it as a way of boosting already‐existing interventions or curricula, such as school‐based HIV curricula (Cornelius 2009).

However, some participants felt that DTCC delivered via mobile device were not acceptable for their setting and chose not to participate in the interventions. An example of this was seen in a family planning intervention in Sierra Leone where some husbands had a problem with their partner being called by the healthcare provider or their partner participating in the family planning intervention, or both (Jalloh‐Vos 2014). Others felt that in some situations it was important to still have the opportunity to speak to a person on the phone or in person (face‐to‐face), for example if they were having a strong craving for a cigarette and needed support in that moment (Naughton 2013). Some participants felt that DTCC would be useful for certain population groups such as younger audiences (French 2016; Willoughby 2017; Wright 2011), youth just starting on HIV medication, patients not adhering to medication, and those with less education (Smith 2017).

Familiarity with technology, especially SMS, was one reason that participants put forward for accepting digital health interventions (Brown 2014; Cornelius 2009; French 2016; Jennings 2013; Smillie 2014). Youth and pregnant women described using digital technology as something they already did frequently, that fit their learning styles, and was not a foreign approach (Brown 2014; Cornelius 2009; French 2016; Munro 2017). Others mentioned that they were already using SMS to request healthcare providers to call them back or to set up health appointments (Jennings 2013).

Participants also noted that messages delivered through digital mechanisms were very convenient and in some cases cost‐effective. Some felt that they would be very beneficial for families who lived a long distance from the health facility and would save them time and money (Calderón 2017). Messages were perceived as being quick and much easier than going to the health facility to get a pamphlet, going to a doctor’s appointment (Calderón 2017; Jennings 2013; Smillie 2014; Smith 2017), or searching for information online or in books (Lau 2014). Messages were perceived as easily accessible, providing immediate guidance or support, and not taking up much time or attention (French 2016; Munro 2017; Naughton 2013; Perry 2012; Sloan 2017). Clients also liked the fact that the messages were often free. Challenges to convenience included the cost of receiving messages and phone calls and the difficulty of maintaining privacy and confidentiality in some settings when discussing sensitive information via call or SMS (Jennings 2013; Perry 2012).

Participants liked that they could save and re‐read messages as well as have control over receiving, keeping, or deleting the information (Brown 2014; Evans 2016; French 2016; Munro 2017). Others felt that the intervention gave them some control over their own care and health information (Jennings 2013; Munro 2017).

Some participants felt that DTCC delivered via a mobile device was an acceptable way of providing reminders for medication taking or appointments (Curioso 2009; Lau 2014; Mbuagbaw 2012; Mbuagbaw 2014; Rana 2015; Rodrigues 2015). However, others felt that these reminders were not necessary and could be detrimental to patient independence. Some participants only wanted them sent to patient groups who needed help adhering to medication or when they were preoccupied or fatigued (Hirsch‐Moverman 2017; Mbuagbaw 2014; Rana 2015; Rodrigues 2015).

Finally, some participants liked DTCC that delivered messages to their mobile phones, experiencing it as supportive and making them feel that someone was thinking of and cared about them (Greaney 2014; Lau 2014; Munro 2017; Naughton 2013; Rana 2015; Sloan 2017).

**Finding 2: In discussing the pros and cons of DTCC compared to in‐person meetings with a healthcare provider, some participants perceived interacting with a healthcare provider as preferable, warmer, and something to which they were accustomed. Others also felt that people could receive a faster response using digital communication and that the messages were more convenient and less judgemental. However, some liked having direct access to both healthcare providers and DTCC (very low confidence).**

A few studies, from both LMIC and HIC contexts (Calderón 2017; Nachega 2016; Naughton 2013; Sloan 2017; Smillie 2014), described a range of participants’ preferences for digital health interventions compared to in‐person visits to healthcare providers. The majority of studies in this finding looked at the perspectives of pregnant and postpartum women and parents. Some clients liked having direct access to both healthcare providers and to digital health interventions, as each played a different role (Naughton 2013; Sloan 2017; Smillie 2014). Some felt that the digital health interventions were more convenient, reliable, flexible, and faster and provided more frequent support (Nachega 2016; Naughton 2013; Sloan 2017; Smillie 2014). Clients who were pregnant and trying to quit smoking often preferred the SMS interventions, as they felt healthcare providers judged them and made them feel uncomfortable (Sloan 2017). Clients in some studies liked the digital health interventions but still felt it was important to have access to in‐person visits with healthcare providers or speaking with someone when needed (Calderón 2017; Naughton 2013; Smillie 2014).

**Finding 3: Participants said that they liked two‐way digital communication as this allowed them to engage directly with a healthcare provider, which they trusted more; to receive answers to their questions and have opportunities for discussion; and to receive a more immediate response. However, some participants felt that for some topics they would feel uncomfortable talking to a healthcare provider through a digital channel, due to issues related to shyness and privacy, and would prefer to use SMS (very low confidence).**

Some studies from both LMIC and HIC contexts found that participants wanted or liked to have the option of engaging directly with healthcare providers through DTCC in order to receive answers to their questions (Akinfaderin‐Agarau 2012; Calderón 2017; Cates 2015; Jennings 2013; Rana 2015; Rodrigues 2015; Smillie 2014; Smith 2017; Willoughby 2017). In general, participants felt that these types of two‐way communication options would be useful and provide them with answers to their questions when they needed them, as well as allow them to maintain contact with their healthcare providers in between appointments if questions or concerns were to arise (Rana 2015). One participant in one study stated that two‐way communication would not be acceptable, as the person on the other end would then be informed of his HIV status (Rodrigues 2015).

Some participants preferred voice calls for engaging with healthcare providers above communicating with them through SMS (Akinfaderin‐Agarau 2012). There were different reasons for this. Some participants felt that they could ask detailed questions and receive detailed answers as well as discuss the various problems or challenges they were facing (Akinfaderin‐Agarau 2012; Rodrigues 2015), or that the service could be more trusted because they spoke to someone directly (Akinfaderin‐Agarau 2012). Others thought that it would provide more opportunity for discussion and ensure that the message was well received by the intended recipient, and that they could receive an immediate response (Jennings 2013).

However, some participants explained that they preferred SMS services, as they would allow the participants to be more open and to ask about issues they would be too shy to bring up when speaking directly with someone (Akinfaderin‐Agarau 2012; Smillie 2014). Some participants felt that they would not feel comfortable talking to someone in person, especially if that person was new or unknown to them (Smillie 2014). SMS was also viewed as advantageous for brief and relatively confidential receipt of information (Jennings 2013).

**Finding 4: Some participants expressed a concern that some people might view digital targeted communication from healthcare providers as a replacement to seeking appropriate medical assistance, which might have adverse impacts. While some saw digital health as a way to increase access to care, others noted that text messaging might be seen by poorer people as a cheaper or sufficient healthcare option, which might decrease appropriate health‐seeking behaviour (very low confidence).**

Participants in one study from the USA exploring college students' views on receiving SMS for sexual health promotion expressed concern that other people might become over‐reliant on digital health interventions because they were seen as a cheaper option than going to the doctor (Willoughby 2017). There was a worry that people would use the digital communication intervention instead of seeking appropriate medical attention. The participants thought that this could especially be the case for people with few resources.

#### Varying degrees of access to network services, phones, and messages

('Summary of qualitative findings' table for Findings 5 to 9 is shown in Table 3.)

**3:** 'Summary of qualitative findings' table for findings related to the varying degrees of access to network services, phones, and messages

**Finding **		**Overall CERQual assessment **	**Explanation for assessment **	**Contributing studies**
**5**	Participants reported varying degrees of access to network services, including cell networks (for calls and SMS) and internet. In addition, some had poor access to electricity to charge their phones. These factors were reported to be barriers to using the digital targeted client communication.	High confidence	Due to minor concerns regarding methodological limitations	Akinfaderin‐Agarau 2012; Cornelius 2009; Flax 2017; Hirsch‐Moverman 2017; Jalloh‐Vos 2014; Mbuagbaw 2012; Mbuagbaw 2014; Smillie 2014
**6**	Participants reported varying degrees of access to mobile devices. For instance, some had no phone; some had lost or broken their phone; some could not afford to purchase airtime; some had changed their number or sim card; or for some access to the phone was controlled by another person. These factors were reported to be barriers to using the digital targeted client communication.	Moderate confidence	Due to minor concerns regarding methodological limitations and relevance	Akinfaderin‐Agarau 2012; Entsieh 2015; Flax 2017; Hirsch‐Moverman 2017; Jalloh‐Vos 2014; Jennings 2013; Menacho 2013; Missal 2016; Rana 2015; Smillie 2014
**7**	Some participants, particularly women and adolescents, had their access to phones controlled or restricted by others, especially if they had to share or borrow a phone. They noted that they would often have to explain why they wanted to use the phone, and who they wanted to call, to allay suspicions about this communication. They mentioned that this was a barrier to accessing digital targeted client communication and made it difficult to keep their messages private.	Moderate confidence	Due to minor concerns regarding methodological limitations, coherence, adequacy, and relevance	Akinfaderin‐Agarau 2012; Flax 2017; Jalloh‐Vos 2014; Rana 2015
**8**	Participants believed that the cost of participating in digital targeted client communication should be free or very low, as cost could present a barrier to participation, particularly for young people and those on lower incomes. Participants felt that there should be little or no charge for costs such as joining the digital health intervention, downloading applications (apps), or for sending and receiving mobile messages/phone calls.	High confidence	Due to minor concerns regarding relevance	Akinfaderin‐Agarau 2012; Calderón 2017; Cornelius 2009; Menacho 2013; Mitchell 2016; Perry 2012; Rana 2015; Smith 2017
**9**	Participants’ ability to access digital communication was sometimes limited by their language skills and their personal level of literacy or techno‐literacy, or both.	Moderate confidence	Due to minor concerns regarding relevance and moderate concerns regarding methodological limitations	Akinfaderin‐Agarau 2012; Calderón 2017; Curioso 2009; Greaney 2014; Hirsch‐Moverman 2017; Jalloh‐Vos 2014; Mbuagbaw 2014; Rodrigues 2015; Smillie 2014

**Finding 5: Participants reported varying degrees of access to network services, including cell networks (for calls and SMS) and the internet. In addition, some participants had poor access to electricity to charge their phones. These factors were reported to be barriers to using the DTCC (high confidence).**

Studies from a range of income settings found that issues related to network services and electricity acted as a barrier to people’s use of DTCC (Akinfaderin‐Agarau 2012; Cornelius 2009; Flax 2017; Hirsch‐Moverman 2017; Jalloh‐Vos 2014; Mbuagbaw 2012; Mbuagbaw 2014; Smillie 2014). Lack of network or internet coverage meant that some participants could not participate in the intervention or did not receive some of the messages (Akinfaderin‐Agarau 2012; Cornelius 2009; Flax 2017; Hirsch‐Moverman 2017; Jalloh‐Vos 2014; Mbuagbaw 2012; Mbuagbaw 2014; Smillie 2014). For instance, when network coverage was poor, some participants in a Nigerian study recommended that SMS was the best option as they were more likely to be transmitted when the network was unstable, whereas voice calls would not connect or would be dropped (Akinfaderin‐Agarau 2012). Participants in a study from Canada described living in mountainous areas with no network coverage (Smillie 2014). Participants in low‐income settings also mentioned that not being able to charge their phone due to power outages or lack of access to electricity was a barrier to participating in digital health interventions (Akinfaderin‐Agarau 2012; Hirsch‐Moverman 2017; Mbuagbaw 2014).

**Finding 6: Participants reported varying degrees of access to mobile devices. For instance, some participants had no phone; some had lost or broken their phone; some could not afford to purchase airtime; some had changed their number or sim card; or for some access to the phone was controlled by another person. These factors were reported to be barriers to using the DTCC (moderate confidence).**

Some studies, the majority from LMIC settings in Africa (Akinfaderin‐Agarau 2012; Entsieh 2015; Flax 2017; Hirsch‐Moverman 2017; Jalloh‐Vos 2014; Jennings 2013; Menacho 2013; Missal 2016; Rana 2015; Smillie 2014), found that access to functioning mobile phones was a barrier to participants’ use of DTCC. Some participants reported not owning a phone (Akinfaderin‐Agarau 2012; Hirsch‐Moverman 2017; Jalloh‐Vos 2014; Rana 2015), and others had lost or broken their phone (Flax 2017; Smillie 2014). For some, not owning a phone caused feelings of jealousy and unhappiness and forced them to borrow a phone if they wanted to participate in the digital health intervention (Jalloh‐Vos 2014).

Cost was also a barrier to participation for some participants, as they could not afford the airtime or credit needed to receive or send SMS or phone calls (Jalloh‐Vos 2014; Jennings 2013; Smillie 2014), although in some studies, participants received free airtime, which removed this access barrier (Hirsch‐Moverman 2017). In other cases, participants changed residence, changed sim cards, or had multiple sim cards and were no longer able to be reached by the DTCC (Missal 2016; Rana 2015; Smillie 2014).

For some participants, their access to a phone was controlled by others. This could be because they could not afford to purchase a phone themselves (Jalloh‐Vos 2014), or because physical access to the mobile phone was controlled by another person (see Finding 18) (Akinfaderin‐Agarau 2012; Jalloh‐Vos 2014; Rana 2015). This group was mainly comprised of women and adolescents, and this is discussed further in Findings 7 and 18.

**Finding 7: Some participants, particularly women and adolescents, had their access to phones controlled or restricted by others, especially if they had to share or borrow a phone. They noted that they would often have to explain why they wanted to use the phone, and who they wanted to call, to allay suspicions about this communication. They mentioned that this was a barrier to accessing DTCC and made it difficult to keep their messages private (moderate confidence).**

A few studies from LMIC settings in Africa found that some participants, particularly women and adolescents, had their access to phones controlled or restricted by others, especially if they had to share or borrow a phone (Akinfaderin‐Agarau 2012; Flax 2017; Jalloh‐Vos 2014; Rana 2015). They mentioned that this was a barrier to accessing DTCC and made it difficult to keep their messages private. In some contexts, women had their mobile phone use controlled by their husbands or other family members. The women would often have to explain why they wanted to use the phone and who they wanted to call (Akinfaderin‐Agarau 2012; Jalloh‐Vos 2014; Rana 2015), for example to allay suspicion that they were talking to their boyfriends or having an affair. For some women, this would mean having to find an alternative phone to use if they did not want their husband to know they were using a digital health service. For example, in one study, some women did not want their husbands knowing they were receiving information on family planning (Jalloh‐Vos 2014). In some settings, women and girls were also viewed by their society as not having time to use phones due to greater domestic obligations than their male counterparts (Akinfaderin‐Agarau 2012). Youth in one study also reported facing restrictions related to using phones at school (Rana 2015).

In one study, women in a women’s group all shared a single phone. They elected one group member to control the phone and share the messages. This group member was then responsible for distributing the messages from the DTCC. In this context, the majority of the participants accepted this form of phone sharing and believed it was functional (Flax 2017).

In all studies contributing to this finding, participants felt that sharing a phone or having access to their phone controlled by someone else delayed the delivery of the message (Flax 2017; Rana 2015), and decreased the privacy and confidentiality around their personal information (Akinfaderin‐Agarau 2012; Jalloh‐Vos 2014; Rana 2015).

**Finding 8: Participants believed that the cost of participating in DTCC should be free or very low, as cost could present a barrier to participation, particularly for young people and those on lower incomes. Participants felt that there should be little or no charge for costs such as joining the digital health intervention, downloading applications (apps), or for sending and receiving mobile messages/phone calls (high confidence).**

Participants in several studies felt it was important for digital health interventions to have little or no cost, as these costs could present a barrier to participation (Akinfaderin‐Agarau 2012; Calderón 2017; Cornelius 2009; Menacho 2013; Mitchell 2016; Perry 2012; Rana 2015; Smith 2017). This was especially important to young people (Akinfaderin‐Agarau 2012; Perry 2012), and those with lower incomes (Calderón 2017; Cornelius 2009; Mitchell 2016; Rana 2015). If the intervention could not be offered at no cost to the client, then participants felt that the interventions should be very low cost and that cheaper options should be used, for example SMS instead of voice calls (Akinfaderin‐Agarau 2012). In some cases, messages sent to participants were free, but if a participant wanted to reply they had to use their own airtime. Some participants thought that this would prevent people from using the bi‐directional functions within digital health interventions (Rana 2015).

**Finding 9: Participants’ ability to access digital communication was sometimes limited by their language skills and their personal level of literacy and/or techno‐literacy (moderate confidence).**

Some studies, the majority from LMIC settings (Akinfaderin‐Agarau 2012; Calderón 2017; Curioso 2009; Greaney 2014; Hirsch‐Moverman 2017; Jalloh‐Vos 2014; Mbuagbaw 2014; Rodrigues 2015; Smillie 2014), found that participants’ ability to access digital health messages was sometimes limited by their language skills (Akinfaderin‐Agarau 2012; Jalloh‐Vos 2014; Mbuagbaw 2014; Smillie 2014), or not understanding how to use the technology (Calderón 2017; Curioso 2009; Hirsch‐Moverman 2017; Jalloh‐Vos 2014; Rodrigues 2015; Smillie 2014). One study from the USA found that Latina women needing cancer screening receiving interactive voice recordings as reminders understood how to access messages but that the language used in the messages was not familiar to them (Greaney 2014), as illustrated in the following quote.

“'If your response is ‘yes’, then press the star button.’ ‘Star’ is what Americans say, but on the telephone there is no star; it is an asterisk. It all depends on who you are speaking with. If the person you are speaking with understands that that is a star then let’s press the star, but if the person understands it’s an asterisk then he/she will begin to look for a star” (Greaney 2014).

Participants with literacy issues often preferred voice calls to SMS, as they could talk with the caller and ask for clarifying information (Akinfaderin‐Agarau 2012; Jalloh‐Vos 2014). Participants in two studies said they had learned or were willing to learn how to text in order to participate in digital health interventions (Calderón 2017; Smillie 2014).

#### Communication delivery and format preferences

('Summary of qualitative findings' table for Findings 10 to 13 is shown in Table 4.)

**4:** 'Summary of qualitative findings' table for findings related to communication delivery and format preferences

**Finding**		**Overall CERQual assessment**	**Explanation for assessment**	**Contributing studies**
**10**	Participants often had preferences for how often health messages were sent, the time of day they were sent, and the duration of the digital targeted client communication. However, there was variation in what most participants felt was appropriate timing and frequency, and these preferences were often linked to the health issue on which the messaging was focused; whether people had their own phone or had to share a phone; and the participant’s particular circumstances. Participants were particularly concerned about being bombarded with too many messages; whether the timing of the messages was convenient for them; and/or whether messages arrived in connection with the behaviour the message was trying to target.	Moderate confidence	Due to minor concerns regarding methodological limitations and moderate concerns regarding relevance	Calderón 2017; Cornelius 2009; Evans 2016; French 2016; Gold 2010; Greaney 2014; Jennings 2013; Mbuagbaw 2012; Menacho 2013; Missal 2016; Mitchell 2016; Munro 2017; Naughton 2013; Odeny 2014; Rana 2015; Rodrigues 2015; Sloan 2017; Smillie 2014; Smith 2017; Ware 2016; Willoughby 2017; Wright 2011
**11**	Participants had different preferences for various delivery channels available for sharing information through digital targeted client communication, including mobile messaging, interactive voice response, or speaking with a healthcare provider. These preferences were influenced by a number of factors including cost, convenience, the ability to store messages and re‐read them, familiarity with the channel, personal preferences, the nature of the content being delivered, the nature of the topic, language and literacy considerations, and the ability to have a discussion with a real‐life person.	Moderate confidence	Due to minor concerns regarding methodological limitations and moderate concerns regarding relevance	Akinfaderin‐Agarau 2012; Cates 2015; Curioso 2009; Greaney 2014; Jennings 2013; Missal 2016; Mitchell 2016; Naughton 2013; Odeny 2014; Rana 2015; Rodrigues 2015; Smillie 2014; Willoughby 2017
**12**	Participants appreciated personalised health information and discussed their preferences for options to make interventions more relevant to individuals. This could include sender‐based personalisation or receiver‐based options. Reasons for these preferences included engaging the user, enhancing credibility, increasing feelings of ownership, control over their personal information and feelings of privacy. Preferences for tailoring included making digital health messages personalised by using an individual's name; allowing participants to choose the content, topic, and language of their messages; providing information relevant to the participant's setting (local information); allowing them to select the timing and frequency of the message; providing personalised reminders (e.g. for vaccination or medication); and allowing participants to have control over privacy settings.	Low confidence	Due to minor concerns regarding methodological limitations and serious concerns regarding relevance	Calderón 2017; Evans 2016; French 2016; Goldenberg 2015; Hirsch‐Moverman 2017; Jennings 2013; Munro 2017; Naughton 2013; Odeny 2014; Sloan 2017; Ware 2016; Willoughby 2017
**13**	Participants mentioned various message formats that they preferred. These included a preference for short, concise, personalised, clear, and direct messages in a language they could understand and in full text rather than "text speak".	Low confidence	Due to minor concerns regarding methodological limitations and serious concerns regarding relevance	Akinfaderin‐Agarau 2012; Calderón 2017; Cates 2015; Curioso 2009; Evans 2016; French 2016; Gold 2010; Greaney 2014; Lau 2014; Menacho 2013; Missal 2016; Munro 2017; Naughton 2013; Odeny 2014; Perry 2012; Rana 2015; Smillie 2014; Willoughby 2017

**Finding 10: Participants often had preferences for how often health messages were sent, the time of day they were sent, and the duration of the DTCC. However, there was variation in what most participants felt was appropriate timing and frequency, and these preferences were often linked to the health issue on which the messaging was focused; whether people had their own phone or had to share a phone; and the participant’s particular circumstances. Participants were particularly concerned about being bombarded with too many messages; whether the timing of the messages was convenient for them; and/or whether messages arrived in connection with the behaviour the message was trying to target (moderate confidence).**

Many studies discussed and presented participants’ preferences related to timing, frequency of messages, and duration of digital health projects (Calderón 2017; Cornelius 2009, Evans 2016; French 2016; Gold 2010; Greaney 2014; Jennings 2013; Mbuagbaw 2012; Menacho 2013; Missal 2016; Mitchell 2016; Munro 2017; Naughton 2013; Odeny 2014; Rana 2015; Rodrigues 2015; Sloan 2017; Smillie 2014; Smith 2017; Ware 2016; Willoughby 2017; Wright 2011). However, within and across studies and client groups, there was no consensus as to the ideal timing, frequency, or duration, as this was linked to personal preferences, contextual factors (such as attending school), and the behaviour or information the text message was trying to target.

With regard to frequency, participants did not want in general to feel pestered or bombarded by too many messages (Evans 2016; French 2016; Gold 2010; Willoughby 2017), but described a fine balance between feeling bombarded and not receiving enough information to reinforce the messages. For example, participants receiving medication reminders were open to receiving multiple texts a day, whereas those receiving more general/less tailored information wanted messages much less frequently. However, in a number of studies no clear consensus emerged on the optimal frequency of the messages (Evans 2016; Gold 2010; Rana 2015).

Participants' preferences for message frequency could also be linked to owning a phone or having to share (Jennings 2013). Those who had to share a phone wanted messages only a few times a week with the ability to stop the messages if the phone owner was away, whereas those who owned their own phone were open to daily messages (Jennings 2013).

With regard to the timing of delivery of the messages, preferences for timing varied among population groups. It was important to participants that messages arrive when they could be seen and accessed, for example not late at night, when they would not be seen until the next day (Rana 2015; Ware 2016). Some adolescents and young adults thought during school hours would be fine (Cornelius 2009; French 2016), whereas others believed that this could cause problems based on restrictions around phone use (Cornelius 2009; Rana 2015; Wright 2011). Some participants felt that it was important that the message arrive in connection with the behaviour it was targeting (French 2016; Gold 2010; Menacho 2013; Naughton 2013; Rana 2015; Ware 2016), or in good time before an appointment (Odeny 2014), for example on a Friday night before going out to remind them of condom use (French 2016). Participants also liked the option of tailoring the timing of messages to fit their lives (French 2016).

With regard to duration, no consensus emerged on how long the intervention should last (Evans 2016).

**Finding 11: Participants had different preferences for various delivery channels available for sharing information through DTCC, including mobile messaging, interactive voice response, or speaking with a healthcare provider. These preferences were influenced by a number of factors including cost, convenience, the ability to store messages and re‐read them, familiarity with the channel, personal preferences, the nature of the content being delivered, the nature of the topic, language and literacy considerations, and the ability to have a discussion with a real‐life person (moderate confidence).**

Some studies presented data related to participants’ preferences for the delivery channel used to share information for digital health interventions (Akinfaderin‐Agarau 2012; Cates 2015; Curioso 2009; Greaney 2014; Jennings 2013; Mitchell 2016; Missal 2016; Naughton 2013; Odeny 2014; Rana 2015; Rodrigues 2015; Smillie 2014; Willoughby 2017). Some participants believed that different delivery channels would meet different needs and have different purposes (Willoughby 2017).

Participants from a number of studies had a preference for information delivered by SMS (Akinfaderin‐Agarau 2012; Curioso 2009; Jennings 2013; Mitchell 2016; Naughton 2013; Odeny 2014; Rana 2015; Smillie 2014). Reasons for preferring SMS included lower cost for the participant (Akinfaderin‐Agarau 2012); the messages were brief (Jennings 2013; Naughton 2013); confidential and protected privacy (Akinfaderin‐Agarau 2012; Smillie 2014); could be kept for reference and re‐reading later (Curioso 2009; Jennings 2013); were easier to share (Akinfaderin‐Agarau 2012; Naughton 2013); and to understand, for example due to literacy issues or not understanding the accent of the person calling (Akinfaderin‐Agarau 2012; Smillie 2014).

However, participants in a few studies raised concerns with text messaging or preferred other delivery channels. They thought, for example, that text messages were more appropriate for younger audiences (Willoughby 2017), or that the highly convenient nature of the SMS was a negative that would only have a short‐term impact, as people would delete them or ignore them (Naughton 2013; Rana 2015). Finally, others who were unfamiliar with SMS technology felt that they were too passive and unfamiliar and so they ignored them (Rodrigues 2015).

A few studies presented data that participants preferred interactive voice response (IVR) to receive digital health interventions (Greaney 2014; Missal 2016; Rodrigues 2015). Often this was related to low literacy levels in a community or that participants found them easier, more interactive, and that they attracted more attention than an SMS, meaning that they would not be easily missed (Rodrigues 2015). However, some participants felt that they should be able to repeat or re‐read the message at a time more convenient to them, for example through linked delivery on platforms such as voice, video, or SMS (Missal 2016).

A number of studies presented data where participants discussed their preferences for speaking directly with a healthcare provider on the phone to other delivery platforms (Akinfaderin‐Agarau 2012; Greaney 2014; Jennings 2013; Odeny 2014; Rana 2015; Rodrigues 2015). Participants talked about why they preferred speaking directly to a healthcare provider. These reasons included that phone calls were clear and could aid understanding in people with low literacy levels (Akinfaderin‐Agarau 2012); they trusted speaking to a healthcare provider (Akinfaderin‐Agarau 2012); they could engage in discussions (Jennings 2013); and they felt it was faster and more immediate to receive an answer to their questions (Akinfaderin‐Agarau 2012; Jennings 2013; Rodrigues 2015).

Participants also discussed other delivery channels that they liked. These included an app (Mitchell 2016), written letters (Greaney 2014), voicemails, Facebook, and reminder alarms (Rana 2015).

**Finding 12: Participants appreciated personalised health information and discussed their preferences for options to make interventions more relevant to individuals. This could include sender‐based personalisation or receiver‐based options. Reasons for these preferences included engaging the user, enhancing credibility, increasing feelings of ownership, control over their personal information, and feelings of privacy. Preferences for tailoring included making digital health messages personalised by using an individual's name; allowing participants to choose the content, topic, and language of their messages; providing information relevant to the participant's setting (local information); allowing them to select the timing and frequency of the message; providing personalised reminders (e.g. for vaccination or medication); and allowing participants to have control over privacy settings (low confidence).**

Some studies discussed participants’ thoughts about personalised or customised messaging and their preferences for options to make interventions more relevant to individuals (Calderón 2017; Evans 2016; French 2016; Goldenberg 2015; Hirsch‐Moverman 2017; Jennings 2013; Munro 2017; Naughton 2013; Odeny 2014; Sloan 2017; Ware 2016; Willoughby 2017). Some participants felt that if the messages were not tailored to individual users this could cause them to disengage from interventions or cause problems in their personal lives (Jennings 2013; Munro 2017; Naughton 2013, ). For example, participants in one study on HIV messaging in Kenya to prevent mother‐to‐child transmission suggested that two different sets of messages be developed for those who had and those who had not disclosed their HIV status to their partner. This would help women avoid risking disclosure of their status (Jennings 2013). Participants in another study discussed the importance of using personal pronouns to make messages more relevant to the user. They felt that naming functions within the digital health intervention as, for example, “ MY test plan”, would help participants to take ownership of these functions (Goldenberg 2015).

Participants in several studies mentioned ways in which the people receiving communication could personalise or customise digital health interventions that they believed would be important. These included the following.

- Being able to request the time of day the message(s) would be sent (French 2016; Hirsch‐Moverman 2017; Jennings 2013; Sloan 2017; Ware 2016; Willoughby 2017).
- Being able to select the frequency of the messages (Hirsch‐Moverman 2017; Willoughby 2017).
- Being able to customise the app to meet personal needs, such as reminders and privacy settings (Goldenberg 2015).
- Being able to select a preferred language (Jennings 2013; Ware 2016).
- Being able to personalise or select message content (Jennings 2013; Sloan 2017; Ware 2016; Willoughby 2017).

Participants in a number of studies mentioned ways in which the people designing and sending communication could personalise or customise digital health interventions that they believed would be important. These included the following.

- Using unique access codes in order to prevent children and other adults from unintentionally gaining entry into discussed personal health information (Calderón 2017).
- Using the individual’s name (Evans 2016).
- Delivering the message in different languages (Evans 2016).
- Including community‐specific information (Evans 2016; Munro 2017).
- Being able to, for example, text “STOP” if they wanted to stop receiving messages (French 2016).
- Explaining the reason for the appointment as well as the date (e.g. the specific vaccinations to be received on that day) (Odeny 2014).

**Finding 13: Participants mentioned various message formats that they preferred. These included a preference for short, concise, personalised, clear, and direct messages in a language they could understand and in full text rather than "text speak" (low confidence).**

A number of studies presented data related to participants’ preferences for message format (Akinfaderin‐Agarau 2012; Calderón 2017; Cates 2015; Curioso 2009; Evans 2016; French 2016; Gold 2010; Greaney 2014; Lau 2014; Menacho 2013; Missal 2016; Munro 2017; Naughton 2013; Odeny 2014; Perry 2012; Rana 2015; Smillie 2014; Willoughby 2017). In general, participants liked or wanted short and concise messages that were easy to understand and factual, especially from text messages (Calderón 2017; Curioso 2009; Evans 2016; French 2016; Gold 2010; Greaney 2014; Lau 2014; Menacho 2013; Odeny 2014; Perry 2012; Rana 2015; Wright 2011). Most participants preferred text messages that were written out in full and did not use abbreviations or slang, as these felt more professional and were more representative of how they thought a health professional would write (Cates 2015; French 2016; Naughton 2013; Willoughby 2017).

In one study (Missal 2016), participants commented on their experiences with interactive voice response (IVR). Most found the content and language of the message useful. However, some found the message too fast, short, and sometimes the voice was not clear enough. They were unable to request for the message to be played again if they had missed or misunderstood the content.

#### Communication content preferences

('Summary of qualitative findings' table for Fndings 14 to 17 is shown in Table 5.)

**5:** 'Summary of qualitative findings' table for findings related to communication content preferences

**Finding**		**Overall CERQual assessment**	**Explanation for assessment**	**Contributing studies**
**14**	Participants’ perceptions of who sent the digital health communication could influence their trust in and perception of the credibility and value of the digital targeted client communication and the information it provides. Participants said they wanted a known, identified phone number; messages sent from a reliable, trusted, credible source such as health professionals or official sources; and in some cases to feel like the messages were sent by a person (even if sent from an automated service). However, some participants, such as those with stigmatised health conditions, preferred an unmarked sender to protect their privacy.	Moderate confidence	Due to minor concerns regarding methodological limitations and moderate concerns regarding relevance	Akinfaderin‐Agarau 2012; Brown 2014; Calderón 2017; Cates 2015; Evans 2016; Greaney 2014; Lau 2014; Mbuagbaw 2012; Menacho 2013; Missal 2016; Naughton 2013; Rana 2015; Rodrigues 2015; Smillie 2014; Willoughby 2017
**15**	Participants said that the tone of digital health communication mattered to them. Their preferences varied but included a tone that was: motivational, friendly, encouraging, polite, respectful, congratulatory, personalised, upbeat, positive, humorous, and relatable. Some participants highlighted that they did not like feeling pressured, lectured to, shamed, or frightened by digital health messages.	Low confidence	Due to minor concerns regarding methodological limitations and serious concerns regarding relevance	Cates 2015; Curioso 2009; Evans 2016; French 2016; Gold 2010; Jennings 2013; Menacho 2013; Munro 2017; Naughton 2013; Odeny 2014; Perry 2012; Rana 2015; Sloan 2017; Wright 2011
**16**	Participants had preferences regarding the content they receive through digital targeted client communication. They wanted varied content that provided new knowledge and reminders, as well as explanations, solutions, and suggestions about health issues. They were interested in content related to health, illness, and treatments and practical topics such as health facility location and transportation. They wanted this information to be relevant and acceptable to their personal circumstances and local setting.	Moderate confidence	Due to minor concerns regarding methodological limitations and moderate concerns regarding relevance	Brown 2014; Calderón 2017; Cornelius 2009; Entsieh 2015; French 2016; Gold 2010; Greaney 2014; Jalloh‐Vos 2014; Jennings 2013; Mbuagbaw 2014; Missal 2016; Mitchell 2016; Munro 2017; Nachega 2016; Odeny 2014; Perry 2012; Sloan 2017; Smith 2017
**17**	Some participants felt that including elements in the mobile‐based platform in which participants are asked for a response (e.g. via knowledge quizzes or multiple‐choice questions or a practical tool allowing access to additional information, such as a nutrition calculator) could increase the engagement of users with the intervention, its content, and provide additional information to them. In one study, participants suggested that it would be helpful if the response was quick, simple, and convenient.	Low confidence	Due to minor concerns regarding methodological limitations, moderate concerns regarding adequacy, and serious concerns regarding relevance	Cornelius 2009; Munro 2017; Naughton 2013; Wright 2011

**Finding 14: Participants’ perceptions of who sent the digital health communication could influence their trust in and perception of the credibility and value of the DTCC and the information it provides. Participants said they wanted a known, identified phone number; messages sent from a reliable, trusted, credible source such as health professionals or official sources; and in some cases to feel like the messages were sent by a person (even if sent from an automated service). However, some participants, such as those with stigmatised health conditions, preferred an unmarked sender to protect their privacy (moderate confidence).**

Several studies found that participants’ perceptions of who the sender of the message was could influence their trust in and perception of the digital health intervention’s credibility and value (Akinfaderin‐Agarau 2012; Brown 2014; Calderón 2017; Cates 2015; Evans 2016; Greaney 2014; Mbuagbaw 2012; Menacho 2013; Missal 2016; Naughton 2013; Lau 2014; Rana 2015; Rodrigues 2015; Smillie 2014; Willoughby 2017). Participants in many studies identified that the sender should be known and identifiable. If this was not the case many stated that they were more likely to ignore or delete the message (Akinfaderin‐Agarau 2012; Greaney 2014; Smillie 2014), as their phones were already receiving many messages linked with telemarketing (Menacho 2013; Missal 2016). If a sender was unknown, it was also felt that this would decrease the credibility of the message. However, some participants preferred an unmarked sender in order to protect their privacy. This was in the case, for example, of a stigmatised health condition such as HIV, where if the sender's phone number were identifiable, a person’s HIV status may be inadvertently revealed (Rana 2015). These participants felt that a solution would be to allow participants to choose if they wanted the number or name of the sender to be attached to the message (Rana 2015).

Participants also wanted the messages to come from a reliable, trusted, and credible source (Brown 2014; Cates 2015; Evans 2016; Lau 2014; Willoughby 2017). They were interested in the credentials or education of the person creating or responding to the message. Many felt that it was important that the information be written and sent by health professionals and from official sources (Cates 2015; Greaney 2014; Lau 2014; Mbuagbaw 2012). In some cases, if this was unclear, this led them to worry about the accuracy of message content and the intentions of the proposed intervention (Calderón 2017). If the participant knew and trusted the source, they also felt that their data would be protected (Evans 2016).

Many participants felt that it was important that the messages felt like they were coming from an actual person rather than from an automated system ( Lau 2014; Naughton 2013; Rodrigues 2015; Willoughby 2017). Some participants felt that since the messages were written by a health professional, the computer’s role in the automated sending of the messages was irrelevant (Naughton 2013). These participants suggested that messages which were not repeated and were delivered at various times of day using the participant’s name would feel more like they were sent by a person (Naughton 2013).

**Finding 15: Participants said that the tone of digital health communication mattered to them. Their preferences varied but included a tone that was: motivational, friendly, encouraging, polite, respectful, congratulatory, personalised, upbeat, positive, humorous, and relatable. Some participants highlighted that they did not like feeling pressured, lectured, shamed, or frightened by digital health messages (low confidence).**

A number of studies, the majority from HIC contexts (Cates 2015; Curioso 2009; Evans 2016; French 2016; Gold 2010; Jennings 2013; Menacho 2013; Munro 2017; Naughton 2013; Odeny 2014; Perry 2012; Rana 2015; Sloan 2017; Wright 2011), presented data that discussed participants’ preferences related to the tone of the messaging used in DTCC delivered via mobile devices. In general, there was consensus across studies that messages should be polite and respectful. Participants indicated that the tone of the message could influence their acceptance of the message (Cates 2015; French 2016; Jennings 2013); their trust in the message content (French 2016); the credibility of the message; and their engagement with the messages and the digital health intervention (Cates 2015; Curioso 2009; French 2016; Gold 2010; Jennings 2013; Menacho 2013; Munro 2017; Wright 2011).

Participants liked when the tone of the message was, for example, motivational (Curioso 2009; Rana 2015), encouraging (Curioso 2009; Jennings 2013; Munro 2017; Naughton 2013; Odeny 2014; Sloan 2017), upbeat, positive, and reassuring (Evans 2016; Gold 2010; Munro 2017; Perry 2012), friendly (French 2016; Sloan 2017), polite and respectful (Evans 2016; Odeny 2014; Wright 2011), humorous (Gold 2010; Menacho 2013; Wright 2011), or supportive (Jennings 2013; Munro 2017; Odeny 2014; Sloan 2017).

Participants did not like it when messages were used as a warning, to scare people, or were based in fear (Cates 2015; Evans 2016; Munro 2017), or if they felt pressured, told off, shamed, lectured, or patronised by the content (French 2016; Munro 2017).

**Finding 16: Participants had preferences regarding the content they receive through DTCC. They wanted varied content that provided new knowledge and reminders, as well as explanations, solutions, and suggestions about health issues. They were interested in content related to health, illness, and treatments and practical topics such as health facility location and transportation. They wanted this information to be relevant and acceptable to their personal circumstances and local setting (moderate confidence).**

A number of studies presented data related to participants’ preferences regarding the content they received through digital health interventions (Brown 2014; Calderón 2017; Cornelius 2009; Entsieh 2015; French 2016; Gold 2010; Greaney 2014; Jalloh‐Vos 2014; Jennings 2013; Mbuagbaw 2014; Mitchell 2016; Missal 2016; Munro 2017; Nachega 2016; Odeny 2014; Perry 2012; Sloan 2017; Smith 2017). Participants expressed preferences for varied content (Brown 2014; Entsieh 2015; Missal 2016; Perry 2012), that provided them with new knowledge (Brown 2014; Calderón 2017; Cornelius 2009; Entsieh 2015; French 2016; Gold 2010; Jalloh‐Vos 2014; Missal 2016; Munro 2017; Odeny 2014; Perry 2012; Sloan 2017; Smith 2017), or a reminder to take a medication or to reinforce something they knew already (Akinfaderin‐Agarau 2012; Gold 2010; Greaney 2014; Mbuagbaw 2014; Nachega 2016). Participants liked when the content of digital health interventions gave explanations, solutions, and suggestions about health issues (Brown 2014; Entsieh 2015; French 2016; Gold 2010; Greaney 2014; Jalloh‐Vos 2014; Missal 2016; Munro 2017; Perry 2012). An example of such practical advice is the suggestion that mothers who struggled with breastfeeding (or the idea of breastfeeding) could pump their milk and give it to their infants in a bottle instead of switching to formula milk (Brown 2014), as expressed in the quote below from a teenage mother in the USA.

“I was kind of grossed out by actually breast feeding so I had decided to just use formula. I don't know why I was so grossed out, I just was. But then you said in one of the messages that you can pump and feed the breast milk through a bottle, and the baby still gets all those benefits. It makes sense, but I just never thought of it. Because of that message, I started to do that and my baby still gets breast milk, but otherwise I would have given up” (Brown 2014).

Finally, participants were interested in content related to health, illness, and treatments and practical topics such as health facility location and transportation (Brown 2014; Calderón 2017; Greaney 2014; Jalloh‐Vos 2014; Jennings 2013; Missal 2016; Mitchell 2016; Munro 2017; Perry 2012). Many wanted this information to be relevant and acceptable to their personal circumstances and local setting. For example, participants from a few studies expressed an interest in information related to the location of health facilities, including transport information, and contact information for the health facility closest to them to be included in the content (Greaney 2014; Mitchell 2016; Perry 2012).

**Finding 17: Some participants felt that including elements in the mobile‐based platform in which participants are asked for a response (e.g. via knowledge quizzes or multiple‐choice questions or a practical tool allowing access to additional information, such as a nutrition calculator) could increase the engagement of users with the intervention, its content, and provide additional information to them. In one study, participants suggested that it would be helpful if the response was quick, simple, and convenient (low confidence).**

Participants in a few studies, all from HIC contexts (Cornelius 2009; Munro 2017; Naughton 2013; Wright 2011), felt that bi‐directional communication or content that somehow engaged clients (e.g. using quizzes or replies) would be more useful than one‐way communication. Participants mentioned that for this to be the case, the interaction should be made convenient and allow for quick and simple responses. Such interactive options would help keep participants' attention and prolong engagement with the intervention. Participants in one study from the UK, women who had smoked during a previous pregnancy and receive SMS support for smoking cessation, also felt that when the communication asked for a reply they would think more about the content of the messages they were receiving (Naughton 2013), as noted by one participant below.

“An interactive text inviting a reply would make ‘you think more about the text message’ otherwise ‘you don't have to do anything with it so you read it and then forget about it’” (Naughton 2013).

#### Privacy and confidentiality regarding personal health information

('Summary of qualitative findings' table for Findings 18 to 19 is shown in Table 6.)

**6:** 'Summary of qualitative findings' table for findings related to privacy and confidentiality regarding personal health information

**Finding**		**Overall CERQual assessment**	**Explanation for assessment**	**Contributing studies**
**18**	Some participants with health issues that are often seen as stigmatised or very personal (e.g. HIV, family planning, and abortion care) worried that their confidential health information would be disclosed or their identity traced due to their participation in digital targeted client communication. In general, people’s perceptions of information delivery channels (SMS, interactive voice response, voice call) were influenced by how confidential they felt the delivery channels to be.	High confidence	Due to minor concerns regarding methodological limitations	Akinfaderin‐Agarau 2012; Calderón 2017; Cates 2015; Curioso 2009; Evans 2016; French 2016; Goldenberg 2015; Greaney 2014; Jalloh‐Vos 2014; Jennings 2013; Mbuagbaw 2012; Mbuagbaw 2014; Menacho 2013; Mitchell 2016; Nachega 2016; Odeny 2014; Perry 2012; Rana 2015; Rodrigues 2015; Smith 2017; Willoughby 2017
**19**	Some participants proposed strategies to address their concerns regarding confidentiality and privacy. These strategies for communication included neutral, coded, or discreet language; access codes; communication that does not disclose the sender; coming from a trusted sender; and the ability to tailor and control content, timing, and frequency of their messages.	High confidence	Due to minor concerns regarding methodological limitations	Calderón 2017; Curioso 2009; Evans 2016; French 2016; Goldenberg 2015; Greaney 2014; Mbuagbaw 2012; Menacho 2013; Odeny 2014; Rana 2015; Rodrigues 2015; Smith 2017; Willoughby 2017

Below we present findings specifically related to privacy and confidentiality. However, this theme is also touched upon in a number of other findings including Finding 7.

**Finding 18: Some participants with health issues that are often seen as stigmatised or very personal (e.g. HIV, family planning, and abortion care) worried that their confidential health information would be disclosed or their identity traced due to their participation in DTCC. In general, people’s perceptions of information delivery channels (SMS, interactive voice response, voice call) were influenced by how confidential they felt the delivery channels to be (high confidence).**

A number of studies, the majority from LMIC settings (Akinfaderin‐Agarau 2012; Calderón 2017; Cates 2015; Curioso 2009; Evans 2016; French 2016; Goldenberg 2015; Greaney 2014; Jalloh‐Vos 2014; Jennings 2013; Mbuagbaw 2012; Mbuagbaw 2014; Menacho 2013; Mitchell 2016; Nachega 2016; Odeny 2014; Perry 2012; Rana 2015; Rodrigues 2015; Smith 2017; Willoughby 2017), found that some participants had concerns about the extent to which their privacy and personal information were sufficiently protected. This was especially true for those who were dealing with health conditions that are often seen as stigmatised or very personal. People participating in interventions related to HIV and AIDS expressed the strongest concerns, especially for interventions that sent HIV testing reminders or reminders to take medication (Akinfaderin‐Agarau 2012; Curioso 2009; Evans 2016; Goldenberg 2015; Jennings 2013; Mbuagbaw 2012; Mbuagbaw 2014; Menacho 2013; Nachega 2016; Odeny 2014; Rana 2015; Rodrigues 2015). These participants worried that the SMS or phone conversation would reveal their status to people who picked up their phones or who overheard their conversations. However, in one study from Kenya, participants felt that receiving an SMS or phone call would protect their privacy more than a face‐to‐face appointment, as conversations in a health facility were easily overheard by others. Some participants in a family planning digital communication study in Sierra Leone feared that participating in the intervention would compromise their privacy because their husbands or family members would find out they were using family planning methods (Jalloh‐Vos 2014). Similarly, a participant in a postabortion care intervention in Cambodia was worried that her medical history of abortion would be discovered by her family if she was to receive a phone call in their presence or if someone else were to answer her phone (Smith 2017). Some adolescents participating in interventions related to sexual and reproductive health felt that if others saw the messages it might be embarrassing; it may cause their parents to ask them or they may be suspected of having a disease (Akinfaderin‐Agarau 2012; Perry 2012; Willoughby 2017). Some participants had more general worries that their private information, such as banking details and personal health information, would be disclosed or shared (Calderón 2017; Goldenberg 2015; Mitchell 2016).

Participants in a few studies expressed opinions on how confidential they felt different delivery channels were. Some believed that texting provided more privacy than receiving a voice call (Cates 2015; Curioso 2009; French 2016; Menacho 2013; Perry 2012). Some participants felt that text messages were easier, more confidential, and more readily available (Curioso 2009). Others felt that they had more control over text messaging as they could prevent the messages from appearing on their phone screens, could lock their phones, and could delete messages (French 2016).

**Finding 19: Some participants proposed strategies to address their concerns regarding confidentiality and privacy. These strategies for communication included neutral, coded, or discreet language; access codes; communication that does not disclose the sender; coming from a trusted sender; and the ability to tailor and control content, timing, and frequency of their messages (high confidence).**

In some studies, many from LMIC settings (Calderón 2017; Curioso 2009; Evans 2016; French 2016; Greaney 2014; Goldenberg 2015; Mbuagbaw 2012; Menacho 2013; Odeny 2014; Rana 2015; Rodrigues 2015; Smith 2017; Willoughby 2017), participants presented strategies that could be implemented to address their concerns regarding privacy and confidentiality. Some participants felt that the best way to protect their privacy would be to use neutral or coded language (Curioso 2009; Goldenberg 2015; Mbuagbaw 2012; Menacho 2013; Odeny 2014; Rana 2015; Willoughby 2017). This was especially true for those receiving messages about sensitive topics such as HIV and family planning. Others felt that privacy could be protected by not disclosing the sender of the message and making sure the message was sent from a trusted source (Evans 2016; Greaney 2014). Some participants suggested that digital health interventions use access codes or passwords that participants would have to enter to gain access to messages (Calderón 2017; French 2016; Goldenberg 2015; Rana 2015). Finally, many participants believed that some of their concerns about privacy could be addressed by allowing them to tailor and control various aspects of the interventions such as the content, frequency, and timing of their messages (Goldenberg 2015; Rodrigues 2015; Smith 2017).

#### Perceptions of intervention impact

('Summary of qualitative findings' table for Findings 20 to 25 is shown in Table 7.)

**7:** 'Summary of qualitative findings' table for findings related to perceptions of programme impact

**Finding**		**Overall CERQual assessment**	**Explanation for assessment**	**Contributing studies**
**20**	Some participants thought that participating in digital targeted client communication had influenced their behaviour, whilst others did not. Reasons given for the changes in behaviour included receiving new knowledge; receiving strategies on how to initiate discussion with a partner or healthcare provider; being motivated or reassured by the intervention; and being reminded, for example, to take medication or make an appointment. Some participants who believed that the intervention did not have any influence on their behaviour found that the digital health interventions were not relevant to them.	Low confidence	Due to minor concerns regarding relevance and adequacy and moderate concerns regarding methodological limitations	Brown 2014; Entsieh 2015; French 2016; Gold 2010; Greaney 2014; Hirsch‐Moverman 2017; Jalloh‐Vos 2014; Jennings 2013; Lau 2014; Missal 2016; Munro 2017; Rodrigues 2015; Sloan 2017; Smillie 2014; Smith 2017; Ware 2016
**21**	Some participants suggested that the effects of the messaging may not be sustained over time, as they and others would become bored with or fatigued by the messages, especially if the content was not varied enough.	Low confidence	Due to moderate concerns regarding relevance and adequacy	Cornelius 2009; Curioso 2009; Evans 2016; Gold 2010; Menacho 2013; Mitchell 2016; Rana 2015; Willoughby 2017
**22**	Some participants were concerned about becoming over‐reliant on digital reminders and thought that this might make them dependent on digital targeted communication for undertaking some health tasks. They were concerned that in the absence of these reminders they would adhere poorly to care plans.	Low confidence	Due to minor concerns regarding methodological limitations, moderate concerns about relevance, and serious concerns about adequacy	Jalloh‐Vos 2014; Mbuagbaw 2012; Rana 2015
**23**	Some participants felt that digital health interventions could save them time and money by giving them access to health care via their mobile phones. This was especially relevant to participants who faced barriers in attending health care because of distance to a health facility and a lack of time and or financial means.	Low confidence	Due to minor concerns regarding methodological limitations and moderate concerns regarding adequacy and relevance	Calderón 2017; Smith 2017
**24**	Some participants felt that digital health interventions provided them with feelings of support and connectedness, as they felt that someone was taking the time to send them messages. A few participants felt that in some cases the sense of caring and support that they received from healthcare providers through digital health interventions had a positive influence on their relationship with their healthcare provider.	Moderate confidence	Due to moderate concerns regarding methodological limitations and relevance	Brown 2014; Calderón 2017; Entsieh 2015; Jalloh‐Vos 2014; Lau 2014; Mbuagbaw 2014; Munro 2017; Nachega 2016; Rana 2015; Rodrigues 2015; Sloan 2017; Smillie 2014; Smith 2017; Ware 2016; Wright 2011
**25**	Participants described how they shared digital communication content more broadly with friends, family, and community members. Many participants felt that the information would be useful to others.	Moderate confidence	Due to minor concerns regarding methodological limitations and moderate concerns regarding relevance	Calderón 2017; Cornelius 2009; Flax 2017; French 2016; Gold 2010; Jennings 2013; Perry 2012; Smith 2017; Wright 2011

**Finding 20: Some participants thought that participating in DTCC had influenced their behaviour, while others did not. Reasons given for the changes in behaviour included receiving new knowledge; receiving strategies on how to initiate discussion with a partner or healthcare provider; being motivated or reassured by the intervention; and being reminded, for example, to take medication or make an appointment. Some participants who believed that the intervention did not have any influence on their behaviour found that the digital health interventions were not relevant to them (low confidence).**

A number of studies found that participants thought that taking part in DTCC had influenced their behaviour (Brown 2014; Entsieh 2015; French 2016; Gold 2010; Greaney 2014; Hirsch‐Moverman 2017; Jalloh‐Vos 2014; Jennings 2013; Lau 2014; Missal 2016; Munro 2017; Rodrigues 2015; Sloan 2017; Smillie 2014; Smith 2017; Ware 2016), while others felt that it had no impact.

In some studies participants felt that the targeted client communication had had a positive impact on their behaviour. New knowledge about child and maternal health such as vaccinations and breastfeeding and antenatal care, Brown 2014; Entsieh 2015, risk of sexually transmitted infections (Gold 2010), the husband’s role and how he can support his wife during pregnancy and delivery (Missal 2016), and family planning methods (Smith 2017), had influenced participant actions, including for testing or choosing a particular contraceptive method.

Digital targeted client communication also provided some participants with specific strategies, for example how to discuss a health topic with a significant other. Some studies found that this led to participants talking to their partners about testing for sexually transmitted diseases (French 2016; Jennings 2013); preparing for labour and delivery (Missal 2016); or discussing questions with a healthcare provider (Munro 2017). Participants felt that without these strategies they might not have taken the steps to have these conversations about their health.

Messages also motivated or reassured participants about their health decisions. Some participants felt a sense of confidence in their parenting decisions, as the messages they received validated their parenting choices (Brown 2014); or made them feel like the difficult conversations about disease testing were the right thing to do (French 2016; Jennings 2013); or motivated them to quit smoking (Sloan 2017), or take their medications on time (Smillie 2014). Messages also made participants feel less apprehensive about procedures such as sexually transmitted infection testing (Gold 2010), or continuing contraceptive use after side effects occurred (Smith 2017). In other cases the messages motivated women to visit the health facility more frequently (Jalloh‐Vos 2014), or husbands to support their wives during pregnancy (Missal 2016).

Some participants felt that the reminders delivered through digital health interventions helped them remember to take their medication (Hirsch‐Moverman 2017; Smith 2017; Rodrigues 2015; Ware 2016); get tested (French 2016; Jennings 2013); improve their use of condoms (French 2016); adhere to or engage in treatment (Jennings 2013; Lau 2014); schedule or attend appointments (Greaney 2014; Jalloh‐Vos 2014; Smillie 2014); or by just keeping sexual health “up in their mind” (Gold 2010).

In some studies (Gold 2010; Greaney 2014; Hirsch‐Moverman 2017; Rodrigues 2015; Smillie 2014,), participants believed that the intervention had no impact on their behaviour, whether it be remembering to take medications (Hirsch‐Moverman 2017; Rodrigues 2015; Smillie 2014); testing for sexually transmitted infections and using condoms (Gold 2010); or scheduling an appointment for screening (Greaney 2014).

**Finding 21: Some participants suggested that the effects of the messaging may not be sustained over time, as they and others would become bored with or fatigued by the messages, especially if the content was not varied enough (low confidence).**

Some studies found that some participants thought the effects of digital health messaging may not be sustainable, as they or other users might become fatigued by the messages (Curioso 2009; Cornelius 2009; Evans 2016; Gold 2010; Menacho 2013; Mitchell 2016; Rana 2015; Willoughby 2017). Participants thought that this message fatigue would most likely occur if the frequency, content, and topics were not varied enough (Evans 2016; Gold 2010; Menacho 2013).

**Finding 22: Some participants were concerned about becoming over‐reliant on digital reminders and thought that this might make them dependent on digital targeted communication for undertaking some health tasks. They were concerned that, in the absence of these reminders, they would adhere poorly to care plans (low confidence).**

A few studies, from LMIC settings in Africa (Jalloh‐Vos 2014; Mbuagbaw 2012; Rana 2015), found that some participants were concerned about over‐reliance on digital reminders, and that in their absence adherence might become problematic. Two studies explored perceptions related to adherence to HIV medication, and another family planning and antenatal/postpartum care. These concerns were important to participants that mentioned the problem of reliance, as they believed that the digital health interventions would eventually end and people needed to remember themselves (Rana 2015). For example, in Sierra Leone, both women and men mentioned that if the nurse did not call to remind them about family planning they would forget, and that it was the nurse's job to remind them to come (Jalloh‐Vos 2014).

**Finding 23: Some participants felt that digital health interventions could save them time and money by giving them access to health care via their mobile phones. This was especially relevant to participants who faced barriers in attending health care because of distance to a health facility and a lack of time and or financial means (low confidence).**

Two studies from LMIC settings found that participants believed that digital health interventions could potentially save them time and money by giving them access to health care through their mobile phones instead of having to go to the health facility (Calderón 2017; Smith 2017). In Peru (Calderón 2017), women over 18 who had at least one child believed that this would be useful to families living in resource‐poor communities, where families often cannot afford the cost or time related to transport to the health facility or the consultation. They felt that the intervention would be used more by families that live further away from a health facility, described as follows.

“People like me who work all day could use it, we have to use it, because they then already have someone to ask, you will not be with that doubt ‘do I give or not give the medication to the baby?’ or ‘how much medication do I have to give him/her?’ I don’t know, so when we have someone to call or to send text messages to, you will not hesitate to do it as they say” (Calderón 2017).

Women in Cambodia also felt that the digital health intervention saved them time and money, as they could receive their family planning counselling over the phone instead of having to travel to the health facility. This saved them both the fees associated with transport and the consultation with the healthcare provider (Smith 2017).

**Finding 24: Some participants felt that digital health interventions provided them with feelings of support and connectedness, as they felt that someone was taking the time to send them messages. A few participants felt that in some cases the sense of caring and support that they received from healthcare providers through digital health interventions had a positive influence on their relationship with their healthcare provider (moderate confidence).**

A number of studies, the majority from LMIC settings (Brown 2014; Calderón 2017; Entsieh 2015; Jalloh‐Vos 2014; Lau 2014; Mbuagbaw 2014; Munro 2017; Nachega 2016; Rana 2015; Rodrigues 2015; Sloan 2017; Smillie 2014; Smith 2017; Ware 2016; Wright 2011), presented data related to participants’ reflections around the caring and supportive nature of DTCC. In some cases, the participants in these studies felt that someone was interested in their situation, was invested in their well‐being, and cared about them. This led some participants to feel encouraged and to have increased self‐confidence and feelings of self‐worth. For others, the messages provided support, guidance, and information, often giving a sense of direction, reassurance, and motivation to participants. Support was presented in various ways, for example to take medications or providing counselling. In some cases, this increased dialogue between healthcare workers and participants had a positive influence on their relationship, for example by making each group more aware of the other’s expectations (Jalloh‐Vos 2014; Smillie 2014; Smith 2017).

**Finding 25: Participants described how they shared digital communication content more broadly with friends, family, and community members. Many participants felt that the information would be useful to others (moderate confidence).**

Some studies found that participants shared messages they had received or thought they would share messages with friends, family, colleagues, or neighbours (Calderón 2017; Cornelius 2009; Flax 2017; French 2016; Jennings 2013; Perry 2012; Smith 2017; Wright 2011). Many participants felt that it was important to share this information, as they had found the messages helpful and believed the information would be useful to others (Calderón 2017; Flax 2017; French 2016; Gold 2010; Jennings 2013; Perry 2012; Smith 2017).

Participants in one study felt that sharing messages and experiences could help to create a sense of community (Wright 2011). In another study, participants were excited about the possibility of creating message content to share with friends and family (Cornelius 2009).

Finally, one study sent SMSes to pre‐existing community‐based women’s groups about maternal and child health topics. Not all of the women in the group were pregnant or had children. However, these participants mentioned that they still found the content interesting and discussed it with other group members, family, and neighbours (Flax 2017).

### Supplementing the Cochrane Reviews of effectiveness with synthesised qualitative findings – matrix results

As described in the Methods section, we used a matrix approach to explore whether potential implementation barriers that we had identified in our synthesis (see Table 8 below) had been addressed in the programmes evaluated in the related Cochrane Reviews of effectiveness (Palmer Ongoing a; Palmer Ongoing).

The relevance of our synthesis findings to the reviews of effectiveness was strengthened by the fact that the studies included in the qualitative synthesis and those included in the effectiveness reviews came from similar settings. Around half of the studies in both the synthesis and the reviews were from low‐income countries (primarily African countries), whilst the remaining studies where from high‐income countries (primarily the USA). In addition, around 10 of the qualitative studies included in our synthesis appear to have been carried out to inform the development of some of the trials.

Table 8 presents an overview of our matrix assessment (a more detailed version can be found in Appendix 5, and a list of all of the studies included in the analysis is shown in Appendix 6). In summary, we found that some of the included trials did describe efforts to address some of the potential barriers to implementation that we had identified in the qualitative synthesis. However, most of the trials referred to only a small number of barriers, and in some trials potential participants were actively excluded if they were dealing with these barriers.

One barrier identified in our synthesis was related to situations where the target group did not own a functioning mobile phone. In more than half of the trials, trialists dealt with this barrier by making mobile phone ownership a condition for trial participation. Between 0.3% and 63% of eligible participants were excluded from trial participation because they did not own a phone or could not receive text messages. Some trialists *did* attempt to find solutions to this problem, however. For instance, some trialists only required that participants had *access* to a phone, for instance through family, friends, or neighbours (Brown 2016; Gibson 2017; Kassaye 2016; Odeny 2014); in other trials participants were provided with phones (e.g. Cook 2015; Ingersoll 2015); and in one trial, local healthcare providers were given phones to share with women who had no access to phones (Joshi 2015).

Other barriers identified in our synthesis were tied to poor access to electricity to charge phones and poor access to network services. However, trialists rarely referred to these issues. If mentioned, this was usually because access to specific networks or to stable electricity was a condition for participation in the trial. Trials that did attempt to deal with these barriers included one trial based in the USA (Ingersoll 2015). Here, assessments carried out prior to the trial showed that many people had inconsistent cellular or internet service, but that they could usually receive text messages. The trialists therefore built a texting system rather than one that would require a consistent cellular signal or internet access (Ingersoll 2015). Another exception was a Kenya‐based trial (Pop‐Eleches 2011). As many participants in this trial needed to charge their phones at fee‐based charging stations in nearby markets, the trialists provided financial support to cover these fees (Pop‐Eleches 2011).

Another barrier was tied to the expenses associated with the trial interventions. This was also confirmed as a problem by some trialists, who described poor participation because of cost barriers (Ahlers‐Schmidt 2012; Lester 2010; Norton 2014). Around one‐third of the trials described efforts to address this problem in some way, for instance by offering vouchers to participants (e.g. Lund 2012; Omole 2018), or by offering participants free access to various internet sites in return for receiving mobile advertising (Gold 2011).

Our synthesis also pointed to problems in receiving messages when people changed their phone numbers or sim cards. Again, this issue was rarely referred to in the trials. When mentioned, this was usually as an explanation for participant loss to follow‐up or as a condition for trial participation. Examples of trials that *did* try to address this issue include a trial from India, where participants with new phones were able to update their contact details with their local health worker (Joshi 2015); a USA‐based trial (Bull 2016), where participants were asked to update information about their phone number every month (Devine 2014); and a trial from Ecuador, where trialists gathered several different contact avenues to maximise their ability to contact participants (Maslowsky 2016).

Two related barriers were tied to situations where people had to share phones or had their phones controlled by others, and situations where these people, as well as people with their own phones, were concerned about privacy issues when receiving messages. Very few trialists discussed situations whereby people’s phones might be controlled by others, and at least two‐thirds did not refer to privacy issues. However, several trialists did describe efforts to address issues of privacy and confidentiality. These included trials targeting people about oral contraception (Castano 2012), or HIV treatment and prevention (Garofalo 2016; Mbuagbaw 2012; Ybarra 2016) where trialists ensured that messages did not refer to the recipient’s name, their health status, or the name of the medication; encouraged recipients to delete messages; and gave them information about privacy settings on their phones.

Other barriers identified in our synthesis were created by language problems, low literacy, or limited techno‐literacy. At least a third of the trials made no reference to these issues. Another third actively excluded people who did not speak one or two mainstream languages, and several trials also excluded people who could not read these languages or who were illiterate. Some trialists described efforts to overcome these problems, including designing text messages so that they scored low on readability scales ( Bigna 2014; Stockwell 2014); offering text messages in the local language (Kassaye 2016); or sending voice messages or pictorial messages instead of text messages (de Costa 2012; Smith 2015).

Our synthesis also found that people’s trust in and perceptions of the information they received was influenced by their perceptions of the sender. Attempts to address this issue were rarely described by trialists. Exceptions included a UK‐based trial, Cooper 2015, that highlighted in advertising materials that the intervention was supported by the National Health Service (NHS) (Emery 2018); and a USA‐based trial (Bull 2016), where messages were linked to celebrities that the target audience said were most interesting to them (Devine 2014).

The final issue we assessed in our matrix was the extent to which the target group had been given an opportunity to offer feedback about their needs, preferences, and experiences regarding the intervention. More than half of the trialists described collecting user feedback to develop or improve the intervention, although it was not always clear how information gathered before, during, or after the trial had influenced the intervention or would influence future versions of the intervention.

## Discussion

### Summary of main results

The acceptability of digital targeted client communication (DTCC) is mixed**.** Some clients described DTCC in positive terms, as providing them with support and connectedness; giving them a feeling that someone cares about them; and, in some cases, having a positive influence on their relationship with their healthcare provider. However, clients who are dealing with health conditions that are often stigmatised or very personal (e.g. HIV, family planning, and abortion care) worry that their confidential health information will be disclosed or their identity traced.

Clients' perceptions and experiences of DTCC can be influenced by a number of factors. Participants believed that there should be little or no charge to participate in digital health interventions. They wanted messages that were from a trusted sender and that were polite and encouraging. They did not like feeling pressured, lectured to, or frightened. They wanted varied information that arrived at a time and frequency that was convenient for them. Content preferences included new knowledge, reminders, solutions, and suggestions about health issues presented in a clear, short, and personalised way.

In general, barriers to participating in digital health interventions included problems with network connectivity, access to electricity, device usability, and issues tied to data confidentiality and privacy.

Some of our findings addressed issues related to gender, equity, and human rights. For example, access to healthcare services via digital devices may be particularly helpful to clients with caring or work responsibilities; clients who live far from health facilities; and poorer people. However, access to digital health interventions may be particularly difficult for others who speak minority languages or who have low literacy skills or low digital literacy skills, who for example do not know how to open, read, or send a text message. Participation may also be difficult for clients with poor access to network services, electricity, or ownership of mobile phones. For clients, particularly women and adolescents, who have to share or borrow a phone or who have phone access controlled by others, it can be difficult to receive messages or keep them private. Clients with stigmatised health conditions such as HIV were also concerned about what would happen if their privacy was not protected, for example if the fact that they were participating in the digital health intervention led to the disclosure of their HIV status.

### Overall completeness and applicability of evidence

The sampling approach we used in this review (see above) aimed to achieve a maximum variation of target populations, settings, delivery mechanisms, and content focus of the targeted digital health intervention.

We found studies that represented clients from all of the client groups included in the scope of this synthesis. However, studies from parents and pregnant and postpartum women and their partners were not as common. For example, we found very few studies researching DTCC for HIV‐positive pregnant and postpartum women. We are therefore less certain whether these client groups have the same perspectives and expectations of targeted digital health interventions.

A majority of the studies covered topics related to sexual and reproductive health for young people and adults, with a focus on sexually transmitted infections and specifically HIV/AIDS. We are therefore less certain about whether participants receiving digital health interventions related to other topics have the same preferences for information and intervention delivery, such as vaccination and general child health.

The studies included in this review come from a wide variety of contexts and settings. There are a number of included studies from poorly resourced healthcare systems as well as high‐income settings. The range of settings included in this review highlighted access issues as well as issues related to gender and equity across all settings.

The majority of the studies included in this synthesis looked at digital health interventions where the content was or would be delivered by SMS. We have only a few studies that explore perceptions related to other delivery methods such as interactive voice response (IVR).

The data collection methods and study designs of the included studies may in some cases limit the applicability and completeness of the data reported. One example of this is the large number of studies that presented hypothetical digital health interventions (18 studies), both with and without examples of content, and asked participants to reflect on their preferences if they would participate. It is unclear whether these preferences based on hypothetical thinking would remain the same once the participant had actually experienced participating in the intervention and receiving the messages.

All of the included studies made use of individual or group interviews and focus group discussions as their main method of data collection. None used long‐term ethnographic methods or field observation. While interviews and focus groups allow researchers to collect data on what people say, observational methods also allow researchers to collect data on what people do and why. This would have been appropriate for understanding how clients engaged with and used the various digital health interventions and allowed researchers to compare actions with interview and focus group data. Interviews and focus group discussions seem to be the most commonly used research methods amongst qualitative researchers exploring issues related to health. This could be because they are less time‐consuming than longer‐term ethnographic methods.

### Agreements and disagreements with other studies or reviews

The review team identified one published qualitative evidence synthesis that had a similar scope to this review (O’Connor 2016). The O'Connor synthesis had a much broader scope and explored factors affecting client engagement and recruitment to digital health interventions. Also, these interventions did not have to be targeted at a specific audience and were open to any health intervention delivered by a digital technology. We are aware of an ongoing related qualitative evidence synthesis considering healthcare workers’ experiences with and perceptions of targeted digital communication via mobile device (Odendaal 2015). A number of reviews of the effect of mHealth programs in general have been carried out (Anglada‐Martinez 2015; Ahmed 2017; Aranda‐Jan 2014; Catalani 2013; Cole‐Lewis 2010; Free 2013; Free 2016; Gurman 2012; Krishna 2009; Lee 2016; Peiris 2014). Two overviews of systematic reviews looking at text messaging (Hall 2015), and the impact of mHealth interventions (Marcolino 2018), have also been done. However, many of these reviews and overviews address health issues beyond the scope of our synthesis, such as obesity and chronic illness (Bacigalupo 2013; Hamine 2015; Peiris 2014).

In 2015, a review was published that explored the adoption of mHealth in low resource environments (Chib 2015). This review found that the majority of studies in these environments concentrated principally on pilot projects focused mostly on the introduction and implementation of new interventions. This finding is similar to the types of studies we found in this synthesis, where approximately half of the included studies were projects using hypothetical examples to develop targeted digital health interventions. A further six studies were pilot projects for new targeted digital health interventions. We only identified one study that explored client experiences related to an ongoing project at scale, Mobile Midwife in Ghana (Entsieh 2015). Other systematic reviews have identified a similar large number of pilot studies (Catalani 2013; Gurman 2012). In the absence of studies of large‐scale implementation (where the targeted digital communication has become a routine part of care), it is difficult to say if people’s experiences and perceptions of smaller studies are transferable to interventions delivered at scale.

Our qualitative evidence synthesis highlighted issues of access to mobile phone technology, networks, and electricity. Other studies have highlighted this issue as well (Aranda‐Jan 2014; Bukachi 2007). As discussed in the findings of our matrix analysis, future trials should take these access issues into consideration when defining participant inclusion criteria. At this time, little is known about how targeted digital health communication interventions are used or perceived by those who do not have access to phones, networks, or electricity, as the large majority of existing trials and qualitative studies include participants who have phones and access to networks and electricity. Our findings highlight a potential lack of digital literacy among clients in many settings, which could also affect the feasibility and acceptance of targeted digital health communication interventions. Similarly, the O’Connor 2016 review concluded that "more investment is also needed to improve computer literacy and ensure technologies are accessible and affordable for those who wish to sign up to them”.

Finally, issues related to tailoring and personalisation of timing, format, content, and privacy were clearly described in the findings of this review. One other review, Gurman 2012, mentions that less than half of the interventions included described targeting or tailoring the content. A meta‐analysis of tailored print health behaviour change interventions found that tailored interventions were more effective than non‐tailored interventions for health promotion (Noar 2007). The O’Connor 2016 review also found that interventions that are personalised when possible should be a focus when creating digital health interventions. To support this thinking, research has found that successful intervention design demands a user‐centred and iterative approach to developing new digital behaviour change interventions (Yardley 2016). Tailoring was also identified as a core interactive design feature in effective e‐health interventions (Morrison 2012).

### Summary of integrating the findings from this synthesis with the findings of relevant Cochrane effectiveness reviews

We used a matrix approach to explore how the findings from our synthesis related to, or could help to inform, the findings of the two related Cochrane Reviews of effectiveness of DTCC (Palmer Ongoing a; Palmer Ongoing).

Our synthesis and the two intervention reviews were designed to complement each other, and used similar inclusion criteria where possible. The included studies in the synthesis and the two reviews were therefore broadly similar in terms of population groups, delivery mechanism, setting, and publication date. All three syntheses/reviews included studies with the same population groups (i.e. adolescents and adults that are users or potential users of reproductive health services; pregnant and postpartum women, including women living with HIV; and parents of children under five). In all three syntheses/reviews, the majority of programmes used text messages to communicate with their target audiences. Slightly more than half of the 35 studies in the synthesis were from LMICs. Slightly more than half of the 68 studies in the intervention reviews were also from LMICs. The studies sampled in our synthesis were published between 2009 and 2017, whilst the trials included in the intervention reviews were published between 2006 and 2018.

Our matrix shows that most of the potential barriers to implementation raised by participants in the qualitative research were not referred to in most of the trials. It is possible that trialists did attempt to find solutions but did not report these in their publications. It is also possible that our search strategy failed to identify all relevant publications, and that these solutions were reported elsewhere. For instance, many of the trialists did not describe how they dealt with privacy issues when participants received messages in the papers we examined. However, it is possible that some privacy issues were addressed to some extent during scientific ethics review and approval processes.

Some of the included trials *did* describe efforts to address some of these issues. Where this occurred, it would be useful to explore the impact, transferability, and potential sustainability of these efforts. Some of the solutions described by trialists are likely to be sustainable, including efforts to increase accessibility of messages through audio, pictures, and local languages; or protecting people’s privacy though anonymising messages. However, other solutions may be less sustainable outside of a trial context or may be unacceptable for other reasons. These include the distribution of free phones or covering the costs of recharging phones. Other solutions, such as sharing of phones, may also lead to concerns about privacy.

In several trials, trialists simply excluded participants who were dealing with the barriers we identified in our synthesis. The exclusion of participants who do not own their own phones; who are likely to change their phone number or sim card; or because of language, literacy, or techno‐literacy issues is problematic. Whilst the size of these challenges varies from setting to setting, people that experience these challenges may be the same people who need health services the most, but who access them the least. Interventions that specifically aim to increase people’s access to and use of healthcare services should therefore make a particular effort to address these challenges.

Table 8 below presents an overview of the findings of our matrix analysis. The full table can be found in Appendix 5. The table starts with 10 questions identified from the qualitative synthesis findings. The table then indicates how many studies from each of the client groups addressed the question (Y), or not (N); if the description was unclear (?) or if the topic of the question was not mentioned in the intervention study (NM). The table ends by displaying the total number of studies in numbers and percentages that addressed the question (Y), or not (N); if the description was unclear (?) or if the topic of the question was not mentioned in the intervention study (NM).

#### Table 8: Integrating findings from this synthesis with the findings of relevant Cochrane effectiveness reviews

**Table:** 

Have the trialists described efforts to address situations where members of the target group: 1. do not own a functioning mobile device; 2. have poor access to network services; 3. have poor access to electricity to charge mobile devices; 4. want to avoid expenses associated with the intervention, such as paying for airtime; 5. change their phone numbers or sim cards; 6. have access to the phone controlled by someone else; 7. have low literacy, differing language skills, or limited techno‐literacy; 8. have concerns about privacy and confidentiality; 9. perceive different sources as more or less reliable, trusted, and credible; 10. have members of the target group been given an opportunity to offer feedback about their needs, preferences, and experiences regarding the intervention.										
	**1**	**2**	**3**	**4**	**5**	**6**	**7**	**8**	**9**	**10**
**Adolescents (N = 13)**										
**Y**	3	1	1	5	2	1	2	6	1	7
**N**	9			1	3	1	5			1
**?**				1	2	1	2			3
**NM**	1	12	12	6	6	10	4	7	12	2
**Adults (N = 27)**										
**Y**	7	2	1	12		2	8	10	1	17
**N**	15	2	1	3	7	1	5	1		
**?**		1					5	2	1	1
**NM**	5	22	25	12	20	24	12	14	25	9
**Pregnant and postpartum women (N = 11)**										
**Y**	2	1		4	3		2		1	5
**N**	7		1				4			1
**?**							3			2
**NM**	2	10	10	7	8	11	2	11	10	3
**Parents (N = 14)**										
**Y**	2						5			7
**N**	10				3		3			
**?**					1		1	1		
**NM**	2	14	14	14	10	14	5	13	14	7
**Mothers living with HIV (N = 3)**										
**Y**	2			2			2	1		2
**N**										
**?**						1		1		
**NM**	1	3	3	1	3	2	1	1	3	1
**Total for all population groups (N = 68)**										
**Y**	16 (24%)	4 (5%)	2 (3%)	23 (35%)	5 (7%)	3 (4%)	19 (27%)	17 (25%)	3 (4%)	38 (57%)
**N**	41 (60%)	2 (3%)	2 (3%)	4 (5%)	13 (19%)	2 (3%)	17 (25%)	1 (1%)		2 (3%)
**?**		1 (1%)		1 (1%)	3 (4%)	2 (3%)	11 (15%)	4 (5%)	1 (1%)	6 (8%)
**NM**	11 (16%)	61 (91%)	64 (94%)	40 (59%)	47 (70%)	61 (90%)	24 (34%)	46 (69%)	64 (95%)	22 (32%)
N = No; Y = Yes; ? = Unclear; NM = Not mentioned										

### Review author reflexivity

As part of the synthesis process, we reflected on how our backgrounds and positions might have influenced our choice of review topic, study selection, data extraction, analysis, and interpretation of data. Our backgrounds are in health systems research, social sciences, epidemiology, and nursing, and while working on the synthesis we were employed by government research institutions in Norway, South Africa and the USA (HA, CG, SL, NL, EA) and by the WHO (TT). The synthesis was commissioned to inform a WHO guideline, specifically to address guideline questions regarding the acceptability and feasibility of digital health interventions. Three of us were key members of the WHO guideline technical team (TT, SL, CG). Some of us had been involved in primary research related to digital health interventions, whilst others (CG, HA) had no previous work experience on this topic. Before working on the synthesis, our viewpoints regarding digital health interventions ranged from being neutral to these types of interventions to a slight skepticism of digital interventions as a magic bullet in solving health issues. All of us started the process believing that the implementation of digital health interventions should be informed by robust evidence of effectiveness, acceptability, and feasibility. Whilst working on the synthesis, we became more convinced of the importance of supporting evidence‐based decision‐making in digital health ‐ especially having seen from the studies included in this review, as well as from several other reviews commissioned for the WHO guideline, the range of challenges and constraints in implementing these interventions at scale, and in ways that protect the privacy of participants.

## Authors' conclusions

### Implications for practice

The following questions, derived from our findings, may help health system or programme managers when implementing or planning for digital targeted client communication strategies to address issues of importance to their target population. It is important to consider local contextual factors including gender, age, cultural group, and education when implementing new digital targeted client communication strategies.

1. Do clients own or have access to a functioning mobile device? If not, have solutions to access issues been considered?
2. Do clients have access to network services in the area where they will be engaging with the digital health intervention? If not, have solutions to access issues been considered?
3. Do clients have access to electricity to charge mobile devices? If not, have solutions to access issues been considered?
4. Is participating in the digital health intervention free or of very limited cost to ensure that there are no barriers to participation? If not, have solutions to access issues been considered?
5. Have solutions been considered for when clients change their phone numbers or sim cards in order to maintain intervention engagement and avoid losing contact?
6. Have solutions been considered for when clients have their access to a phone controlled by someone else?
7. Have solutions been considered for tailoring or changing intervention content to engage clients who have low literacy, differing language skills, or limited digital literacy?
8. Have solutions been considered for tailoring or changing intervention content to ensure the privacy and confidentiality of clients and to avoid any harms that a break in this privacy may cause?
9. Has an attempt been made to explore how clients perceive different sources of digital health interventions as more or less reliable, trusted, and credible? Has an attempt been made to use those sources that are perceived as trusted, reliable, and credible to send digital health messages?
10. Have members of the client target group been given an opportunity to offer feedback about their needs, preferences, and experiences regarding the intervention during intervention development, implementation, and evaluation?

### Implications for research

These implications have been derived from the CERQual assessment and the overview of the studies included in this review.

There is a need for better reporting of context, sampling, methods, and researcher reflexivity in qualitative studies. Future qualitative studies should report their methods clearly and include reflection on the researchers' roles in the study and how this may have impacted on the process and results of the study. More detail concerning setting and participants is also needed to identify underlying cultural or social phenomena (shared values or beliefs) that mediate the influence of communications, as these need to be addressed when designing targeted digital health interventions. A better representation of the participant’s voice in the data in the studies included in this synthesis could have improved our confidence in some of the findings. For example, in some studies quotes were not labelled with a participant identifier, so we were unable to determine if the quotes came from multiple participants or the same participant.

Research about digital targeted client communication should aim to include a broader spectrum of participants in relation to phone ownership, literacy, and ability to use a smartphone. Researchers could also focus on exploring why some digital health interventions do or do not influence participants' actions and behaviour.

More research is needed on the public’s preferences around the details of timing, amount, and content of digital health interventions from people who have actually participated in digital health interventions. There is a large body of hypothetical studies, and there are some studies that evaluate or discuss participants' experiences after participating in a pilot project or a research trial. However, we found only one study that interviewed participants involved in a digital health intervention that was being delivered on a national scale, in Ghana (Entsieh 2015).

Trials assessing the effectiveness of digital targeted client communication interventions should consider the issues identified in this qualitative evidence synthesis (Table 8 above) and should ensure that the design and assessment of the intervention are properly reported, for instance following existing reporting guidelines for digital health interventions (Agarwal 2016).

## What's new

**Table:** 

**Date**	**Event**	**Description**
16 October 2019	Amended	Typographical error corrected in abstract.

## Appendices

### Appendix 1. List of targeted client communication topics

**Table:**

**For adolescent and youth populations as potential users of SRH services**	**For adult populations as potential users of SRH services**	**For pregnant and postpartum women (up to 6 weeks)**	**For pregnant and postpartum women (up to 6 weeks) living with HIV**	**Parents and other caregivers of children under 5 years of age**
Family planning/ contraception Sexual violence Prevention, diagnosis, and treatment of STIs, including HIV Screening for cervical and breast cancer Folic acid fortification Infertility Safe abortion HPV vaccination Comprehensive sexual education Puberty	Family planning/ contraception Sexual violence Prevention, diagnosis, and treatment of STIs, including HIV Screening for cervical and breast cancer Infertility Safe abortion	Antenatal care Birth preparedness Skilled attendant at birth Emergency obstetric care Postpartum care Kangaroo Mother Care Tetanus immunization Anemia prevention and control STI testing and treatment in pregnancy Sexual violence Malaria prevention and treatment Smoking cessation during pregnancy	Antenatal care Birth preparedness Skilled attendant at birth Emergency obstetric care Postpartum care Kangaroo mother care Tetanus immunisation Anaemia prevention and control STI testing and treatment in pregnancy Sexual violence Malaria prevention and treatment ARV adherence Early infant diagnosis Retention of mother and infant pairs in eMTCT care	Postnatal care Immunisation Breastfeeding Integrated management of newborn and childhood illnesses (IMNCI) Water, sanitation, and hygiene (WASH) Management of diarrhoeal illnesses, oral rehydration solution, zinc Growth monitoring and nutrition Early infant diagnosis in HIV‐exposed children; ARV therapy for HIV‐exposed and HIV‐infected children Early childhood development
Abbreviations: ARV: antiretroviral; eMTCT: elimination of mother‐to‐child transmission; HPV: human papillomavirus; SRH: sexual and reproductive health; STI: sexually transmitted infection				

### Appendix 2. Search strategy

#### Ovid MEDLINE® In‐Process & Other Non‐Indexed Citations, Ovid MEDLINE® Daily and Ovid MEDLINE® 1946 to Present ‐ Searched 3 July 2017

1 Family Planning Services/ (23966)

2 Contraception/ (18495)

3 Reproductive behavior/ or Contraception behavior/ (8083)

4 exp Contraceptive agents/ (68229)

5 exp Contraceptive Devices/ (23779)

6 (condom* or (OC adj pill) or (depot medroxyprogest* or NET‐EN or NET EN or Mesigyna or Cyclofem) or (intrauterine system or intra‐uterine system or IUS or intrauterine device* or intra‐uterine device* or IUD*) or (vasectomy or sterilisation or sterilization or (tubal adj ligation)) or ((vaginal adj ring) or cycletel or cycle‐tel or ((abstain or abstinen*) adj2 (sex* or intercourse)) or lactational amenorr*)).ti,ab,kw. (55490)

7 (contracept* or family planning or (birth adj (control or regulat* or spacing)) or planned parenthood or ((population or fertility) adj (regulat* or control))).ti,ab,kw. (87955)

8 Pregnancy in Adolescence/ (7459)

9 (pregnan* adj2 (adolescen* or teen* or schoolchild*)).ti,ab,kw. (6428)

10 Pregnancy, unplanned/ or Pregnancy, unwanted/ (3799)

11 (pregnan* adj3 (prevent* or interrupt* or unplanned or unwanted or mistimed)).ti,ab,kw. (12797)

12 exp Sexually Transmitted Diseases/di, dt, ep, pc, px, tm [Diagnosis, Drug Therapy, Epidemiology, Prevention & Control, Psychology, Transmission] (202486)

13 (sexually transmi* or STI or STIs or STD or STDs or venereal).ti,ab,kw. (43203)

14 exp HIV Infections/di, dt, ep, pc, px, tm [Diagnosis, Drug Therapy, Epidemiology, Prevention & Control, Psychology, Transmission] (171444)

15 HIV Seropositivity/dt, ep, pc, px, tm [Drug Therapy, Epidemiology, Prevention & Control, Psychology, Transmission] (8236)

16 (Anti‐HIV Agents/ or Antiretroviral Therapy, Highly Active/) and Medication Adherence/ (1671)

17 (hiv or hiv‐1* or hiv‐2* or hiv1 or hiv2 or human immunodeficiency virus or human immunedeficiency virus or human immuno‐deficiency virus or human immune‐deficiency virus or (human immun* and deficiency virus) or acquired immunodeficiency syndrome or acquired immunedeficiency syndrome or acquired immuno‐deficiency syndrome or acquired immune‐deficiency syndrome or (acquired immun* and deficiency syndrome)).ti,ab,kw. (303793)

18 ((antiretroviral* or anti‐retroviral* or ARV*) adj2 (complian* or adheren*)).ti,ab,kw. (2178)

19 (Anti‐HIV Agents/ or Antiretroviral Therapy, Highly Active/) and (Infant, Premature/ or Infant, Newborn/ or Infant, Low Birth Weight/ or Infant, Extremely Low Birth Weight/ or Infant, Small for Gestational Age/ or Infant/ or Infant, Very Low Birth Weight/ or Infant, Postmature/ or Infant, Extremely Premature/ or Child/ or Child, Preschool/ or Adolescent/) (8310)

20 ((antiretroviral* or anti‐retroviral* or ARV*) and (infant* or newborn* or neonat* or child* or schoolchild* or adolescen* or teen*)).ti,ab,kw. (7745)

21 Papillomavirus Infections/pc [Prevention & Control] (4874)

22 Papillomavirus Vaccines/ad, tu [Administration & Dosage, Therapeutic Use] (3397)

23 Human Papillomavirus Recombinant Vaccine Quadrivalent, Types 6, 11, 16, 18/ad, tu [Administration & Dosage, Therapeutic Use] (63)

24 ((hpv or papilloma virus* or papillomavirus*) adj2 (vaccinat* or revaccinat* or immuniz* or immunis* or immunother* or inoculat* or innoculat* or prophyla*)).ti,ab,kw. (4505)

25 Domestic Violence/ or Spouse Abuse/ or Intimate Partner Violence/ or Rape/ (18827)

26 (((sexual or domestic or spouse* or intimate partner) adj3 (violen* or abus*)) or rape).ti,ab,kw. (29317)

27 Puberty/ (12795)

28 (pubert* or pubescen*).ti,ab,kw. (35567)

29 Menstruation/ (15555)

30 (menstruat* or menstrual*).ti,ab,kw. (46593)

31 Abortion, Legal/ (7381)

32 Abortion, Induced/ (26890)

33 (abort* or miscarr* or (pregnan* adj2 terminat*)).ti,ab,kw. (90341)

34 Infertility/ (13584)

35 Reproductive Techniques, Assisted/ (8167)

36 Fertilization in Vitro/ (28895)

37 (infertil* or assisted reproductive technolog* or in vitro fertili* or in‐vitro fertili* or IVF).ti,ab,kw. (77929)

38 Sexual behavior/ or Sex work/ or Safe sex/ or Unsafe sex/ (58649)

39 (sex* adj (protected or unprotected or safe or unsafe or risk* or behavio*)).ti,ab,kw. (30802)

40 (Contact tracing/ or Disease notification/) and Sexual partners/ (481)

41 (partner* adj3 (notifi* or tracing or report*)).ti,ab,kw. (4188)

42 Prenatal Care/ (24157)

43 (((antenatal or ante‐natal or prenatal or pre‐natal or antepartum or ante‐partum) adj3 (care or service* or counsel* or test*)) or (birth adj3 prepar*)).ti,ab,kw. (23850)

44 Maternal Health Services/ (12513)

45 ((maternal or mother*) adj3 (health or service* or care)).ti,ab,kw. (23535)

46 Reproductive Health/ (2179)

47 (reproductive adj2 (health or care or service*)).ti,ab,kw. (11640)

48 Midwifery/ (17799)

49 (midwi* or skilled birth or skilled attendan*).ti,ab,kw. (21866)

50 Obstetric Labor Complications/ (16607)

51 Pregnancy Complications/ (85521)

52 ((obstetric* or pregnan* or labour or labor or parturition) adj3 (emergenc* or complication*)).ti,ab,kw. (19569)

53 Postnatal Care/ (4855)

54 Perinatal Care/ (3758)

55 Postpartum Period/ (22402)

56 ((postnatal or post‐natal or perinatal or peri‐natal or postpartum or post‐partum) adj2 (care or service*)).ti,ab,kw. (5608)

57 Maternal Nutritional Physiological Phenomena/ (3266)

58 Prenatal Nutritional Physiological Phenomena/ (1578)

59 Breast Feeding/ (34309)

60 (breast feed* or breast fed or breastfeed* or breastfed).ti,ab,kw. (36816)

61 (Infant, Premature/ or Infant, Newborn/ or Infant, Low Birth Weight/ or Infant, Extremely Low Birth Weight/ or Infant, Small for Gestational Age/ or Infant/ or Infant, Very Low Birth Weight/ or Infant, Postmature/ or Infant, Extremely Premature/) and Early Diagnosis/ (2250)

62 (early adj1 diagnos* adj2 (infant* or neonat* or newborn*)).ti,ab,kw. (378)

63 diagnosis.fs. and (infant* or neonat* or newborn*).ti,ab,kw. (83646)

64 *"Infectious Disease Transmission, Vertical"/ (8460)

65 ((mother‐to‐child transmi* adj3 (prevent* or eliminat*)) or emtct or pmtct or (vertical adj transmi*)).ti,ab,kw. (7627)

66 (Immunization/ or Immunization, passive/ or Immunization schedule/ or Immunization, secondary/ or Immunization Programs/ or Vaccination/ or Mass vaccination/) and (Infant, Premature/ or Infant, Newborn/ or Infant, Low Birth Weight/ or Infant, Extremely Low Birth Weight/ or Infant, Small for Gestational Age/ or Infant/ or Infant, Very Low Birth Weight/ or Infant, Postmature/ or Infant, Extremely Premature/ or Child/ or Child, Preschool/ or Adolescent/ or Pregnancy/) (43689)

67 ((immuniz* or immunis* or vaccinat*) and (infant* or newborn* or neonat* or child* or adolescen* or teen*)).ti,ab,kw. (45220)

68 Child health services/ or Maternal‐child health services/ (19872)

69 "Delivery of Health Care, Integrated"/ (10591)

70 ((integrat* adj3 (health care or healthcare or management or treat* or service*) adj3 (child* or schoolchild* or infant* or neonat* or newborn or adolescen* or teen*)) or IMNCI).ti,ab,kw. (886)

71 (Diarrhea/di, dt, ep, pc, th, tm or Diarrhea, Infantile/di, dt, ep, pc, th, tm) and (Infant, Premature/ or Infant, Newborn/ or Infant, Low Birth Weight/ or Infant, Extremely Low Birth Weight/ or Infant, Small for Gestational Age/ or Infant/ or Infant, Very Low Birth Weight/ or Infant, Postmature/ or Infant, Extremely Premature/ or Child/ or Child, Preschool/ or Adolescent/) (10175)

72 (diarrh* and (infant* or newborn* or neonat* or child* or schoolchild* or adolescen* or teen*)).ti,ab,kw. (25409)

73 Hand Hygiene/ or Hand Disinfection/ (5710)

74 Water Supply/ (30922)

75 Drinking Water/ (5420)

76 Sanitation/ (6553)

77 (handwash* or hand‐wash* or (wash* adj1 hand*) or hand hygiene or hand‐hygiene or soap or water suppl* or sanitation or sanitary or drinking water or potable water).ti,ab,kw. (79227)

78 Fluid Therapy/ and (Infant, Premature/ or Infant, Newborn/ or Infant, Low Birth Weight/ or Infant, Extremely Low Birth Weight/ or Infant, Small for Gestational Age/ or Infant/ or Infant, Very Low Birth Weight/ or Infant, Postmature/ or Infant, Extremely Premature/ or Child/ or Child, Preschool/) (4512)

79 (oral rehydration adj (solution* or salt* or therapy)).ti,ab,kw. (2171)

80 Child Development/ or Adolescent Development/ (43788)

81 ((child* or schoolchild* or adolescen* or teen*) adj2 (develop* or progress*)).ti,ab,kw. (48148)

82 Breast Neoplasms/di, dg, pc or (Breast Neoplasms/ and Mass Screening/) (59944)

83 Uterine Cervical Neoplasms/di, dg, pc [Diagnosis, Diagnostic Imaging, Prevention & Control] (22428)

84 (((breast or cervix or cervical) adj (neoplasm* or cancer*)) and (screen* or diagnos*)).ti,ab,kw. (70367)

85 Folic Acid/ad, tu, th [Administration & Dosage, Therapeutic Use, Therapy] (8348)

86 Folic Acid Deficiency/dt, pc, th [Drug Therapy, Prevention & Control, Therapy] (803)

87 (folic acid adj (fortif* or supplement* or treat* or therap*)).ti,ab,kw. (3090)

88 Sex Education/ (8462)

89 (sex* adj (educat* or "health promot*")).ti,ab,kw. (8482)

90 Pregnancy in Adolescence/ (7459)

91 Kangaroo‐Mother Care Method/ (220)

92 (kangaroo adj2 (mother or infant or care)).ti,ab,kw. (540)

93 (Anemia/dt, pc or Anemia, Hypochromic/dt, pc or Anemia, Iron‐Deficiency/dt, pc) and Pregnancy/ (1420)

94 ((maternal or mother* or pregnan*) adj2 (nutrition* or folate or folic or iron or anaemi* or anemi*)).ti,ab,kw. (8929)

95 (Malaria/di, dt, pc or Malaria, Falciparum/di, dt, pc or Malaria, Vivax/di, dt, pc) and (Pregnancy/ or Pregnancy Complications, Parasitic/) (1999)

96 ((malaria* or falciparum or vivax) adj3 (pregnan* or mother* or maternal or postpartum or post partum)).ti,ab,kw. (2187)

97 Smoking Cessation/ and (Pregnancy/ or Pregnancy in Adolescence/) (1515)

98 (((smoking or smoker* or cigarette or tobacco) adj3 (ceas* or cessation or stop* or discontinu*)) and (pregnan* or maternal or mother*)).ti,ab,kw. (1895)

99 Mental health/ or Mental disorders/ or Mental health services/ or Community mental health services/ (206414)

100 Maternal behavior/ or Mother‐child relations/ or Parenting/ or Paternal behavior/ (38878)

101 Depression, Postpartum/ (4465)

102 (((mental or behavio*) adj3 (health or disorder*)) or postpartum depression or post‐partum depression).ti,ab,kw. (186868)

103 or/1‐102 (1885303)

104 Cell Phones/ (7022)

105 Smartphone/ (1162)

106 MP3‐Player/ (167)

107 Computers, Handheld/ (3076)

108 ((cell* or mobile*) adj1 (phone* or telephone* or technolog* or device*)).ti,ab,kw. (12925)

109 (handheld or hand‐held).ti,ab,kw. (9885)

110 (smartphone* or smart‐phone* or cellphone* or mobiles).ti,ab,kw. (5371)

111 ((personal adj1 digital) or (PDA adj3 (device* or assistant*)) or MP3 player* or MP4 player*).ti,ab,kw. (1291)

112 (samsung or nokia).ti,ab,kw. (808)

113 (windows adj3 (mobile* or phone*)).ti,ab,kw. (42)

114 android.ti,ab,kw. (1508)

115 (ipad* or i‐pad* or ipod* or i‐pod* or iphone* or i‐phone*).ti,ab,kw. (1927)

116 (tablet* adj3 (device* or computer*)).ti,ab,kw. (974)

117 Telemedicine/ (16542)

118 Videoconferencing/ or Webcasts as topic/ (1479)

119 Text Messaging/ (1630)

120 Telenursing/ (173)

121 (mhealth or m‐health or "mobile health" or ehealth or e‐health or "electronic health").ti,ab,kw. (15150)

122 (telemedicine or tele‐medicine or telehealth or tele‐health or telecare or tele‐care or telenursing or tele‐nursing or telepsychiatry or tele‐psychiatry or telemonitor* or tele‐monitor* or teleconsult* or tele‐consult* or telecounsel* or tele‐counsel* or telecoach* or tele‐coach*).ti,ab,kw. (13744)

123 (videoconferenc* or video‐conferenc* or webcast* or web‐cast*).ti,ab,kw. (2476)

124 (((text* or short or voice or multimedia or multi‐media or electronic or instant) adj1 messag*) or instant messenger).ti,ab,kw. (3373)

125 (texting or texted or texter* or ((sms or mms) adj (service* or messag*)) or interactive voice response* or IVR or voice call* or callback* or voice over internet or VOIP).ti,ab,kw. (2519)

126 (Facebook or Twitter or Whatsapp* or Skyp* or YouTube or "You Tube" or Google Hangout*).ti,ab,kw. (4017)

127 Mobile Applications/ (2148)

128 "mobile app*".ti,ab,kw. (1671)

129 Social Media/ (3725)

130 (social adj (media or network*)).ti,ab,kw. (15779)

131 Reminder Systems/ (3035)

132 (remind* adj3 (text* or system* or messag*)).ti,ab,kw. (1388)

133 Electronic Mail/ (2399)

134 (electronic mail* or email* or e‐mail or webmail).ti,ab,kw. (11345)

135 Medical informatics/ or Medical informatics applications/ (12810)

136 Nursing informatics/ or Public health informatics/ (2455)

137 ((medical or clinical or health or healthcare or nurs*) adj3 informatics).ti,ab,kw. (5000)

138 Multimedia/ (1784)

139 Hypermedia/ (398)

140 Blogging/ (807)

141 (multimedia or multi‐media or hypermedia or hyper‐media or blog* or vlog* or weblog* or web‐log*).ti,ab,kw. (6134)

142 Interactive Tutorial/ (247)

143 Computer‐Assisted Instruction/ (11225)

144 ((interactive or computer‐assisted) adj1 (tutor* or technolog* or learn* or instruct* or software or communication)).ti,ab,kw. (2208)

145 or/104‐144 (132547)

146 103 and 145 (15436)

147 Qualitative Research/ (34710)

148 Interview/ (27953)

149 (theme$ or thematic).mp. (73915)

150 qualitative.af. (183873)

151 Nursing Methodology Research/ (16939)

152 questionnaire$.mp. (600933)

153 ethnological research.mp. (7)

154 ethnograph$.mp. (8388)

155 ethnonursing.af. (139)

156 phenomenol$.af. (21166)

157 (grounded adj (theor$ or study or studies or research or analys?s)).af. (9080)

158 (life stor$ or women* stor$).mp. (1094)

159 (emic or etic or hermeneutic$ or heuristic$ or semiotic$).af. or (data adj1 saturat$).tw. or participant observ$.tw. (18540)

160 (social construct$ or (postmodern$ or post‐structural$) or (post structural$ or poststructural$) or post modern$ or post‐modern$ or feminis$ or interpret$).mp. (455073)

161 (action research or cooperative inquir$ or co operative inquir$ or co‐operative inquir$).mp. (3372)

162 (humanistic or existential or experiential or paradigm$).mp. (124350)

163 (field adj (study or studies or research)).tw. (13705)

164 human science.tw. (251)

165 biographical method.tw. (15)

166 theoretical sampl$.af. (530)

167 ((purpos$ adj4 sampl$) or (focus adj group$)).af. (47292)

168 (life world or life‐world or conversation analys?s or personal experience$ or theoretical saturation).mp. (13506)

169 ((lived or life) adj experience$).mp. (8082)

170 cluster sampl$.mp. (5645)

171 observational method$.af. (586)

172 content analysis.af. (18829)

173 (constant adj (comparative or comparison)).af. (3524)

174 ((discourse$ or discurs$) adj3 analys?s).tw. (1693)

175 narrative analys?s.af. (871)

176 heidegger$.tw. (585)

177 colaizzi$.tw. (513)

178 spiegelberg$.tw. (77)

179 (van adj manen$).tw. (320)

180 (van adj kaam$).tw. (40)

181 (merleau adj ponty$).tw. (187)

182 husserl$.tw. (222)

183 foucault$.tw. (687)

184 (corbin$ adj2 strauss$).tw. (254)

185 glaser$.tw. (884)

186 or/147‐185 (1448447)

187 146 and 186 (4951)

188 limit 187 to yr="1993 ‐Current" (4863)

#### Embase (Ovid) 1980 to 2017 Week 27 ‐ Searched 5 July 2017

1 family planning/ (34549)

2 contraception/ (41073)

3 reproductive behavior/ (901)

4 contraceptive behavior/ (2733)

5 exp contraceptive agent/ (129445)

6 exp contraceptive device/ (37567)

7 (condom* or (OC adj pill) or (depot medroxyprogest* or NET‐EN or NET EN or Mesigyna or Cyclofem) or (intrauterine system or intra‐uterine system or IUS or intrauterine device* or intra‐uterine device* or IUD*) or (vasectomy or sterilisation or sterilization or (tubal adj ligation)) or ((vaginal adj ring) or cycletel or cycle‐tel or ((abstain or abstinen*) adj2 (sex* or intercourse)) or lactational amenorr*)).ti,ab,kw. (58260)

8 (contracept* or family planning or (birth adj (control or regulat* or spacing)) or planned parenthood or ((population or fertility) adj (regulat* or control))).ti,ab,kw. (83356)

9 adolescent pregnancy/ (8624)

10 (pregnan* adj2 (adolescen* or teen* or schoolchild*)).ti,ab,kw. (6466)

11 unplanned pregnancy/ (4221)

12 unwanted pregnancy/ (3016)

13 (pregnan* adj3 (prevent* or interrupt* or unplanned or unwanted or mistimed)).ti,ab,kw. (14324)

14 exp sexually transmitted disease/di, dt, ep, pc [Diagnosis, Drug Therapy, Epidemiology, Prevention] (36065)

15 (sexually transmi* or STI or STIs or STD or STDs or venereal).ti,ab,kw. (52215)

16 exp Human immunodeficiency virus infection/di, dt, ep, pc [Diagnosis, Drug Therapy, Epidemiology, Prevention] (161341)

17 (hiv or hiv‐1* or hiv‐2* or hiv1 or hiv2 or human immunodeficiency virus or human immunedeficiency virus or human immuno‐deficiency virus or human immune‐deficiency virus or (human immun* and deficiency virus) or acquired immunodeficiency syndrome or acquired immunedeficiency syndrome or acquired immuno‐deficiency syndrome or acquired immune‐deficiency syndrome or (acquired immun* and deficiency syndrome)).ti,ab,kw. (369396)

18 ((antiretroviral* or anti‐retroviral* or ARV*) adj2 (complian* or adheren*)).ti,ab,kw. (2552)

19 (antiretroviral therapy/ or highly active antiretroviral therapy/) and medication compliance/ (675)

20 (antiretroviral therapy/ or highly active antiretroviral therapy/) and (child/ or infant/ or adolescent/ or newborn/) (4120)

21 ((antiretroviral* or anti‐retroviral* or ARV*) and (infant* or newborn* or neonat* or child* or schoolchild* or adolescen* or teen*)).ti,ab,kw. (10070)

22 papillomavirus infection/pc [Prevention] (2211)

23 Wart virus vaccine/ad, dt [Drug Administration, Drug Therapy] (6026)

24 ((hpv or papilloma virus* or papillomavirus*) adj2 (vaccinat* or revaccinat* or immuniz* or immunis* or immunother* or inoculat* or innoculat* or prophyla*)).ti,ab,kw. (5876)

25 domestic violence/ or battered woman/ or family violence/ or exp partner violence/ (19133)

26 statutory rape/ or acquaintance rape/ or rape/ or marital rape/ (7024)

27 (((sexual or domestic or spouse* or intimate partner) adj3 (violen* or abus*)) or rape).ti,ab,kw. (35500)

28 puberty/ or menarche/ (31168)

29 (pubert* or pubescen*).ti,ab,kw. (44006)

30 menstruation/ (18624)

31 (menstruat* or menstrual*).ti,ab,kw. (51352)

32 abortion/ or imminent abortion/ or recurrent abortion/ or septic abortion/ or spontaneous abortion/ (60269)

33 (abort* or miscarr* or (pregnan* adj2 terminat*)).ti,ab,kw. (100665)

34 infertility/ (36051)

35 infertility therapy/ or in vitro fertilization/ (18622)

36 (infertil* or assisted reproductive technolog* or in vitro fertili* or in‐vitro fertili* or IVF).ti,ab,kw. (111090)

37 sexual behavior/ or adolescent sexual behavior/ or casual sex/ or prostitution/ or exp safe sex/ or sexual practice/ or exp unsafe sex/ (105056)

38 (sex* adj (protected or unprotected or safe or unsafe or risk* or behavio*)).ti,ab,kw. (31641)

39 contact examination/ (3153)

40 (partner* adj3 (notifi* or tracing or report*)).ti,ab,kw. (5253)

41 prenatal care/ or prenatal screening/ (39325)

42 (((antenatal or ante‐natal or prenatal or pre‐natal or antepartum or ante‐partum) adj3 (care or service* or counsel* or test*)) or (birth adj3 prepar*)).ti,ab,kw. (29229)

43 maternal health service/ (427)

44 ((maternal or mother*) adj3 (health or service* or care or welfare)).ti,ab,kw. (26499)

45 reproductive health/ (13050)

46 (reproductive adj2 (health or care or service*)).ti,ab,kw. (14894)

47 midwife/ or nurse midwife/ (27896)

48 (midwi* or skilled birth or skilled attendan*).ti,ab,kw. (23691)

49 labor complication/ (9201)

50 pregnancy complication/ (71151)

51 ((obstetric* or pregnan* or labour or labor or parturition) adj3 (emergenc* or complication*)).ti,ab,kw. (30187)

52 postnatal care/ or newborn care/ (16297)

53 perinatal care/ (12694)

54 maternal care/ or maternal welfare/ (26845)

55 maternal nutrition/ (9782)

56 puerperium/ (31726)

57 ((postnatal or post‐natal or perinatal or peri‐natal or postpartum or post‐partum) adj2 (care or service*)).ti,ab,kw. (6938)

58 breast feeding/ (42834)

59 (breast feed* or breast fed or breastfeed* or breastfed).ti,ab,kw. (42934)

60 early diagnosis/ and (exp infant/ or newborn/) (5547)

61 (early adj1 diagnos* adj2 (infant* or neonat* or newborn*)).ti,ab,kw. (504)

62 diagnosis.fs. and (infant* or neonat* or newborn*).ti,ab,kw. (103248)

63 vertical transmission/ (12627)

64 ((mother‐to‐child transmi* adj3 (prevent* or eliminat*)) or emtct or pmtct or (vertical adj transmi*)).ti,ab,kw. (9576)

65 (immunization/ or mass immunization/ or vaccination/) and (exp infant/ or newborn/ or exp child/ or adolescent/ or pregnancy/) (43651)

66 ((immuniz* or immunis* or vaccinat*) and (infant* or newborn* or neonat* or child* or adolescen* or teen* or pregnan*)).ti,ab,kw. (55411)

67 child health care/ or early childhood intervention/ or maternal child health care/ (36574)

68 integrated health care system/ (9135)

69 ((integrat* adj3 (health care or healthcare or management or treat* or service*) adj3 (child* or schoolchild* or infant* or neonat* or newborn or adolescen* or teen*)) or IMNCI).ti,ab,kw. (976)

70 diarrhea/di, dm, dt, ep, pc, th [Diagnosis, Disease Management, Drug Therapy, Epidemiology, Prevention, Therapy] (21647)

71 infantile diarrhea/di, dm, dt, ep, pc, th [Diagnosis, Disease Management, Drug Therapy, Epidemiology, Prevention, Therapy] (1735)

72 (diarrhea/di, dm, dt, ep, pc, th or infantile diarrhea/di, dm, dt, ep, pc, th) and (exp infant/ or newborn/ or exp child/ or adolescent/ or pregnancy/) (7720)

73 (diarrh* and (infant* or newborn* or neonat* or child* or schoolchild* or adolescen* or teen*)).ti,ab,kw. (29397)

74 hand washing/ or hand disinfection/ (11356)

75 water supply/ (32264)

76 drinking water/ (38027)

77 sanitation/ (12723)

78 (handwash* or hand‐wash* or (wash* adj1 hand*) or hand hygiene or hand‐hygiene or soap or water suppl* or sanitation or sanitary or drinking water or potable water).ti,ab,kw. (93230)

79 oral rehydration therapy/ (2408)

80 (oral rehydration adj (solution* or salt* or therapy)).ti,ab,kw. (2242)

81 child development/ or adolescent development/ (43677)

82 ((child* or schoolchild* or adolescen* or teen*) adj2 (develop* or progress*)).ti,ab,kw. (53455)

83 breast cancer/di, dm, dt, pc [Diagnosis, Disease Management, Drug Therapy, Prevention] (95144)

84 breast cancer/ and cancer screening/ (15395)

85 uterine cervix cancer/di, dm, dt, pc [Diagnosis, Disease Management, Drug Therapy, Prevention] (16779)

86 (((breast or cervix or cervical) adj (neoplasm* or cancer*)) and (screen* or diagnos*)).ti,ab,kw. (107649)

87 folic acid/ad, dt [Drug Administration, Drug Therapy] (11535)

88 folic acid deficiency/dm, dt, pc, th [Disease Management, Drug Therapy, Prevention, Therapy] (1168)

89 (folic acid adj (fortif* or supplement* or treat* or therap*)).ti,ab,kw. (3883)

90 sexual education/ (10473)

91 (sex* adj (educat* or "health promot*")).ti,ab,kw. (8361)

92 kangaroo care/ (706)

93 (kangaroo adj2 (mother or infant or care)).ti,ab,kw. (718)

94 (anemia/dt, pc or iron deficiency anemia/dt, pc) and pregnancy/ (1207)

95 ((maternal or mother* or pregnan*) adj2 (nutrition* or folate or folic or iron or anaemi* or anemi*)).ti,ab,kw. (9114)

96 (malaria/di, dm, dt, pc or malaria, falciparum/di, dm, dt, pc or malaria, vivax/di, dm, dt, pc) and (pregnancy/ or pregnancy complication/) (1486)

97 ((malaria* or falciparum or vivax) adj3 (pregnan* or mother* or maternal or postpartum or post partum)).ti,ab,kw. (2651)

98 smoking cessation/ and (pregnancy/ or adolescent pregnancy/) (1931)

99 (((smoking or smoker* or cigarette or tobacco) adj3 (ceas* or cessation or stop* or discontinu*)) and (pregnan* or maternal or mother*)).ti,ab,kw. (2272)

100 mental health/ or community mental health/ or mental health service/ (147416)

101 maternal behavior/ or parental behavior/ or paternal behavior/ (22210)

102 puerperal depression/ (8330)

103 (((mental or behavio*) adj3 (health or disorder*)) or postpartum depression or post‐partum depression or postnatal depression or post‐natal depression).ti,ab,kw. (235408)

104 or/1‐103 (2249116)

105 mobile phone/ or smartphone/ (15726)

106 mp3 player/ (160)

107 ((cell* or mobile*) adj1 (phone* or telephone* or technolog* or device*)).ti,ab,kw. (16368)

108 (handheld or hand‐held).ti,ab,kw. (12981)

109 (smartphone* or smart‐phone* or cellphone* or mobiles).ti,ab,kw. (7417)

110 ((personal adj1 digital) or (PDA adj3 (device* or assistant*)) or MP3 player* or MP4 player*).ti,ab,kw. (1683)

111 (samsung or nokia).ti,ab,kw. (1425)

112 (windows adj3 (mobile* or phone*)).ti,ab,kw. (61)

113 android.ti,ab,kw. (2314)

114 (ipad* or i‐pad* or ipod* or i‐pod* or iphone* or i‐phone*).ti,ab,kw. (3494)

115 (tablet* adj3 (device* or computer*)).ti,ab,kw. (1535)

116 telemedicine/ or telecardiology/ or teleconsultation/ or teledermatology/ or telediagnosis/ or telemonitoring/ or telepathology/ or telepsychiatry/ or teleradiotherapy/ or telesurgery/ or teletherapy/ (27082)

117 videoconferencing/ or webcast/ (2779)

118 text messaging/ (2815)

119 telenursing/ (201)

120 (mhealth or m‐health or "mobile health" or ehealth or e‐health or "electronic health").ti,ab,kw. (19120)

121 (telemedicine or tele‐medicine or telehealth or tele‐health or telecare or tele‐care or telenursing or tele‐nursing or telepsychiatry or tele‐psychiatry or telemonitor* or tele‐monitor* or teleconsult* or tele‐consult* or telecounsel* or tele‐counsel* or telecoach* or tele‐coach*).ti,ab,kw. (17396)

122 (videoconferenc* or video‐conferenc* or webcast* or web‐cast*).ti,ab,kw. (3291)

123 (((text* or short or voice or multimedia or multi‐media or electronic or instant) adj1 messag*) or instant messenger).ti,ab,kw. (4409)

124 (texting or texted or texter* or ((sms or mms) adj (service* or messag*)) or interactive voice response* or IVR or voice call* or callback* or voice over internet or VOIP).ti,ab,kw. (3476)

125 (Facebook or Twitter or Whatsapp* or Skyp* or YouTube or "You Tube" or Google Hangout*).ti,ab,kw. (5764)

126 mobile application/ (4307)

127 "mobile app*".ti,ab,kw. (1967)

128 social media/ (8882)

129 (social adj (media or network*)).ti,ab,kw. (20412)

130 reminder system/ (2115)

131 (remind* adj3 (text* or system* or messag*)).ti,ab,kw. (1924)

132 e‐mail/ (14658)

133 (electronic mail* or email* or e‐mail or webmail).ti,ab,kw. (22106)

134 medical informatics/ (17675)

135 nursing informatics/ (1272)

136 ((medical or clinical or health or healthcare or nurs*) adj3 informatics).ti,ab,kw. (6985)

137 multimedia/ (3162)

138 hypermedia/ (368)

139 blogging/ (135)

140 (multimedia or multi‐media or hypermedia or hyper‐media or blog* or vlog* or weblog* or web‐log*).ti,ab,kw. (8936)

141 teaching/ (79979)

142 ((interactive or computer‐assisted) adj1 (tutor* or technolog* or learn* or instruct* or software or communication)).ti,ab,kw. (3036)

143 or/105‐142 (250953)

144 104 and 143 (27712)

145 qualitative analysis/ or qualitative research/ (91734)

146 interview/ (163414)

147 (theme$ or thematic).mp. (90768)

148 qualitative.af. (231824)

149 nursing methodology research/ (14443)

150 questionnaire$.mp. (730111)

151 ethnological research.mp. (8)

152 ethnograph$.mp. (9657)

153 ethnonursing.af. (103)

154 phenomenol$.af. (25758)

155 (grounded adj (theor$ or study or studies or research or analys?s)).af. (10886)

156 (life stor$ or women* stor$).mp. (1335)

157 (emic or etic or hermeneutic$ or heuristic$ or semiotic$).af. or (data adj1 saturat$).tw. or participant observ$.tw. (20697)

158 (social construct$ or (postmodern$ or post‐structural$) or (post structural$ or poststructural$) or post modern$ or post‐modern$ or feminis$ or interpret$).mp. (395594)

159 (action research or cooperative inquir$ or co operative inquir$ or co‐operative inquir$).mp. (4053)

160 (humanistic or existential or experiential or paradigm$).mp. (151958)

161 (field adj (study or studies or research)).tw. (15406)

162 human science.tw. (267)

163 biographical method.tw. (19)

164 theoretical sampl$.af. (692)

165 ((purpos$ adj4 sampl$) or (focus adj group$)).af. (52066)

166 (life world or life‐world or conversation analys?s or personal experience$ or theoretical saturation).mp. (38320)

167 ((lived or life) adj experience$).mp. (10021)

168 cluster sampl$.mp. (7024)

169 observational method$.af. (1937)

170 content analysis.af. (23013)

171 (constant adj (comparative or comparison)).af. (4144)

172 ((discourse$ or discurs$) adj3 analys?s).tw. (1875)

173 narrative analys?s.af. (1004)

174 heidegger$.tw. (644)

175 colaizzi$.tw. (563)

176 spiegelberg$.tw. (95)

177 (van adj manen$).tw. (338)

178 (van adj kaam$).tw. (34)

179 (merleau adj ponty$).tw. (193)

180 husserl$.tw. (266)

181 foucault$.tw. (707)

182 (corbin$ adj2 strauss$).tw. (266)

183 glaser$.tw. (828)

184 or/145‐183 (1691925)

185 144 and 184 (8529)

186 limit 185 to yr="1993 ‐Current" (8389)

187 limit 186 to embase (3633)

#### WHO Global Health Library – Searched 6 July 2017

(tw:((cell* OR mobile*) AND (phone* OR telephone* OR technolog* OR device*)) OR smartphone* OR smart‐phone* OR cellphone* OR mobiles OR mhealth OR m‐health OR "mobile health" OR ehealth OR e‐health OR "electronic health" OR telemedicine OR tele‐medicine OR telehealth OR tele‐health OR telecare OR tele‐care OR telenursing OR tele‐nursing OR telepsychiatry OR tele‐psychiatry OR telemonitor* OR tele‐monitor* OR teleconsult* OR tele‐consult* OR telecounsel* OR tele‐counsel* OR telecoach* OR tele‐coach* OR videoconferenc* OR video‐conferenc* OR webcast* OR web‐cast* OR ((text* OR short OR voice OR multimedia OR multi‐media OR electronic OR instant) AND messag*) OR instant messenger OR texting OR texted OR texter* OR ((sms OR mms) AND (service* OR messag*)) OR interactive voice response* OR ivr OR voice call* OR callback* OR voice over internet OR voip OR "mobile app*" OR (social AND (media OR network*)) OR ((medical OR clinical OR health OR healthcare OR nurs*) AND informatics)) OR mj:("Telemedicine" OR "Cell Phones" OR "Internet" OR "Mobile Applications" OR "Medical Informatics" OR "Information Technology" OR "Smartphone")) AND (instance:"ghl") AND ( db:("LILACS" OR "WPRIM" OR "WHOLIS" OR "IMEMR" OR "AIM") AND year_cluster:("2015" OR "2013" OR "2014" OR "2005" OR "2007" OR "2006" OR "2011" OR "2012" OR "2009" OR "2001" OR "2003" OR "2010" OR "2016" OR "2008" OR "2002" OR "2000" OR "1998" OR "2004" OR "1999" OR "1997" OR "1996" OR "1995" OR "1993" OR "2017") AND tw:(qualitative OR interview* OR focus group* OR questionnaire* OR ethnograph* OR perception* OR perceiv* OR opinion* OR attitude* OR view* OR experienc* OR sceptic* OR skeptic* OR dilemma* OR "social mobilisation" OR "social mobilization" OR complian* OR refus* OR feeling* OR impression* OR belief* OR trust OR accept* OR knowledge OR comprehension OR understanding OR aware* OR ( (communit* OR social OR patient* ) AND ( behavi* OR integrat* OR program* OR support* OR norm OR norms OR leader* OR advoca* OR information OR action OR need OR needs OR influenc* OR complian* OR participat* ) )) = 1411 hits

#### POPLINE – Searched 6 July 2017

Keyword:(TEXT MESSAGING OR MOBILE DEVICES OR INFORMATION COMMUNICATION TECHNOLOGY OR CELLULAR PHONE) AND All Fields: (qualitative OR interview OR interviews OR interviewing OR focus group OR focus groups OR questionnaire OR questionnaires OR ethnography OR ethnographic OR perception OR perceptions OR perceive OR perceives OR perceived OR opinion OR opinions OR attitude OR attitudes OR view OR views OR experience OR experiences OR experienced OR experiencing OR sceptic OR sceptical OR skeptic OR skeptical OR dilemma OR dilemmas OR "social mobilisation" OR "social mobilization" OR compliant OR compliance OR refuse OR refusal OR refused OR refusing OR feeling OR feelings OR impression OR impressions OR belief OR beliefs OR trust OR accept OR acceptance OR accepting OR accepted OR knowledge OR comprehension OR understanding OR aware OR awareness OR ( (community OR communities OR social OR patient OR patients OR client OR clients ) AND ( behavior OR behaviour OR behaviors OR behaviours OR behavioral OR behavioural OR integrate OR integration OR program OR programs OR programme OR programmes OR support OR supporting OR supported OR norm OR norms OR leader OR leaders OR leadership OR advocate OR advocated OR advocacy OR information OR action OR need OR needs OR influence OR influences OR influenced OR influencing OR participate OR participates OR participated OR participation ) ))

OR

All Fields:((cell OR cellular OR mobile) AND (phone OR phones OR telephone OR telephones OR technology OR technologies OR device OR devices)) OR smartphone OR smartphones OR smart‐phone OR smart‐phones OR cellphone OR cellphones OR mobiles OR mhealth OR m‐health OR "mobile health" OR ehealth OR e‐health OR "electronic health" OR telemedicine OR tele‐medicine OR telehealth OR tele‐health OR telecare OR tele‐care OR telenursing OR tele‐nursing OR telepsychiatry OR tele‐psychiatry OR telemonitor OR telemonitoring OR tele‐monitor OR tele‐monitoring OR teleconsult OR teleconsulting OR tele‐consult OR tele‐consulting OR telecounsel OR telecounseling OR tele‐counsel OR tele‐counseling OR telecoach OR telecoaching OR tele‐coach OR tele‐coaching OR videoconference OR videoconferences OR videoconferencing OR video‐conference OR video‐conferences OR video‐conferencingOR webcast OR webcasts OR webcasting OR web‐cast OR web‐casts OR web‐casting OR ((text OR texts OR texting OR short OR voice OR multimedia OR multi‐media OR electronic OR instant) AND (message OR messages OR messaging) OR instant messenger OR texting OR texted OR texter OR texters OR ((sms OR mms) AND (service OR services OR message OR messages OR messaging)) OR interactive voice response OR interactive voice responses OR ivr OR voice call OR voice calls OR callback OR voice over internet OR voip OR "mobile app" OR "mobile application" OR "mobile applications" OR (social AND (media OR network* OR networks OR networking)) OR ((medical OR clinical OR health OR healthcare OR nurse OR nurses OR nursing) AND informatics)) AND (qualitative OR interview OR interviews OR interviewing OR focus group OR focus groups OR questionnaire OR questionnaires OR ethnography OR ethnographic OR perception OR perceptions OR perceive OR perceives OR perceived OR opinion OR opinions OR attitude OR attitudes OR view OR views OR experience OR experiences OR experienced OR experiencing OR sceptic OR sceptical OR skeptic OR skeptical OR dilemma OR dilemmas OR "social mobilisation" OR "social mobilization" OR compliant OR compliance OR refuse OR refusal OR refused OR refusing OR feeling OR feelings OR impression OR impressions OR belief OR beliefs OR trust OR accept OR acceptance OR accepting OR accepted OR knowledge OR comprehension OR understanding OR aware OR awareness OR ( (community OR communities OR social OR patient OR patients OR client OR clients ) AND ( behavior OR behaviour OR behaviors OR behaviours OR behavioral OR behavioural OR integrate OR integration OR program OR programs OR programme OR programmes OR support OR supporting OR supported OR norm OR norms OR leader OR leaders OR leadership OR advocate OR advocated OR advocacy OR information OR action OR need OR needs OR influence OR influences OR influenced OR influencing OR participate OR participates OR participated OR participation ) )) – 1381 hits (1993‐2017)

### Appendix 3. Data richness scale table

**Table:**

**Score **	**Measure **	**Example **
**1**	Very little qualitative data presented that relate to the synthesis objective. Those findings that are presented are fairly descriptive.	For example, a mixed‐methods study using open‐ended survey questions or a more detailed qualitative study where only part of the data relates to the synthesis objective
**2**	Some qualitative data presented that relate to the synthesis objective	For example, a limited number of qualitative findings from a mixed‐methods or qualitative study
**3**	A reasonable amount of qualitative data that relate to the synthesis objective	For example, a typical qualitative research article in a journal with a smaller word limit and often using simple thematic analysis
**4**	A good amount and depth of qualitative data that relate to the synthesis objective	For example, a qualitative research article in a journal with a larger word count that includes more context and setting descriptions and a more in‐depth presentation of the findings
**5**	A large amount and depth of qualitative data that relate in depth to the synthesis objective	For example, from a detailed ethnography or a published qualitative article with the same objectives as the synthesis

### Appendix 4. CERQual evidence profiles

**Table:**

**Finding 1**: Overall, participants had a range of views regarding acceptance of the idea of receiving health information through their mobile devices. This was due to factors such as familiarity with the technology, convenience, control, being able to save and re‐read messages later, cost, seeing it as a simple way of providing a reminder for medication or appointments, and the sense that someone was thinking about them and cared enough to send a message.	
**Assessment for each CERQual component**	
Methodological limitations	Moderate concerns about methodological limitations due to poor reporting of participant voices in the findings and researcher reflexivity
Coherence	No or very minor concerns about coherence
Relevance	Moderate concerns about relevance due to a fair number of studies where participants did not experience an mHealth intervention but were asked to comment about their preferences regarding a hypothetical intervention
Adequacy	No or very minor concerns about adequacy
**Overall CERQual assessment**	
Low confidence	Due to moderate concerns regarding methodological limitations and relevance
**Contributing studies**	
**Study**	**Context**
Akinfaderin‐Agarau 2012	Nigeria; adolescent girls and young women; using mobile phones to provide sexual and reproductive health information and services; hypothetical with *no* examples of programme content
Brown 2014	USA; single, adolescent mothers; health promotion information weekly via SMS during the first 6 months postpartum; pilot or implementation study with participation in an mHealth programme
Calderon 2017	Peru; women over 18 who had at least 1 child; SMS‐based mHealth programme on child health; hypothetical with *no* examples of programme content
Cates 2015	USA; middle school students designing text messages to promote HPV vaccine; hypothetical with an example of messages being used
Cornelius 2009	USA; African‐American adolescents; SMS to support HIV/AIDS curriculum; hypothetical with *no* examples of programme content
Curioso 2009	Peru; HIV‐positive adults receiving ART; SMS related to HIV/AIDS; hypothetical with *no* examples of programme content
Evans 2016	UK; African communities; SMS‐based HIV mHealth programme; hypothetical with examples and with *no* examples of programme content
French 2016	UK; young people aged 16 to 24; SMS on sexually transmitted infections; part of an RCT or pilot RCT
Gold 2010	Australia; young people aged 16 to 24; SMS on sexually transmitted infections; part of an RCT or pilot RCT
Greaney 2014	USA; Latina women over the age of 21 needing cancer screening; interactive voice call reminding of screening; hypothetical with an example of messages being used
Hirsch‐Moverman 2017	Lesotho; HIV patients; SMS to provide real‐time adherence support to people on HIV and TB treatment; part of an RCT or pilot RCT
Jalloh‐Vos 2014	Sierra Leone; pregnant and postpartum women and their partners; mobile phone intervention for antenatal care and family planning; part of an RCT or pilot RCT
Jennings 2013	Kenya; HIV‐positive women enrolled in PMTCT and their male partners; SMS reminder for PMTCT testing; hypothetical with an example of messages being used
Lau 2014	South Africa; pregnant women; SMS for antenatal health promotion; part of an RCT or pilot RCT
Mbuagbaw 2012	Cameroon; HIV‐positive patients; SMS for HIV drug adherence; part of an RCT or pilot RCT
Mbuagbaw 2014	Cameroon; individuals living with HIV or involved in HIV support work; community‐owned text messaging programme to support people living with HIV; hypothetical with *no* examples of programme content
Menacho 2013	Peru; men who have sex with men; SMS to motivate for HIV testing; hypothetical with examples and with *no* examples of programme content
Missal 2016	India; husbands of pregnant women 12 to 20 weeks along; voice messages about antenatal care and preparing for delivery; pilot or implementation study with participation in an mHealth programme
Munro 2017	Canada; pregnant or have given birth in the last 12 months; SMS Text4baby programme about prenatal education; hypothetical with an example of messages being used
Naughton 2013	UK; women who smoked during a recent pregnancy; SMS for smoking cessation during pregnancy; pilot or implementation study with participation in an mHealth programme
Odeny 2014	Kenya; women; SMS for early infant HIV testing; hypothetical with *no* examples of programme content
Perry 2012	USA; adolescents aged 15 to 20; SMS with preventative sexual health messages; evaluation or formative research on an existing mHealth programme that the participants have been using
Rana 2015	Uganda; HIV‐positive youth receiving ART; SMS for HIV‐positive youth; Hypothetical with *no* examples of programme content
Rodrigues 2015	India; participants in the intervention arm of the trial; interactive voice recordings and SMS for HIV ART adherence; part of an RCT or pilot RCT
Sloan 2017	UK; women who had received the MiQuit intervention during pregnancy; SMS for smoking cessation during pregnancy; part of an RCT or pilot RCT
Smillie 2014	Canada; HIV‐positive people; SMS about HIV as part of the WelTel BC trial; pilot or implementation study with participation in an mHealth programme
Smith 2017	Cambodia; women who had received an abortion; mobile phone voice messaging and counsellor support for postabortion care; part of an RCT or pilot RCT
Willoughby 2017	USA; college students; SMS for sexual health promotion; hypothetical with an example of messages being used
Wright 2011	USA; African‐American men aged 16 to 20; SMS for HIV prevention; hypothetical with an example of messages being used
Anti retroviral therapy (ARV); Human Papillomavirus (HPV); Prevention of mother‐to‐child transmission (PMTCT); Randomized control trial (RCT); Tuberculosis (TB)	

**Table:**

**Finding 2:** In discussing the pros and cons of digital targeted client communication compared to in‐person meetings with a healthcare provider, some participants perceived interacting with a healthcare provider as preferable, warmer, and something to which they were accustomed. Others also felt that people could receive a faster response using digital communication and that the messages were more convenient and less judgemental. However, some liked having direct access to both healthcare providers and digital targeted client communication.		
**Assessment for each CERQual component**		
Methodological limitations	Minor concerns about methodological limitations due to poor reporting of researcher reflexivity	
Coherence	No or very minor concerns about coherence	
Relevance	Serious concerns about relevance due to a fair number of studies where participants did not experience an mHealth intervention but were asked to comment about their preferences regarding a hypothetical intervention and partial relevance of the target group	
Adequacy	Serious concerns about adequacy due to thin data from a small number of studies	
**Overall CERQual assessment**		
Very low confidence		Due to minor concerns regarding methodological limitations and serious concerns regarding adequacy and relevance
**Contributing studies**		
**Study**		**Context**
Calderon 2017		Peru; women over 18 who had at least 1 child; SMS‐based mHealth programme on child health; hypothetical with *no* examples of programme content
Nachega 2016		South Africa; HIV‐infected pregnant women; SMS about ART adherence to prevent PMTCT; hypothetical with *no* examples of programme content
Naughton 2013		UK; women who smoked during a recent pregnancy; SMS for smoking cessation during pregnancy; pilot or implementation study with participation in an mHealth programme
Sloan 2017		UK; women who had received the MiQuit intervention during pregnancy; SMS for smoking cessation during pregnancy; part of an RCT or pilot RCT
Smillie 2014		Canada; HIV‐positive people; SMS about HIV as part of the WelTel BC trial; pilot or implementation study with participation in an mHealth programme
Anti retroviral therapy (ARV); Prevention of mother‐to‐child transmission (PMTCT)		

**Table:**

**Finding 3:** Participants said that they liked 2‐way digital communication, as this allowed them to engage directly with a healthcare provider, which they trusted more; to receive answers to their questions and have opportunities for discussion; and to receive a more immediate response. However, some participants felt that for some topics they would feel uncomfortable talking to a healthcare provider through a digital channel, due to issues related to shyness and privacy, and would prefer to use SMS.	
**Assessment for each CERQual component**	
Methodological limitations	Moderate concerns about methodological limitations due to poor reporting of sampling, ethical considerations, and researcher reflexivity
Coherence	No or very minor concerns about coherence
Relevance	Serious concerns about relevance due to a large number of studies where participants did not experience an mHealth intervention but were asked to comment about their preferences regarding a hypothetical intervention
Adequacy	Moderate concerns about adequacy due to thin data
**Overall CERQual assessment**	
Very low confidence	Due to moderate concerns regarding methodological limitations and adequacy and serious concerns regarding relevance
**Contributing studies**	
**Study**	**Context**
Akinfaderin‐Agarau 2012	Nigeria; adolescent girls and young women; using mobile phones to provide sexual and reproductive health information and services; hypothetical with *no* examples of programme content
Calderon 2017	Peru; women over 18 who had at least 1 child; SMS‐based mHealth programme on child health; hypothetical with *no* examples of programme content
Cates 2015	USA; middle school students designing text messages to promote HPV vaccine; hypothetical with an example of messages being used
Jennings 2013	Kenya; HIV‐positive women enrolled in PMTCT and their male partners; SMS reminder for PMTCT testing; hypothetical with an example of messages being used
Rana 2015	Uganda; HIV‐positive youth receiving ART; SMS for HIV‐positive youth; Hypothetical with *no* examples of programme content
Rodrigues 2015	India; participants in the intervention arm of the trial; interactive voice recordings and SMS for HIV ART adherence; part of an RCT or pilot RCT
Smillie 2014	Canada; HIV‐positive people; SMS about HIV as part of the WelTel BC trial; pilot or implementation study with participation in an mHealth programme
Smith 2017	Cambodia; women who had received an abortion; mobile phone voice messaging and counsellor support for postabortion care; part of an RCT or pilot RCT
Willoughby 2017	USA; college students; SMS for sexual health promotion; hypothetical with an example of messages being used
Anti retroviral therapy (ART); Human Papillomavirus (HPV); Prevention of mother‐to‐child transmission (PMTCT)	

**Table:**

**Finding 4:** Some participants expressed a concern that some people might view digital targeted communication from healthcare providers as a replacement for seeking appropriate medical assistance, which might have adverse impacts. Whilst some saw digital health as a way to increase access to care, others noted that text messaging might be seen by poorer people as a cheaper or sufficient healthcare option, which might decrease appropriate health‐seeking behaviour.	
**Assessment for each CERQual component**	
Methodological limitations	No or very minor concerns
Coherence	No or very minor concerns
Relevance	Serious concerns due to data from only 1 setting
Adequacy	Serious concerns due to data from only 1 setting
**Overall CERQual assessment**	
Very low confidence	Due to serious concerns regarding relevance and adequacy
**Contributing studies**	
**Study**	**Context**
Willoughby 2017	USA; college students; SMS for sexual health promotion; hypothetical with an example of messages being used

**Table:**

**Finding 5:** Participants reported varying degrees of access to network services, including cell networks (for calls and SMS) and internet. In addition, some had poor access to electricity to charge their phones. These factors were reported to be barriers to using the digital targeted client communication.	
**Assessment for each CERQual component**	
Methodological limitations	Minor concerns about methodological limitations due to poor reporting of sampling (unclear how participants were recruited in several studies) and researcher reflexivity
Coherence	No or very minor concerns about coherence
Relevance	No or very minor concerns about relevance
Adequacy	No or very minor concerns about adequacy
**Overall CERQual assessment**	
High confidence	Due to minor concerns regarding methodological limitations
**Contributing studies**	
**Study**	**Context**
Akinfaderin‐Agarau 2012	Nigeria; adolescent girls and young women; using mobile phones to provide sexual and reproductive health information and services; hypothetical with *no* examples of programme content
Cornelius 2009	USA; African‐American adolescents; SMS to support HIV/AIDS curriculum; hypothetical with *no* examples of programme content
Flax 2017	Nigeria; women of all ages belonging to microcredit financing groups; received weekly cell phone breastfeeding text and voice messages to a shared phone; part of an RCT or pilot RCT
Hirsch‐Moverman 2017	Lesotho; HIV patients; SMS to provide real‐time adherence support to people on HIV and TB treatment; part of an RCT or pilot RCT
Jalloh‐Vos 2014	Sierra Leone; pregnant and postpartum women and their partners; mobile phone intervention for antenatal care and family planning; part of an RCT or pilot RCT
Mbuagbaw 2012	Cameroon; HIV‐positive patients; SMS for HIV drug adherence; part of an RCT or pilot RCT
Mbuagbaw 2014	Cameroon; individuals living with HIV or involved in HIV support work; community‐owned text messaging programme to support people living with HIV; hypothetical with *no* examples of programme content
Smillie 2014	Canada; HIV‐positive people; SMS about HIV as part of the WelTel BC trial; pilot or implementation study with participation in an mHealth programme
Randomized control trial (RCT); Tuberculosis (TB)	

**Table:**

**Finding 6:** Participants reported varying degrees of access to mobile devices. For instance, some had no phone; some had lost or broken their phone; some could not afford to purchase airtime; some had changed their number or sim card; or for some access to the phone was controlled by another person. These factors were reported to be barriers to using the digital targeted client communication.	
**Assessment for each CERQual component**	
Methodological limitations	Minor concerns about methodological limitations due to poor reporting of sampling (unclear how participants were recruited in several studies) and researcher reflexivity
Coherence	No or very minor concerns about coherence
Relevance	Minor concerns about relevance due to a focus on study populations that may have limited access to mobile phone ownership, e.g. due to age, gender, socio‐economic status, or health condition (partial relevance)
Adequacy	No or very minor concerns about adequacy
**Overall CERQual assessment**	
Moderate confidence	Due to minor concerns regarding methodological limitations and relevance
**Contributing studies**	
**Study**	**Context**
Akinfaderin‐Agarau 2012	Nigeria; adolescent girls and young women; using mobile phones to provide sexual and reproductive health information and services; hypothetical with *no* examples of programme content
Entsieh 2015	Ghana; pregnant and nursing mothers aged 20 to 35; “Mobile Midwife” app; qualitative research on an existing programme implemented at scale
Flax 2017	Nigeria; Women of all ages belonging to micro credit financing groups; received weekly cell phone breastfeeding text and voice messages to a shared phone; Part of a RCT or pilot RCT
Hirsch‐Moverman 2017	Lesotho; HIV patients; SMS to provide real‐time adherence support to people on HIV and TB treatment; part of an RCT or pilot RCT
Jalloh‐Vos 2014	Sierra Leone; pregnant and postpartum women and their partners; mobile phone intervention for antenatal care and family planning; part of an RCT or pilot RCT
Jennings 2013	Kenya; HIV‐positive women enrolled in PMTCT and their male partners; SMS reminder for PMTCT testing; hypothetical with an example of messages being used
Menacho 2013	Peru; men who have sex with men; SMS to motivate for HIV testing; hypothetical with examples and with *no* examples of programme content
Missal 2016	India; husbands of pregnant women 12 to 20 weeks along; voice messages about antenatal care and preparing for delivery; pilot or implementation study with participation in an mHealth programme
Rana 2015	Uganda; HIV‐positive youth receiving ART; SMS for HIV‐positive youth; Hypothetical with *no* examples of programme content
Smillie 2014	Canada; HIV‐positive people; SMS about HIV as part of the WelTel BC trial; pilot or implementation study with participation in an mHealth programme
Prevention of mother‐to‐child transmission (PMTCT); Tuberculosis (TB)	

**Table:**

**Finding 7:** Some participants, particularly women and adolescents, had their access to phones controlled or restricted by others, especially if they had to share or borrow a phone. They noted that they would often have to explain why they wanted to use the phone, and who they wanted to call, to allay suspicions about this communication. They mentioned that this was a barrier to accessing digital targeted client communication and made it difficult to keep their messages private.	
**Assessment for each CERQual component**	
Methodological limitations	Minor concerns about methodological limitations due to poor reporting of sampling (unclear how participants were recruited in several studies) and researcher reflexivity
Coherence	Minor concerns about coherence, as the majority of participants in 1 study did not see phone sharing as a problem
Relevance	Minor concerns about relevance due to a focus on study populations that may have limited access to mobile phone ownership, e.g. due to age, gender, SES, or health condition (partial relevance)
Adequacy	Minor concerns about adequacy due to a limited number of studies
**Overall CERQual assessment**	
Moderate confidence	Due to minor concerns regarding methodological limitations, coherence, adequacy, and relevance
**Contributing studies**	
**Study**	**Context**
Akinfaderin‐Agarau 2012	Nigeria; adolescent girls and young women; using mobile phones to provide sexual and reproductive health information and services; hypothetical with *no* examples of programme content
Flax 2017	Nigeria; Women of all ages belonging to micro credit financing groups; received weekly cell phone breastfeeding text and voice messages to a shared phone; Part of a RCT or pilot RCT
Jalloh‐Vos 2014	Sierra Leone; pregnant and postpartum women and their partners; mobile phone intervention for antenatal care and family planning; part of an RCT or pilot RCT
Rana 2015	Uganda; HIV‐positive youth receiving ART; SMS for HIV‐positive youth; Hypothetical with *no* examples of programme content

**Table:**

**Finding 8:** Participants believed that the cost of taking part in digital targeted client communication should be free or very low, as cost could present a barrier to participation, particularly for young people and those on lower incomes. Participants felt that there should be little or no charge for costs such as joining the digital health intervention, downloading applications (apps), or for sending and receiving mobile messages/phone calls.	
**Assessment for each CERQual component**	
Methodological limitations	No or very minor concerns about methodological limitations
Coherence	No or very minor concerns about coherence
Relevance	Minor concerns about relevance due to partial relevance in relation to participant group (adolescents focus) and/or in low or middle‐income settings where cost may be particularly important
Adequacy	No or very minor concerns about adequacy
**Overall CERQual assessment**	
High confidence	Due to minor concerns regarding relevance
**Contributing studies**	
**Study**	**Context**
Akinfaderin‐Agarau 2012	Nigeria; adolescent girls and young women; using mobile phones to provide sexual and reproductive health information and services; hypothetical with *no* examples of programme content
Calderon 2017	Peru; women over 18 who had at least 1 child; SMS‐based mHealth programme on child health; hypothetical with *no* examples of programme content
Cornelius 2009	USA; African‐American adolescents; SMS to support HIV/AIDS curriculum; hypothetical with *no* examples of programme content
Menacho 2013	Peru; men who have sex with men; SMS to motivate for HIV testing; hypothetical with examples and with *no* examples of programme content
Mitchell 2016	USA; men who have sex with men; an app to motivate for HIV testing; hypothetical with *no* examples of programme content
Perry 2012	USA; adolescents aged 15 to 20; SMS with preventative sexual health messages; evaluation or formative research on an existing mHealth programme that the participants have been using
Rana 2015	Uganda; HIV‐positive youth receiving ART; SMS for HIV‐positive youth; Hypothetical with *no* examples of programme content
Smith 2017	Cambodia; women who had received an abortion; mobile phone voice messaging and counsellor support for postabortion care; part of an RCT or pilot RCT

**Table:**

**Finding 9:** Participants’ ability to access digital communication was sometimes limited by their language skills and their personal level of literacy or techno‐literacy, or both.	
**Assessment for each CERQual component**	
Methodological limitations	Moderate concerns about methodological limitations due to poor reporting of sampling, participant voices in the findings, and researcher reflexivity
Coherence	No or very minor concerns about coherence
Relevance	Minor concerns about relevance due to partial relevance of study population (populations that are more likely to have literacy and language challenges)
Adequacy	No or very minor concerns about adequacy
**Overall CERQual assessment**	
Moderate confidence	Due to minor concerns regarding relevance and moderate concerns regarding methodological limitations
**Contributing studies**	
**Study**	**Context**
Akinfaderin‐Agarau 2012	Nigeria; adolescent girls and young women; using mobile phones to provide sexual and reproductive health information and services; hypothetical with *no* examples of programme content
Calderon 2017	Peru; women over 18 who had at least 1 child; SMS‐based mHealth programme on child health; hypothetical with *no* examples of programme content
Curioso 2009	Peru; HIV‐positive adults receiving ART; SMS related to HIV/AIDS; hypothetical with *no* examples of programme content
Greaney 2014	USA; Latina women over the age of 21 needing cancer screening; interactive voice call reminding of screening; hypothetical with an example of messages being used
Hirsch‐Moverman 2017	Lesotho; HIV patients; SMS to provide real‐time adherence support to people on HIV and TB treatment; part of an RCT or pilot RCT
Jalloh‐Vos 2014	Sierra Leone; pregnant and postpartum women and their partners; mobile phone intervention for antenatal care and family planning; part of an RCT or pilot RCT
Mbuagbaw 2014	Cameroon; individuals living with HIV or involved in HIV support work; community‐owned text messaging programme to support people living with HIV; hypothetical with *no* examples of programme content
Rodrigues 2015	India; participants in the intervention arm of the trial; interactive voice recordings and SMS for HIV ART adherence; part of an RCT or pilot RCT
Smillie 2014	Canada; HIV‐positive people; SMS about HIV as part of the WelTel BC trial; pilot or implementation study with participation in an mHealth programme
Anti retroviral therapy (ART); Tuberculosis (TB)	

**Table:**

**Finding 10:** Participants often had preferences for how often health messages were sent, the time of day they were sent, and the duration of the digital targeted client communication. However, there was variation in what most participants felt was appropriate timing and frequency, and these preferences were often linked to the health issue on which the messaging was focused; whether people had their own phone or had to share a phone; and the participant’s particular circumstances. Participants were particularly concerned about being bombarded with too many messages; whether the timing of the messages was convenient for them; and/or whether messages arrived in connection with the behaviour the message was trying to target.	
**Assessment for each CERQual component**	
Methodological limitations	Minor concerns about methodological limitations due to poor reporting of researcher reflexivity
Coherence	No or very minor concerns about coherence
Relevance	Moderate concerns about relevance due to a fair number of studies where participants did not experience an mHealth intervention but were asked to comment about their preferences regarding a hypothetical intervention
Adequacy	No or very minor concerns about adequacy
**Overall CERQual assessment**	
Moderate confidence	Due to minor concerns regarding methodological limitations and moderate concerns regarding relevance
**Contributing studies**	
**Study**	**Context**
Calderon 2017	Peru; women over 18 who had at least 1 child; SMS‐based mHealth programme on child health; hypothetical with *no* examples of programme content
Cornelius 2009	USA; African‐American adolescents; SMS to support HIV/AIDS curriculum; hypothetical with *no* examples of programme content
Evans 2016	UK; African communities; SMS‐based HIV mHealth programme; hypothetical with examples and with *no* examples of programme content
French 2016	UK; young people aged 16 to 24; SMS on sexually transmitted infections; part of an RCT or pilot RCT
Gold 2010	Australia; young people aged 16 to 24; SMS on sexually transmitted infections; part of an RCT or pilot RCT
Greaney 2014	USA; Latina women over the age of 21 needing cancer screening; interactive voice call reminding of screening; hypothetical with an example of messages being used
Jennings 2013	Kenya; HIV‐positive women enrolled in PMTCT and their male partners; SMS reminder for PMTCT testing; hypothetical with an example of messages being used
Mbuagbaw 2012	Cameroon; HIV‐positive patients; SMS for HIV drug adherence; part of an RCT or pilot RCT
Menacho 2013	Peru; men who have sex with men; SMS to motivate for HIV testing; hypothetical with examples and with *no* examples of programme content
Missal 2016	India; husbands of pregnant women 12 to 20 weeks along; voice messages about antenatal care and preparing for delivery; pilot or implementation study with participation in an mHealth programme
Mitchell 2016	USA; men who have sex with men; an app to motivate for HIV testing; hypothetical with *no* examples of programme content
Munro 2017	Canada; pregnant or have given birth in the last 12 months; SMS Text4baby programme about prenatal education; hypothetical with an example of messages being used
Naughton 2013	UK; women who smoked during a recent pregnancy; SMS for smoking cessation during pregnancy; pilot or implementation study with participation in an mHealth programme
Odeny 2014	Kenya; women; SMS for early infant HIV testing; hypothetical with *no* examples of programme content
Rana 2015	Uganda; HIV‐positive youth receiving ART; SMS for HIV‐positive youth; Hypothetical with *no* examples of programme content
Rodrigues 2015	India; participants in the intervention arm of the trial; interactive voice recordings and SMS for HIV ART adherence; part of an RCT or pilot RCT
Sloan 2017	UK; women who had received the MiQuit intervention during pregnancy; SMS for smoking cessation during pregnancy; part of an RCT or pilot RCT
Smillie 2014	Canada; HIV‐positive people; SMS about HIV as part of the WelTel BC trial; pilot or implementation study with participation in an mHealth programme
Smith 2017	Cambodia; women who had received an abortion; mobile phone voice messaging and counsellor support for postabortion care; part of an RCT or pilot RCT
Ware 2016	Uganda; HIV‐positive patients initiating ART; SMS for ART adherence; part of an RCT or pilot RCT
Willoughby 2017	USA; college students; SMS for sexual health promotion; hypothetical with an example of messages being used
Wright 2011	USA; African‐American men aged 16 to 20; SMS for HIV prevention; hypothetical with an example of messages being used
Prevention of mother‐to‐child transmission (PMTCT); Randomized control trial (RCT)	

**Table:**

**Finding 11:** Participants had different preferences for various delivery channels available for sharing information through digital targeted client communication, including mobile messaging, interactive voice response, or speaking with a healthcare provider. These preferences were influenced by a number of factors including cost, convenience, the ability to store messages and re‐read them, familiarity with the channel, personal preferences, the nature of the content being delivered, the nature of the topic, language and literacy considerations, and the ability to have a discussion with a real‐life person.	
**Assessment for each CERQual component**	
Methodological limitations	Minor concerns about methodological limitations due to poor reporting of researcher reflexivity
Coherence	No or very minor concerns about coherence
Relevance	Moderate concerns about relevance due to a fair number of studies where participants did not experience an mHealth intervention but were asked to comment about their preferences regarding a hypothetical intervention. However, they may still have had experience with the communication channel outside of an mHealth programme that they could draw on.
Adequacy	No or very minor concerns about adequacy
**Overall CERQual assessment**	
Moderate confidence	Due to minor concerns regarding methodological limitations and moderate concerns regarding relevance
**Contributing studies**	
**Study**	**Context**
Akinfaderin‐Agarau 2012	Nigeria; adolescent girls and young women; using mobile phones to provide sexual and reproductive health information and services; hypothetical with *no* examples of programme content
Cates 2015	USA; middle school students designing text messages to promote HPV vaccine; hypothetical with an example of messages being used
Curioso 2009	Peru; HIV‐positive adults receiving ART; SMS related to HIV/AIDS; hypothetical with *no* examples of programme content
Greaney 2014	USA; Latina women over the age of 21 needing cancer screening; interactive voice call reminding of screening; hypothetical with an example of messages being used
Jennings 2013	Kenya; HIV‐positive women enrolled in PMTCT and their male partners; SMS reminder for PMTCT testing; hypothetical with an example of messages being used
Missal 2016	India; husbands of pregnant women 12 to 20 weeks along; voice messages about antenatal care and preparing for delivery; pilot or implementation study with participation in an mHealth programme
Mitchell 2016	USA; men who have sex with men; an app to motivate for HIV testing; hypothetical with *no* examples of programme content
Naughton 2013	UK; women who smoked during a recent pregnancy; SMS for smoking cessation during pregnancy; pilot or implementation study with participation in an mHealth programme
Odeny 2014	Kenya; women; SMS for early infant HIV testing; hypothetical with *no* examples of programme content
Rana 2015	Uganda; HIV‐positive youth receiving ART; SMS for HIV‐positive youth; Hypothetical with *no* examples of programme content
Rodrigues 2015	India; participants in the intervention arm of the trial; interactive voice recordings and SMS for HIV ART adherence; part of an RCT or pilot RCT
Smillie 2014	Canada; HIV‐positive people; SMS about HIV as part of the WelTel BC trial; pilot or implementation study with participation in an mHealth programme
Willoughby 2017	USA; college students; SMS for sexual health promotion; hypothetical with an example of messages being used
Anti retroviral therapy (ARV); Human Papillomavirus (HPV); Prevention of mother‐to‐child transmission (PMTCT)	

**Table:**

**Finding 12**: Participants appreciated personalised health information and discussed their preferences for options to make interventions more relevant to individuals. This could include sender‐based personalisation or receiver‐based options. Reasons for these preferences included engaging the user, enhancing credibility, increasing feelings of ownership, control over their personal information, and feelings of privacy. Preferences for tailoring included making digital health messages personalised by using an individual's name; allowing participants to choose the content, topic, and language of their messages; providing information relevant to the participant's setting (local information); allowing them to select the timing and frequency of the message; providing personalised reminders (e.g. for vaccination or medication); and allowing participants to have control over privacy settings.	
**Assessment for each CERQual component**	
Methodological limitations	Minor concerns about methodological limitations due to poor reporting of researcher reflexivity
Coherence	No or very minor concerns about coherence
Relevance	Serious concerns about relevance due to a large number of studies where participants did not experience an mHealth intervention but were asked to comment about their preferences regarding a hypothetical intervention
Adequacy	No or very minor concerns about adequacy
**Overall CERQual assessment**	
Low confidence	Due to minor concerns regarding methodological limitations and serious concerns regarding relevance
**Contributing studies**	
**Study**	**Context**
Calderon 2017	Peru; women over 18 who had at least 1 child; SMS‐based mHealth programme on child health; hypothetical with *no* examples of programme content
Evans 2016	UK; African communities; SMS‐based HIV mHealth programme; hypothetical with examples and with *no* examples of programme content
French 2016	UK; young people aged 16 to 24; SMS on sexually transmitted infections; part of an RCT or pilot RCT
Goldenberg 2015	USA; men who have sex with men; SMS on HIV testing reminders; hypothetical with examples and with *no* examples of programme content
Hirsch‐Moverman 2017	Lesotho; HIV patients; SMS to provide real‐time adherence support to people on HIV and TB treatment; part of an RCT or pilot RCT
Jennings 2013	Kenya; HIV‐positive women enrolled in PMTCT and their male partners; SMS reminder for PMTCT testing; hypothetical with an example of messages being used
Munro 2017	Canada; pregnant or have given birth in the last 12 months; SMS Text4baby programme about prenatal education; hypothetical with an example of messages being used
Naughton 2013	UK; women who smoked during a recent pregnancy; SMS for smoking cessation during pregnancy; pilot or implementation study with participation in an mHealth programme
Odeny 2014	Kenya; women; SMS for early infant HIV testing; hypothetical with *no* examples of programme content
Sloan 2017	UK; women who had received the MiQuit intervention during pregnancy; SMS for smoking cessation during pregnancy; part of an RCT or pilot RCT
Ware 2016	Uganda; HIV‐positive patients initiating ART; SMS for ART adherence; part of an RCT or pilot RCT
Willoughby 2017	USA; college students; SMS for sexual health promotion; hypothetical with an example of messages being used
Prevention of mother‐to‐child transmission (PMTCT); Randomized control trial (RCT); Tuberculosis (TB)	

**Table:**

**Finding 13:** Participants mentioned various message formats that they preferred. These included a preference for short, concise, personalised, clear, and direct messages in a language they could understand and in full text rather than "text speak".	
**Assessment for each CERQual component**	
Methodological limitations	Minor concerns about methodological limitations due to poor reporting of participant voices in the findings and researcher reflexivity
Coherence	No or very minor concerns about coherence
Relevance	Serious concerns about relevance due to partial relevance of study population (several of the studies were among adolescents) and a fair number of studies where participants did not experience an mHealth intervention but were asked to comment about their preferences regarding a hypothetical intervention
Adequacy	No or very minor concerns about adequacy
**Overall CERQual assessment**	
Low confidence	Due to minor concerns regarding methodological limitations and serious concerns regarding relevance
**Contributing studies**	
**Study**	**Context**
Akinfaderin‐Agarau 2012	Nigeria; adolescent girls and young women; using mobile phones to provide sexual and reproductive health information and services; hypothetical with *no* examples of programme content
Calderon 2017	Peru; women over 18 who had at least 1 child; SMS‐based mHealth programme on child health; hypothetical with *no* examples of programme content
Cates 2015	USA; middle school students designing text messages to promote HPV vaccine; hypothetical with an example of messages being used
Curioso 2009	Peru; HIV‐positive adults receiving ART; SMS related to HIV/AIDS; hypothetical with *no* examples of programme content
Evans 2016	UK; African communities; SMS‐based HIV mHealth programme; hypothetical with examples and with *no* examples of programme content
French 2016	UK; young people aged 16 to 24; SMS on sexually transmitted infections; part of an RCT or pilot RCT
Gold 2010	Australia; young people aged 16 to 24; SMS on sexually transmitted infections; part of an RCT or pilot RCT
Greaney 2014	USA; Latina women over the age of 21 needing cancer screening; interactive voice call reminding of screening; hypothetical with an example of messages being used
Lau 2014	South Africa; pregnant women; SMS for antenatal health promotion; part of an RCT or pilot RCT
Menacho 2013	Peru; men who have sex with men; SMS to motivate for HIV testing; hypothetical with examples and with *no* examples of programme content
Missal 2016	India; husbands of pregnant women 12 to 20 weeks along; voice messages about antenatal care and preparing for delivery; pilot or implementation study with participation in an mHealth programme
Munro 2017	Canada; pregnant or have given birth in the last 12 months; SMS Text4baby programme about prenatal education; hypothetical with an example of messages being used
Naughton 2013	UK; women who smoked during a recent pregnancy; SMS for smoking cessation during pregnancy; pilot or implementation study with participation in an mHealth programme
Odeny 2014	Kenya; women; SMS for early infant HIV testing; hypothetical with *no* examples of programme content
Perry 2012	USA; adolescents aged 15 to 20; SMS with preventative sexual health messages; evaluation or formative research on an existing mHealth programme that the participants have been using
Rana 2015	Uganda; HIV‐positive youth receiving ART; SMS for HIV‐positive youth; Hypothetical with *no* examples of programme content
Smillie 2014	Canada; HIV‐positive people; SMS about HIV as part of the WelTel BC trial; pilot or implementation study with participation in an mHealth programme
Willoughby 2017	USA; college students; SMS for sexual health promotion; hypothetical with an example of messages being used
Anti retroviral therapy (ART); Human Papiloma Virus (HPV); Randomized control trial (RCT)	

**Table:**

**Finding 14:** Participants’ perceptions of who sent the digital health communication could influence their trust in and perception of the credibility and value of the digital targeted client communication and the information it provides. Participants said they wanted a known, identified phone number; messages sent from a reliable, trusted, credible source such as health professionals or official sources; and in some cases to feel like the messages were sent by a person (even if sent from an automated service). However, some participants, such as those with stigmatised health conditions, preferred an unmarked sender to protect their privacy.	
**Assessment for each CERQual component**	
Methodological limitations	Minor concerns about methodological limitations due to poor reporting of participant voices in the findings and researcher reflexivity
Coherence	No or very minor concerns about coherence
Relevance	Moderate concerns about relevance due to a fair number of studies where participants did not experience an mHealth intervention but were asked to comment about their preferences regarding a hypothetical intervention
Adequacy	No or very minor concerns about adequacy
**Overall CERQual assessment**	
Moderate confidence	Due to minor concerns regarding methodological limitations and moderate concerns regarding relevance
**Contributing studies**	
**Study**	**Context**
Akinfaderin‐Agarau 2012	Nigeria; adolescent girls and young women; using mobile phones to provide sexual and reproductive health information and services; hypothetical with *no* examples of programme content
Brown 2014	USA; single, adolescent mothers; health promotion information weekly via SMS during the first 6 months postpartum; pilot or implementation study with participation in an mHealth programme
Calderon 2017	Peru; women over 18 who had at least 1 child; SMS‐based mHealth programme on child health; hypothetical with *no* examples of programme content
Cates 2015	USA; middle school students designing text messages to promote HPV vaccine; hypothetical with an example of messages being used
Evans 2016	UK; African communities; SMS‐based HIV mHealth programme; hypothetical with examples and with *no* examples of programme content
Greaney 2014	USA; Latina women over the age of 21 needing cancer screening; interactive voice call reminding of screening; hypothetical with an example of messages being used
Lau 2014	South Africa; pregnant women; SMS for antenatal health promotion; part of an RCT or pilot RCT
Mbuagbaw 2012	Cameroon; HIV‐positive patients; SMS for HIV drug adherence; part of an RCT or pilot RCT
Menacho 2013	Peru; men who have sex with men; SMS to motivate for HIV testing; hypothetical with examples and with *no* examples of programme content
Missal 2016	India; husbands of pregnant women 12 to 20 weeks along; voice messages about antenatal care and preparing for delivery; pilot or implementation study with participation in an mHealth programme
Naughton 2013	UK; women who smoked during a recent pregnancy; SMS for smoking cessation during pregnancy; pilot or implementation study with participation in an mHealth programme
Rana 2015	Uganda; HIV‐positive youth receiving ART; SMS for HIV‐positive youth; Hypothetical with *no* examples of programme content
Rodrigues 2015	India; participants in the intervention arm of the trial; interactive voice recordings and SMS for HIV ART adherence; part of an RCT or pilot RCT
Smillie 2014	Canada; HIV‐positive people; SMS about HIV as part of the WelTel BC trial; pilot or implementation study with participation in an mHealth programme
Willoughby 2017	USA; college students; SMS for sexual health promotion; hypothetical with an example of messages being used
Human Papiloma Virus (HPV)	

**Table:**

**Finding 15:** Participants said that the tone of digital health communication mattered to them. Their preferences varied but included a tone that was: motivational, friendly, encouraging, polite, respectful, congratulatory, personalised, upbeat, positive, humorous, and relatable. Some participants highlighted that they did not like feeling pressured, lectured to, shamed, or frightened by digital health messages.	
**Assessment for each CERQual component**	
Methodological limitations	Minor concerns about methodological limitations due to poor reporting of researcher reflexivity
Coherence	No or very minor concerns about coherence
Relevance	Serious concerns about relevance due to partial relevance of study population (several of the studies were among adolescents) and a fair number of studies where participants did not experience an mHealth intervention but were asked to comment about their preferences regarding a hypothetical intervention
Adequacy	No or very minor concerns about adequacy
**Overall CERQual assessment**	
Low confidence	Due to minor concerns regarding methodological limitations and serious concerns regarding relevance
**Contributing studies**	
**Study**	**Context**
Cates 2015	USA; middle school students designing text messages to promote HPV vaccine; hypothetical with an example of messages being used
Curioso 2009	Peru; HIV‐positive adults receiving ART; SMS related to HIV/AIDS; hypothetical with *no* examples of programme content
Evans 2016	UK; African communities; SMS‐based HIV mHealth programme; hypothetical with examples and with *no* examples of programme content
French 2016	UK; young people aged 16 to 24; SMS on sexually transmitted infections; part of an RCT or pilot RCT
Gold 2010	Australia; young people aged 16 to 24; SMS on sexually transmitted infections; part of an RCT or pilot RCT
Jennings 2013	Kenya; HIV‐positive women enrolled in PMTCT and their male partners; SMS reminder for PMTCT testing; hypothetical with an example of messages being used
Menacho 2013	Peru; men who have sex with men; SMS to motivate for HIV testing; hypothetical with examples and with *no* examples of programme content
Munro 2017	Canada; pregnant or have given birth in the last 12 months; SMS Text4baby programme about prenatal education; hypothetical with an example of messages being used
Naughton 2013	UK; women who smoked during a recent pregnancy; SMS for smoking cessation during pregnancy; pilot or implementation study with participation in an mHealth programme
Odeny 2014	Kenya; women; SMS for early infant HIV testing; hypothetical with *no* examples of programme content
Perry 2012	USA; adolescents aged 15 to 20; SMS with preventative sexual health messages; evaluation or formative research on an existing mHealth programme that the participants have been using
Rana 2015	Uganda; HIV‐positive youth receiving ART; SMS for HIV‐positive youth; Hypothetical with *no* examples of programme content
Sloan 2017	UK; women who had received the MiQuit intervention during pregnancy; SMS for smoking cessation during pregnancy; part of an RCT or pilot RCT
Wright 2011	USA; African‐American men aged 16 to 20; SMS for HIV prevention; hypothetical with an example of messages being used
Anti retroviral therapy (ARV); Human Papillomavirus (HPV); Prevention of mother‐to‐child transmission (PMTCT); Randomized control trial (RCT)	

**Table:**

**Finding 16:** Participants had preferences regarding the content they receive through digital targeted client communication. They wanted varied content that provided new knowledge and reminders, as well as explanations, solutions, and suggestions about health issues. They were interested in content related to health, illness, and treatments and practical topics such as health facility location and transportation. They wanted this information to be relevant and acceptable to their personal circumstances and local setting.	
**Assessment for each CERQual component**	
Methodological limitations	Minor concerns about methodological limitations due to poor reporting of researcher reflexivity
Coherence	No or very minor concerns about coherence
Relevance	Moderate concerns about relevance due to a fair number of studies where participants did not experience an mHealth intervention but were asked to comment about their preferences regarding a hypothetical intervention
Adequacy	No or very minor concerns about adequacy
**Overall CERQual assessment**	
Moderate confidence	Due to minor concerns regarding methodological limitations and moderate concerns regarding relevance
**Contributing studies**	
**Study**	**Context**
Brown 2014	USA; single, adolescent mothers; health promotion information weekly via SMS during the first 6 months postpartum; pilot or implementation study with participation in an mHealth programme
Calderon 2017	Peru; women over 18 who had at least 1 child; SMS‐based mHealth programme on child health; hypothetical with *no* examples of programme content
Cornelius 2009	USA; African‐American adolescents; SMS to support HIV/AIDS curriculum; hypothetical with *no* examples of programme content
Entsieh 2015	Ghana; pregnant and nursing mothers aged 20 to 35; “Mobile Midwife” app; qualitative research on an existing programme implemented at scale
French 2016	UK; young people aged 16 to 24; SMS on sexually transmitted infections; part of an RCT or pilot RCT
Gold 2010	Australia; young people aged 16 to 24; SMS on sexually transmitted infections; part of an RCT or pilot RCT
Greaney 2014	USA; Latina women over the age of 21 needing cancer screening; interactive voice call reminding of screening; hypothetical with an example of messages being used
Jalloh‐Vos 2014	Sierra Leone; pregnant and postpartum women and their partners; mobile phone intervention for antenatal care and family planning; part of an RCT or pilot RCT
Jennings 2013	Kenya; HIV‐positive women enrolled in PMTCT and their male partners; SMS reminder for PMTCT testing; hypothetical with an example of messages being used
Mbuagbaw 2014	Cameroon; individuals living with HIV or involved in HIV support work; community‐owned text messaging programme to support people living with HIV; hypothetical with *no* examples of programme content
Missal 2016	India; husbands of pregnant women 12 to 20 weeks along; voice messages about antenatal care and preparing for delivery; pilot or implementation study with participation in an mHealth programme
Mitchell 2016	USA; men who have sex with men; an app to motivate for HIV testing; hypothetical with *no* examples of programme content
Munro 2017	Canada; pregnant or have given birth in the last 12 months; SMS Text4baby programme about prenatal education; hypothetical with an example of messages being used
Nachega 2016	South Africa; HIV‐infected pregnant women; SMS about ART adherence to prevent PMTCT; hypothetical with *no* examples of programme content
Odeny 2014	Kenya; women; SMS for early infant HIV testing; hypothetical with *no* examples of programme content
Perry 2012	USA; adolescents aged 15 to 20; SMS with preventative sexual health messages; evaluation or formative research on an existing mHealth programme that the participants have been using
Sloan 2017	UK; women who had received the MiQuit intervention during pregnancy; SMS for smoking cessation during pregnancy; part of an RCT or pilot RCT
Smith 2017	Cambodia; women who had received an abortion; mobile phone voice messaging and counsellor support for postabortion care; part of an RCT or pilot RCT
Anti retroviral therapy (ARV); Prevention of mother‐to‐child transmission (PMTCT); Randomized control trial (RCT)	

**Table:**

**Finding 17:** Some participants felt that including elements in the mobile‐based platform in which participants are asked for a response (e.g. via knowledge quizzes or multiple‐choice questions or a practical tool allowing access to additional information, such as a nutrition calculator) could increase the engagement of users with the intervention, its content, and provide additional information to them. In one study, participants suggested that it would be helpful if the response was quick, simple, and convenient.	
**Assessment for each CERQual component**	
Methodological limitations	Minor concerns about methodological limitations, as most studies were fairly well conducted and reported (the lack of reflexivity in 3 of the studies is not a serious concern because of the focus of the finding)
Coherence	No or very minor concerns about coherence
Relevance	Serious concerns about relevance due to a fair number of studies where participants did not experience an mHealth intervention but were asked to comment about their preferences regarding a hypothetical intervention; all of the studies were conducted in high‐income countries, and most of the studies were on adolescent and youth populations
Adequacy	Moderate concerns about adequacy due to the small number of studies and thin data
**Overall CERQual assessment**	
Low confidence	Due to minor concerns regarding methodological limitations, moderate concerns regarding adequacy, and serious concerns regarding relevance
**Contributing studies**	
**Study**	**Context**
Cornelius 2009	USA; African‐American adolescents; SMS to support HIV/AIDS curriculum; hypothetical with *no* examples of programme content
Munro 2017	Canada; pregnant or have given birth in the last 12 months; SMS Text4baby programme about prenatal education; hypothetical with an example of messages being used
Naughton 2013	UK; women who smoked during a recent pregnancy; SMS for smoking cessation during pregnancy; pilot or implementation study with participation in an mHealth programme
Wright 2011	USA; African‐American men aged 16 to 20; SMS for HIV prevention; hypothetical with an example of messages being used

**Table:**

**Finding 18:** Some participants with health issues that are often seen as stigmatised or very personal (e.g. HIV, family planning, and abortion care) worried that their confidential health information would be disclosed or their identity traced due to their participation in digital targeted client communication. In general, people’s perceptions of information delivery channels (SMS, interactive voice response, voice call) were influenced by how confidential they felt the delivery channels to be.	
**Assessment for each CERQual component**	
Methodological limitations	Minor concerns about methodological limitations due to poor reporting of researcher reflexivity
Coherence	No or very minor concerns about coherence
Relevance	No or very minor concerns about relevance
Adequacy	No or very minor concerns about adequacy
**Overall CERQual assessment**	
High confidence	Due to minor concerns regarding methodological limitations
**Contributing studies**	
**Study**	**Context**
Akinfaderin‐Agarau 2012	Nigeria; adolescent girls and young women; using mobile phones to provide sexual and reproductive health information and services; hypothetical with *no* examples of programme content
Calderon 2017	Peru; women over 18 who had at least 1 child; SMS‐based mHealth programme on child health; hypothetical with *no* examples of programme content
Cates 2015	USA; middle school students designing text messages to promote HPV vaccine; hypothetical with an example of messages being used
Curioso 2009	Peru; HIV‐positive adults receiving ART; SMS related to HIV/AIDS; hypothetical with *no* examples of programme content
Evans 2016	UK; African communities; SMS‐based HIV mHealth programme; hypothetical with examples and with *no* examples of programme content
French 2016	UK; young people aged 16 to 24; SMS on sexually transmitted infections; part of an RCT or pilot RCT
Goldenberg 2015	USA; men who have sex with men; SMS on HIV testing reminders; hypothetical with examples and with *no* examples of programme content
Greaney 2014	USA; Latina women over the age of 21 needing cancer screening; interactive voice call reminding of screening; hypothetical with an example of messages being used
Jalloh‐Vos 2014	Sierra Leone; pregnant and postpartum women and their partners; mobile phone intervention for antenatal care and family planning; part of an RCT or pilot RCT
Jennings 2013	Kenya; HIV‐positive women enrolled in PMTCT and their male partners; SMS reminder for PMTCT testing; hypothetical with an example of messages being used
Mbuagbaw 2012	Cameroon; HIV‐positive patients; SMS for HIV drug adherence; part of an RCT or pilot RCT
Mbuagbaw 2014	Cameroon; individuals living with HIV or involved in HIV support work; community‐owned text messaging programme to support people living with HIV; hypothetical with *no* examples of programme content
Menacho 2013	Peru; men who have sex with men; SMS to motivate for HIV testing; hypothetical with examples and with *no* examples of programme content
Mitchell 2016	USA; men who have sex with men; an app to motivate for HIV testing; hypothetical with *no* examples of programme content
Nachega 2016	South Africa; HIV‐infected pregnant women; SMS about ART adherence to prevent PMTCT; hypothetical with *no* examples of programme content
Odeny 2014	Kenya; women; SMS for early infant HIV testing; hypothetical with *no* examples of programme content
Perry 2012	USA; adolescents aged 15 to 20; SMS with preventative sexual health messages; evaluation or formative research on an existing mHealth programme that the participants have been using
Rana 2015	Uganda; HIV‐positive youth receiving ART; SMS for HIV‐positive youth; Hypothetical with *no* examples of programme content
Rodrigues 2015	India; participants in the intervention arm of the trial; interactive voice recordings and SMS for HIV ART adherence; part of an RCT or pilot RCT
Smith 2017	Cambodia; women who had received an abortion; mobile phone voice messaging and counsellor support for postabortion care; part of an RCT or pilot RCT
Willoughby 2017	USA; college students; SMS for sexual health promotion; hypothetical with an example of messages being used
Anti retroviral therapy (ARV); Human Papillomavirus (HPV); Prevention of mother‐to‐child transmission (PMTCT); Randomized control trial (RCT)	

**Table:**

**Finding 19:** Some participants proposed strategies to address their concerns regarding confidentiality and privacy. These strategies for communication included neutral, coded, or discreet language; access codes; communication that does not disclose the sender; coming from a trusted sender; and the ability to tailor and control content, timing, and frequency of their messages.	
**Assessment for each CERQual component**	
Methodological limitations	Minor concerns about methodological limitations due to poor reporting of participant voices in the findings and of researcher reflexivity
Coherence	No or very minor concerns about coherence
Relevance	No or very minor concerns about relevance
Adequacy	No or very minor concerns about adequacy
**Overall CERQual assessment**	
High confidence	Due to minor concerns regarding methodological limitations
**Contributing studies**	
**Study**	**Context**
Calderon 2017	Peru; women over 18 who had at least 1 child; SMS‐based mHealth programme on child health; hypothetical with *no* examples of programme content
Curioso 2009	Peru; HIV‐positive adults receiving ART; SMS related to HIV/AIDS; hypothetical with *no* examples of programme content
Evans 2016	UK; African communities; SMS‐based HIV mHealth programme; hypothetical with examples and with *no* examples of programme content
French 2016	UK; young people aged 16 to 24; SMS on sexually transmitted infections; part of an RCT or pilot RCT
Goldenberg 2015	USA; men who have sex with men; SMS on HIV testing reminders; hypothetical with examples and with *no* examples of programme content
Greaney 2014	USA; Latina women over the age of 21 needing cancer screening; interactive voice call reminding of screening; hypothetical with an example of messages being used
Mbuagbaw 2012	Cameroon; HIV‐positive patients; SMS for HIV drug adherence; part of an RCT or pilot RCT
Menacho 2013	Peru; men who have sex with men; SMS to motivate for HIV testing; hypothetical with examples and with *no* examples of programme content
Odeny 2014	Kenya; women; SMS for early infant HIV testing; hypothetical with *no* examples of programme content
Rana 2015	Uganda; HIV‐positive youth receiving ART; SMS for HIV‐positive youth; Hypothetical with *no* examples of programme content
Rodrigues 2015	India; participants in the intervention arm of the trial; interactive voice recordings and SMS for HIV ART adherence; part of an RCT or pilot RCT
Smith 2017	Cambodia; women who had received an abortion; mobile phone voice messaging and counsellor support for postabortion care; part of an RCT or pilot RCT
Willoughby 2017	USA; college students; SMS for sexual health promotion; hypothetical with an example of messages being used
Anti retroviral therapy (ARV); Randomized control trial (RCT)	

**Table:**

**Finding 20:** Some participants thought that participating in digital targeted client communication had influenced their behaviour, whilst others did not. Reasons given for the changes in behaviour included receiving new knowledge; receiving strategies on how to initiate discussion with a partner or healthcare provider; being motivated or reassured by the intervention; and being reminded, for example, to take medication or make an appointment. Some participants who believed that the intervention did not have any influence on their behaviour found that the digital health interventions were not relevant to them.	
**Assessment for each CERQual component**	
Methodological limitations	Moderate concerns about methodological limitations due to poor reporting of participant voice in the findings, ethical considerations, and researcher reflexivity
Coherence	No or very minor concerns about coherence
Relevance	Minor concerns about relevance due to the fact that a large group of the studies were tied to pregnancy and childbirth, which can in itself influence behaviour change
Adequacy	Minor concerns about adequacy due to thin data in some studies
**Overall CERQual assessment**	
Low confidence	Due to minor concerns regarding relevance and adequacy and moderate concerns regarding methodological limitations
**Contributing studies**	
**Study**	**Context**
Brown 2014	USA; single, adolescent mothers; health promotion information weekly via SMS during the first 6 months postpartum; pilot or implementation study with participation in an mHealth programme
Entsieh 2015	Ghana; pregnant and nursing mothers aged 20 to 35; “Mobile Midwife” app; qualitative research on an existing programme implemented at scale
French 2016	UK; young people aged 16 to 24; SMS on sexually transmitted infections; part of an RCT or pilot RCT
Gold 2010	Australia; young people aged 16 to 24; SMS on sexually transmitted infections; part of an RCT or pilot RCT
Greaney 2014	USA; Latina women over the age of 21 needing cancer screening; interactive voice call reminding of screening; hypothetical with an example of messages being used
Hirsch‐Moverman 2017	Lesotho; HIV patients; SMS to provide real‐time adherence support to people on HIV and TB treatment; part of an RCT or pilot RCT
Jalloh‐Vos 2014	Sierra Leone; pregnant and postpartum women and their partners; mobile phone intervention for antenatal care and family planning; part of an RCT or pilot RCT
Jennings 2013	Kenya; HIV‐positive women enrolled in PMTCT and their male partners; SMS reminder for PMTCT testing; hypothetical with an example of messages being used
Lau 2014	South Africa; pregnant women; SMS for antenatal health promotion; part of an RCT or pilot RCT
Missal 2016	India; husbands of pregnant women 12 to 20 weeks along; voice messages about antenatal care and preparing for delivery; pilot or implementation study with participation in an mHealth programme
Munro 2017	Canada; pregnant or have given birth in the last 12 months; SMS Text4baby programme about prenatal education; hypothetical with an example of messages being used
Rodrigues 2015	India; participants in the intervention arm of the trial; interactive voice recordings and SMS for HIV ART adherence; part of an RCT or pilot RCT
Sloan 2017	UK; women who had received the MiQuit intervention during pregnancy; SMS for smoking cessation during pregnancy; part of an RCT or pilot RCT
Smillie 2014	Canada; HIV‐positive people; SMS about HIV as part of the WelTel BC trial; pilot or implementation study with participation in an mHealth programme
Smith 2017	Cambodia; women who had received an abortion; mobile phone voice messaging and counsellor support for postabortion care; part of an RCT or pilot RCT
Ware 2016	Uganda; HIV‐positive patients initiating ART; SMS for ART adherence; part of an RCT or pilot RCT
Prevention of mother‐to‐child transmission (PMTCT); Randomized control trial (RCT); Tuberculosis (TB)	

**Table:**

**Finding 21:** Some participants suggested that the effects of the messaging may not be sustained over time, as they and others would become bored with or fatigued by the messages, especially if the content was not varied enough.	
**Assessment for each CERQual component**	
Methodological limitations	No or very minor concerns about methodological limitations
Coherence	No or very minor concerns about coherence
Relevance	Moderate concerns due to the fact that in the majority of studies participants did not experience an mHealth intervention but were asked to comment about their preferences regarding a hypothetical intervention, and a focus on HIV/AIDS (partial relevance)
Adequacy	Moderate concerns due to thin data in some of the included studies
**Overall CERQual assessment**	
Low confidence	Due to moderate concerns regarding relevance and adequacy
**Contributing studies**	
**Study**	**Context**
Cornelius 2009	USA; African‐American adolescents; SMS to support HIV/AIDS curriculum; hypothetical with *no* examples of programme content
Curioso 2009	Peru; HIV‐positive adults receiving ART; SMS related to HIV/AIDS; hypothetical with *no* examples of programme content
Evans 2016	UK; African communities; SMS‐based HIV mHealth programme; hypothetical with examples and with *no* examples of programme content
Gold 2010	Australia; young people aged 16 to 24; SMS on sexually transmitted infections; part of an RCT or pilot RCT
Menacho 2013	Peru; men who have sex with men; SMS to motivate for HIV testing; hypothetical with examples and with *no* examples of programme content
Mitchell 2016	USA; men who have sex with men; an app to motivate for HIV testing; hypothetical with *no* examples of programme content
Rana 2015	Uganda; HIV‐positive youth receiving ART; SMS for HIV‐positive youth; Hypothetical with *no* examples of programme content
Willoughby 2017	USA; college students; SMS for sexual health promotion; hypothetical with an example of messages being used
Anti retroviral therapy (ART)	

**Table:**

**Finding 22:** Some participants were concerned about becoming over‐reliant on digital reminders and thought that this might make them dependent on digital targeted communication for undertaking some health tasks. They were concerned that in the absence of these reminders they would adhere poorly to care plans.	
**Assessment for each CERQual component**	
Methodological limitations	Minor concerns about methodological limitations due to poor reporting of methods in 1 study
Coherence	No or very minor concerns about coherence
Relevance	Moderate concerns about relevance due to the fact that all of the studies are from 1 region; 2 focus on 1 health issue (HIV); and 1 is hypothetical
Adequacy	Serious concerns about adequacy due to thin data from a small number of studies
**Overall CERQual assessment**	
Low confidence	Due to minor concerns regarding methodological limitations, moderate concerns about relevance, and serious concerns about adequacy
**Contributing studies**	
**Study**	**Context**
Jalloh‐Vos 2014	Sierra Leone; pregnant and postpartum women and their partners; mobile phone intervention for antenatal care and family planning; part of an RCT or pilot RCT
Mbuagbaw 2012	Cameroon; HIV‐positive patients; SMS for HIV drug adherence; part of an RCT or pilot RCT
Rana 2015	Uganda; HIV‐positive youth receiving ART; SMS for HIV‐positive youth; Hypothetical with *no* examples of programme content
Anti retroviral therapy (ART)	

**Table:**

**Finding 23:** Some participants felt that digital health interventions could save them time and money by giving them access to health care via their mobile phones. This was especially relevant to participants who faced barriers in attending health care because of distance to a health facility and a lack of time and/or financial means.	
**Assessment for each CERQual component**	
Methodological limitations	Minor concerns about methodological limitations due to poor reporting of participant voices in the findings, researcher reflexivity, and unclear ethical considerations in 1 study
Coherence	No or very minor concerns about coherence
Relevance	Moderate concerns about relevance due to partial relevance of setting and populations who may be particularly affected by lack of time and funds, and distance
Adequacy	Moderate concerns about adequacy due to a limited number of studies
**Overall CERQual assessment**	
Low confidence	Due to minor concerns regarding methodological limitations and moderate concerns regarding adequacy and relevance
**Contributing studies**	
**Study**	**Context**
Calderon 2017	Peru; women over 18 who had at least 1 child; SMS‐based mHealth programme on child health; hypothetical with *no* examples of programme content
Smith 2017	Cambodia; women who had received an abortion; mobile phone voice messaging and counsellor support for postabortion care; part of an RCT or pilot RCT

**Table:**

**Finding 24:** Some participants felt that digital health interventions provided them with feelings of support and connectedness, as they felt that someone was taking the time to send them messages. A few participants felt that in some cases the sense of caring and support that they received from healthcare providers through digital health interventions had a positive influence on their relationship with their healthcare provider.	
**Assessment for each CERQual component**	
Methodological limitations	Moderate concerns about methodological limitations due to poor reporting of participant voice in the findings, ethical considerations, and researcher reflexivity
Coherence	No or very minor concerns about coherence
Relevance	Moderate concerns about relevance due to a fair number of studies where participants did not experience an mHealth intervention but were asked to comment about their preferences regarding a hypothetical intervention
Adequacy	No or very minor concerns about adequacy
**Overall CERQual assessment**	
Moderate confidence	Due to moderate concerns regarding methodological limitations and relevance
**Contributing studies**	
**Study**	**Context**
Brown 2014	USA; single, adolescent mothers; health promotion information weekly via SMS during the first 6 months postpartum; pilot or implementation study with participation in an mHealth programme
Calderon 2017	Peru; women over 18 who had at least 1 child; SMS‐based mHealth programme on child health; hypothetical with *no* examples of programme content
Entsieh 2015	Ghana; pregnant and nursing mothers aged 20 to 35; “Mobile Midwife” app; qualitative research on an existing programme implemented at scale
Jalloh‐Vos 2014	Sierra Leone; pregnant and postpartum women and their partners; mobile phone intervention for antenatal care and family planning; part of an RCT or pilot RCT
Lau 2014	South Africa; pregnant women; SMS for antenatal health promotion; part of an RCT or pilot RCT
Mbuagbaw 2014	Cameroon; individuals living with HIV or involved in HIV support work; community‐owned text messaging programme to support people living with HIV; hypothetical with *no* examples of programme content
Munro 2017	Canada; pregnant or have given birth in the last 12 months; SMS Text4baby programme about prenatal education; hypothetical with an example of messages being used
Nachega 2016	South Africa; HIV‐infected pregnant women; SMS about ART adherence to prevent PMTCT; hypothetical with *no* examples of programme content
Rana 2015	Uganda; HIV‐positive youth receiving ART; SMS for HIV‐positive youth; Hypothetical with *no* examples of programme content
Rodrigues 2015	India; participants in the intervention arm of the trial; interactive voice recordings and SMS for HIV ART adherence; part of an RCT or pilot RCT
Sloan 2017	UK; women who had received the MiQuit intervention during pregnancy; SMS for smoking cessation during pregnancy; part of an RCT or pilot RCT
Smillie 2014	Canada; HIV‐positive people; SMS about HIV as part of the WelTel BC trial; pilot or implementation study with participation in an mHealth programme
Smith 2017	Cambodia; women who had received an abortion; mobile phone voice messaging and counsellor support for postabortion care; part of an RCT or pilot RCT
Ware 2016	Uganda; HIV‐positive patients initiating ART; SMS for ART adherence; part of an RCT or pilot RCT
Wright 2011	USA; African‐American men aged 16 to 20; SMS for HIV prevention; hypothetical with an example of messages being used
Anti retroviral therapy (ART); Prevention of mother‐to‐child transmission (PMTCT)	

**Table:**

**Finding 25:** Participants described how they shared digital communication content more broadly with friends, family, and community members. Many participants felt that the information would be useful to others.	
**Assessment for each CERQual component**	
Methodological limitations	Minor concerns about methodological limitations due to poor reporting of participant voices in the findings and researcher reflexivity
Coherence	No or very minor concerns about coherence
Relevance	Moderate concerns about relevance due to the fact that in a fair number of studies participants did not experience an mHealth intervention but were asked to comment about their preferences regarding a hypothetical intervention
Adequacy	No or very minor concerns about adequacy
**Overall CERQual assessment**	
Moderate confidence	Due to minor concerns regarding methodological limitations and moderate concerns regarding relevance
**Contributing studies**	
**Study**	**Context**
Calderon 2017	Peru; women over 18 who had at least 1 child; SMS‐based mHealth programme on child health; hypothetical with *no* examples of programme content
Cornelius 2009	USA; African‐American adolescents; SMS to support HIV/AIDS curriculum; hypothetical with *no* examples of programme content
Flax 2017	Nigeria; women of all ages belonging to microcredit financing groups; received weekly cell phone breastfeeding text and voice messages to a shared phone; part of an RCT or pilot RCT
French 2016	UK; young people aged 16 to 24; SMS on sexually transmitted infections; part of an RCT or pilot RCT
Gold 2010	Australia; young people aged 16 to 24; SMS on sexually transmitted infections; part of an RCT or pilot RCT
Jennings 2013	Kenya; HIV‐positive women enrolled in PMTCT and their male partners; SMS reminder for PMTCT testing; hypothetical with an example of messages being used
Perry 2012	USA; adolescents aged 15 to 20; SMS with preventative sexual health messages; evaluation or formative research on an existing mHealth programme that the participants have been using
Smith 2017	Cambodia; women who had received an abortion; mobile phone voice messaging and counsellor support for postabortion care; part of an RCT or pilot RCT
Wright 2011	USA; African‐American men aged 16 to 20; SMS for HIV prevention; hypothetical with an example of messages being used
Prevention of mother‐to‐child transmission (PMTCT); Randomized control trial (RCT)	

### Appendix 5. Expanded matrix table

**Table:**

Have the trialists described efforts to address situations where members of the target group: 1. do not own a functioning mobile device; 2. have poor access to network services; 3. have poor access to electricity to charge mobile devices; 4. want to avoid expenses associated with the intervention, such as paying for airtime; 5. change their phone numbers or sim cards; 6. have access to the phone controlled by someone else; 7. have low literacy, differing language skills, or limited techno‐literacy; 8. have concerns about privacy and confidentiality; 9. perceive different sources as more or less reliable, trusted, and credible; 10. have members of the target group been given an opportunity to offer feedback about their needs, preferences, and experiences regarding the intervention.											
**Adolescents (N = 13)**											
**Trial**		1	2	3	4	5	6	7	8	9	10
1	Belzer 2015 (USA)	Y			Y			N			Y
2	Bull 2016 (USA)	Y	Y	Y	Y	Y	Y		Y	Y	Y
3	Castano 2012 (USA)	N			Y	?		Y	Y		N
4	Delamere 2006 (Ireland)										
5	Garofalo 2016 (USA)	N			Y	Y		Y	Y		Y
6	Gold 2011 (Australia)	N	Y		Y						Y
7	Jeffries 2016 (USA)	N						N			?
8	Lim 2012 (Australia)	N				N		N			Y
9	McCarthy 2016 (UK)	N				N		N	Y		Y
10	Reed 2014 (USA)	N							Y		
11	Rokicki 2017 (Ghana)	Y			?		?	?			Y
12	Suffoletto 2013 (USA)	N				?		N			?
13	Ybarra 2017 (USA)	N			N	N	N	?	Y		?
**Adults (N = 27)**											
**Trial**		1	2	3	4	5	6	7	8	9	10
14	Abdul 2013 (Malaysia)					N					
15	Barnabas 2016 (South Africa/Uganda)	N			Y			Y	Y		Y
16	Constant 2014 (South Africa)	N			Y		N	Y	?		Y
17	Cook 2015 (USA)	Y			Y			N			Y
18	de Costa 2012 (Brazil)	N			Y	N		N	Y		Y
19	de Tolly 2012 (South Africa)	N			Y			N			Y
20	Downing 2013 (Australia)	N				N					
21	Gerdts 2015 (Colombia)										
22	Hou 2010 (USA)	N	Y					?	Y		
23	Huang 2013 (China)	N							Y		
24	Ingersoll 2015 (USA)	Y	Y		Y			Y	Y		Y
25	Joseph 2016 (Mozambique)	N	N	N				N	Y		Y
26	Lee 2016 (USA)	Y						Y			Y
27	Leiby 2016 (Zambia)	N			Y						Y
28	Lester 2010 (Kenya)	Y			Y		Y	Y			Y
29	Mbuagbaw 2012 (Cameroon)	N			N			?	Y	Y	Y
30	Mugo 2016 (Kenya)	Y					Y	?	Y		
31	Norton 2014 (USA)	N			N	N			N		
32	Nsagha 2016 (Cameroon)	N						?			?
33	Odeny 2012 (Kenya)	N			Y	N		?			Y
34	Pop‐Eleches 2011 (Kenya)	Y		Y	Y			Y			Y
35	Ruan 2017 (China)	N				N		N	Y		Y
36	Russell 2012 (USA)										
37	Rutland 2012 (UK)										
38	Shet 2014 (India)	Y	N		Y			Y			Y
39	Smith 2015 (Cambodia)	N			N	N		Y	?	?	Y
40	Young 2015 (Peru)		?		Y				Y		Y
**Pregnant and postpartum women (N = 11)**											
**Trial**		1	2	3	4	5	6	7	8	9	10
41	Cooper 2015 (England)	N			Y			?		Y	Y
42	Evans 2014 (USA)	N						N			Y
43	Jareethum 2008 (Thailand)	N						?			
44	Joshi 2015 (India)	Y				Y		Y			Y
45	Kamau‐Mbuthia 2013 (Kenya)										
46	Lund 2012 (Zanzibar)	N	Y	N	Y			N			N
47	Maslowsky 2016 (Ecuador)				Y	Y		Y			Y
48	McConnell 2016 (Kenya)	Y				Y		?			
49	Moniz 2013 (USA)	N									?
50	Omole 2018 (Nigeria)	N			Y			N			?
51	Yudin 2017 (Canada)	N						N			Y
**Parents (N = 14)**											
**Trial**		1	2	3	4	5	6	7	8	9	10
52	Ahlers‐Schmidt 2012a (USA)	N				N					Y
23	Bangure 2015 (Zimbabwe)	N				?					Y
54	Bigna 2015 (Cameroon)	N						N	?		Y
55	Brown 2016 (Nigeria)	Y									Y
56	Domek 2016 (Guatemala)	N						Y			Y
57	Eze 2015 (Nigeria)	N				N		Y			
58	Gibson 2017 (Kenya)	Y									Y
59	Haji 2016 (Kenya)	N						Y			
60	Hannan 2016 (USA)										
61	Hofstetter 2015 (USA)	N				N		Y			
62	Jimenez 2017 (USA)							?			Y
63	Niederhauser 2015 (USA)	N						N			
64	Sharma 2011 (India)	N						N			
65	Stockwell 2014 (USA)	N						Y			
**Mothers living with HIV/AIDS (N = 3)**											
**Trial**		1	2	3	4	5	6	7	8	9	10
66	Kassaye 2016 (Kenya)	Y			Y		?	Y	?		Y
67	Kebaya 2015 (Kenya)										
68	Odeny 2014 (Kenya)	Y			Y			Y	Y		Y
N = No; Y = Yes; Blank = Not mentioned; ? = Unclear											

### Appendix 6. List of all studies included in the matrix analysis

#### Adolescents (N = 13)

**Belzer 2015**

- Belzer ME, Kolmodin M, Clark LF, Huang J, Olson J, Kahana SY, et al. Acceptability and feasibility of a cell phone support intervention for youth living with HIV with nonadherence to antiretroviral therapy. AIDS Patient Care & STDs 2015;29(6):338‐45.
- Belzer ME, Naar‐King S, Olson J, Sarr M, Thornton S, Kahana SY, et al. The use of cell phone support for non‐adherent HIV‐infected youth and young adults: an initial randomized and controlled intervention trial. AIDS and Behavior 2014;18(4):686‐96.

**Bull 2016**

- Bull S, Devine S, Schmiege SJ, Pickard L, Campbell J, Shlay JC, et al. Text messaging, teen outreach program, and sexual health behavior: a cluster randomized trial. American Journal of Public Health 2016;106(S1):S117‐24.
- Bull S, Devine S, Schmiege SJ, Hammes A, Pickard L, Shlay JC. Text messaging and teen sexual health behavior: long‐term follow‐up of a cluster randomized trial. Computers, Informatics, Nursing 2017;35(11):549‐53.
- Devine S, Bull S, Dreisbach S, Shlay J. Enhancing a teen pregnancy prevention program with text messaging: engaging minority youth to develop TOP® Plus Text. Journal of Adolescent Health 2014;54(3):S78‐83.
- Devine S, Leeds C, Shlay JC, Leytem A, Beum R, Bull S. Methods to assess youth engagement in a text messaging supplement to an effective teen pregnancy program. Journal of Biomedical Informatics 2015;56:379‐86.
- Bull S, Devine S, Schmiege SJ, Pickard L, Campbell J, Shlay JC. Text messaging, teen outreach program, and sexual health behavior: a cluster randomized trial. American Journal of Public Health 2016;106(S1):S117‐24.

**Castano 2012**

- Castano PM, Bynum JY, Andres R, Lara M, Westhoff C. Effect of daily text messages on oral contraceptive continuation: a randomized controlled trial. Obstetrics & Gynecology 2012;119(1):14‐20.
- Hall K, Castano P, Westhoff C. Oral contraceptive knowledge modestly associated with oral contraceptive continuation among young, urban women. Contraception 2011;84(3):320‐1.
- Hall KS, Castano PM, Westhoff CL. The influence of oral contraceptive knowledge on oral contraceptive continuation among young women. Journal of Women's Health 2014;23(7):596‐601.
- Hall KS, Westhoff CL, Castano PM. The impact of an educational text message intervention on young urban women's knowledge of oral contraception. Contraception 2013;87(4):449‐54.

**Delamere 2006**

- Delamere S, Dooley S, Harrington L, King A, Mulcahy F. Safer sex text messages: evaluating a health education intervention in an adolescent population. Sexually Transmitted Infections 2006;82:A27.

**Garofalo 2016**

- Garofalo R, Kuhns LM, Hotton A, Johnson A, Muldoon A, Rice D. A randomized controlled trial of personalized text message reminders to promote medication adherence among HIV‐positive adolescents and young adults. AIDS and Behavior 2016;20(5):1049‐59.
- Dowshen N, Kuhns LM, Gray C, Lee S, Garofalo R. Feasibility of interactive text message response (ITR) as a novel, real‐time measure of adherence to antiretroviral therapy for HIV+ youth. AIDS and Behavior 2013;17(6):2237‐43.
- Dowshen N, Kuhns LM, Johnson A, Holoyda BJ, Garofalo R. Improving adherence to antiretroviral therapy for youth living with HIV/AIDS: a pilot study using personalized, interactive, daily text message reminders. Journal of Medical Internet Research 2012;14(2):e51.

**Gold 2011**

- Gold J, Aitken CK, Dixon HG, Lim MS, Gouillou M, Spelman T, et al. A randomised controlled trial using mobile advertising to promote safer sex and sun safety to young people. Health Education Research 2011;26(5):782‐94.

**Jeffries 2016**

- Jeffries C, Ross P, Matoff‐Stepp S, Thompson R, Harris JL, Uhrig JD, et al. Ucare4life: mobile texting to improve HIV care continuum outcomes for minority youth. Topics in antiviral medicine 2016;24:427.

**Lim 2012**

- Lim MS, Hocking JS, Aitken CK, Fairley CK, Jordan L, Lewis JA, et al. Impact of text and email messaging on the sexual health of young people: a randomised controlled trial. Journal of Epidemiology & Community Health 2012;66(1):69‐74.

**McCarthy 2016**

- Free C, McCarthy O, French RS, Wellings K, Michie S, Roberts I. Can text messages increase safer sex behaviours in young people? Intervention development and pilot randomised controlled trial. Health Technology Assessment 2016;20(57):1‐82.
- McCarthy OL, French RS, Baraitser P, Roberts I, Rathod SD, Devries K, et al. Safetxt: a pilot randomised controlled trial of an intervention delivered by mobile phone to increase safer sex behaviours in young people. BMJ Open 2016;6(12):e013045.
- French RS, McCarthy O, Baraitser P, Wellings K, Bailey JV, Free C. Young people’s views and experiences of a mobile phone texting intervention to promote safer sex behavior. JMIR mHealth and uHealth 2016;4(2):e26.

**Reed 2014**

- Reed JL, Huppert JS, Taylor RG, Gillespie GL, Byczkowski TL, Kahn JA, et al. Improving sexually transmitted infection results notification via mobile phone technology. Journal of Adolescent Health 2014;55(5):690‐7.

**Rokicki 2017**

- Rokicki S, Cohen J, Salomon JA, Fink G. Impact of a text‐messaging program on adolescent reproductive health: a cluster‐randomized trial in Ghana. American Journal of Public Health 2017;107(2):298‐305.
- Rokicki S, Fink G. Assessing the reach and effectiveness of mHealth: evidence from a reproductive health program for adolescent girls in Ghana. BMC Public Health 2017;17(1):969.

**Suffoletto 2013**

- Suffoletto B, Akers A, McGinnis KA, Calabria J, Wiesenfeld HC, Clark DB. A sex risk reduction text‐message program for young adult females discharged from the emergency department. Journal of Adolescent Health 2013;53(3):387‐93.

**Ybarra 2017**

- Ybarra ML, Prescott TL, Phillips GL Jr, Bull SS, Parsons JT, Mustanski B. Pilot RCT results of an mHealth HIV prevention program for sexual minority male adolescents. Pediatrics 2017;140(1):e20162999.
- Prescott TL, Phillips G Jr, DuBois LZ, Bull SS, Mustanski B, Ybarra ML. Reaching adolescent gay, bisexual, and queer men online: development and refinement of a national recruitment strategy. Journal of Medical Internet Research 2016;18(8):e200.
- Ybarra ML, Prescott TL, Philips GL, Bull SS, Parsons JT, Mustanski B. Iteratively developing an mHealth HIV prevention program for sexual minority adolescent men. AIDS and Behavior 2016;20(6):1157‐72.
- Ybarra ML, Prescott TL, Phillips GL Jr, Parsons JT, Bull SS, Mustanski B. Ethical considerations in recruiting online and implementing a text messaging–based HIV prevention program with gay, bisexual, and queer adolescent males. Journal of Adolescent Health 2016;59(1):44‐9.

#### Adults (N = 27)

**Abdul Rashid 2013**

- Abdul Rashid RM, Mohamed M, Hamid ZA, Dahlui M. Is the phone call the most effective method for recall in cervical cancer screening? Results from a randomised control trial. Asian Pacific Journal of Cancer Prevention 2013;14(10):5901‐4.
- Rashid RM, Ramli S, John J, Dahlui M. Cost effective analysis of recall methods for cervical cancer screening in Selangor ‐ results from a prospective randomized controlled trial. Asian Pacific Journal of Cancer Prevention 2014;15(13):5143‐7.
- Rashid RMA, Dahlui M. Cost effective analysis of different types of recall on patients' response rate in a pap smear screening program. Journal of Health and Translational Medicine 2013;16:73.

**Barnabas 2016**

- Barnabas RV, van R, Tumwesigye E, Brantley J, Baeten JM, van H, et al. Uptake of antiretroviral therapy and male circumcision after community‐based HIV testing and strategies for linkage to care versus standard clinic referral: a multisite, open‐label, randomised controlled trial in South Africa and Uganda. Lancet HIV 2016;3(5):e212‐20.
- Gilbert HN, Wyatt MA, Asiimwe S, Turyamureeba B, Tumwesigye E, Van Rooyen H, et al. Messaging circumstances and economic pressures as influences on linkage to medical male circumcision following community‐based HIV testing for men in rural southwest Uganda: a qualitative study. AIDS Research and Treatment 2018;2018:Article ID 8387436.
- Ware NC, Wyatt MA, Asiimwe S, Turyamureeba B, Tumwesigye E, Van Rooyen H, et al. How home HIV testing and counselling with follow‐up support achieves high testing coverage and linkage to treatment and prevention: a qualitative analysis from Uganda. Journal of the International AIDS Society 2016;19(1):20929.

**Constant 2014**

- Constant D, de Tolly K, Harries J, Myer L. Mobile phone messages to provide support to women during the home phase of medical abortion in South Africa: a randomised controlled trial. Contraception 2014;90(3):226‐33.
- de Tolly K, Constant D. Integrating mobile phones into medical abortion provision: intervention development, use, and lessons learned from a randomized controlled trial. JMIR mHealth and uHealth 2014;2(1):e5.
- Constant D, de Tolly K, Harries J, Myer L. Assessment of completion of early medical abortion using a text questionnaire on mobile phones compared to a self‐administered paper questionnaire among women attending four clinics, Cape Town, South Africa. Reproductive Health Matters 2014;22(Suppl 44):83‐93.

**Cook 2015**

- Cook PF, Carrington JM, Schmiege SJ, Starr W, Reeder B. A counselor in your pocket: feasibility of mobile health tailored messages to support HIV medication adherence. Patient Preference & Adherence 2015;9:1353‐66.

**da Costa 2012**

- da Costa TM, Barbosa BJ, Gomes e Costa DA, Sigulem D, de Fátima Marin H, Filho AC, et al. Results of a randomized controlled trial to assess the effects of a mobile SMS‐based intervention on treatment adherence in HIV/AIDS‐infected Brazilian women and impressions and satisfaction with respect to incoming messages. International Journal of Medical Informatics 2012;81(4):257‐69.

**de Tolly 2012**

- de Tolly K, Skinner D, Nembaware V, Benjamin P. Investigation into the use of short message services to expand uptake of human immunodeficiency virus testing, and whether content and dosage have impact. Telemedicine Journal and e‐Health 2012;18(1):18‐23.

**Downing 2013**

- Downing SG, Cashman C, McNamee H, Penney D, Russell DB, Hellard ME. Increasing chlamydia test of re‐infection rates using SMS reminders and incentives. Sexually Transmitted Infections 2013;89(1):16‐9.

**Gerdts 2015**

- Gerdts C, Moseson H, Mora M, DePineres T. Alternative follow‐up options for medical abortion in Colombia: a pilot randomized controlled trial testing the feasibility of text‐messages. Contraception 2015;92(4):373.

**Hou 2010**

- Hou MY, Hurwitz S, Kavanagh E, Fortin J, Goldberg AB. Using daily text‐message reminders to improve adherence with oral contraceptives: a randomized controlled trial. Obstetrics and Gynecology 2010;116(3):633‐40.

**Huang 2013**

- Huang D, Sangthong R, McNeil E, Chongsuvivatwong V, Zheng W, Yang X. Effects of a phone call intervention to promote adherence to antiretroviral therapy and quality of life of HIV/AIDS patients in Baoshan, China: a randomized controlled trial. AIDS Research & Treatment 2013;2013:580974.

**Ingersoll 2015**

- Ingersoll KS, Dillingham RA, Hettema JE, Conaway M, Freeman J, Reynolds G, et al. Pilot RCT of bidirectional text messaging for ART adherence among nonurban substance users with HIV. Health Psychology 2015;34S:1305‐15.

**Joseph Davey 2016**

- Joseph Davey D, Nhavoto JA, Augusto O, Ponce W, Traca D, Nguimfack A, et al. SMSaude: Evaluating mobile phone text reminders to improve retention in HIV care for patients on antiretroviral therapy in Mozambique. Journal of Acquired Immune Deficiency Syndromes 2016;73(2):e23‐30.

**Lee 2016**

- Lee HY, Le C, Ghebre R, Yee D. Mobile phone multimedia messaging intervention for breast cancer screening. Cancer Research 2016;76(4):Abstract P3‐08‐03.
- Lee H, Ghebre R, Le C, Jang YJ, Sharratt M, Yee D. Mobile phone multilevel and multimedia messaging intervention for breast cancer screening: pilot randomized controlled trial. JMIR mHealth and uHealth 2017;5(11):e154.
- Lee H, Lee M, Gao Z, Sadak K. Development and evaluation of culturally and linguistically tailored mobile app to promote breast cancer screening. Journal of Clinical Medicine 2018;7(8):181.

**Leiby 2016**

- Leiby K, Connor A, Tsague L, Sapele C, Kaonga A, Kakaire J, et al. The impact of SMS‐based interventions on VMMC uptake in Lusaka Province, Zambia: a randomized controlled trial. Journal of Acquired Immune Deficiency Syndromes 2016;72(Suppl 4):S264‐72.

**Lester 2010**

- Chi BH, Stringer JS. Mobile phones to improve HIV treatment adherence. Lancet 2010;376(9755):1807‐8.
- Lester RT, Ritvo P, Mills EJ, Kariri A, Karanja S, Chung MH, et al. Effects of a mobile phone short message service on antiretroviral treatment adherence in Kenya (WelTel Kenya1): a randomised trial. Lancet 2010;376(9755):1838‐45.
- Memetovic J, Kop ML, Karanja S, Kimani J, Ngugi EN, Ritvo P, et al. Perceived stigma and disclosure of HIV status: their association with clinical and communication outcomes in the WelTel Kenya1 SMS randomized controlled trial. Canadian Journal of Infectious Diseases and Medical Microbiology 2013;24:107a.
- Lester RT, Mills EJ, Kariri A, Ritvo P, Chung M, Jack W, et al. The HAART cell phone adherence trial (WelTel Kenya1): a randomized controlled trial protocol. Trials 2009;10(1):87.
- Smillie K, Borek NV, Kop MLvd, Lukhwaro A, Li N, Karanja S, et al. Mobile health for early retention in HIV care: a qualitative study in Kenya (WelTel Retain). African Journal of AIDS Research 2014;13(4):331‐8.

**Mbuagbaw 2012**

- Mbuagbaw L, Thabane L, Ongolo‐Zogo P, Lester RT, Mills EJ, Smieja M, et al. The Cameroon Mobile Phone SMS (CAMPS) trial: a randomized trial of text messaging versus usual care for adherence to antiretroviral therapy. PLOS ONE 2012;7(12):e46909.
- Mbuagbaw L, Bonono‐Momnougui RC, Thabane L. Considerations in using text messages to improve adherence to highly active antiretroviral therapy: a qualitative study among clients in Yaounde, Cameroon. HIV/AIDS (Auckland, NZ) 2012;4:45.
- Mbuagbaw L, Thabane L, Ongolo‐Zogo P. Opening communication channels with people living with HIV using mobile phone text messaging: insights from the CAMPS trial. BMC Research Notes 2013;6(1):131.
- Mbuagbaw L, Thabane L, Ongolo‐Zogo P, Lang T. The challenges and opportunities of conducting a clinical trial in a low resource setting: the case of the Cameroon mobile phone SMS (CAMPS) trial, an investigator initiated trial. Trials 2011;12(1):145.
- Mbuagbaw L, Thabane L, Ongolo‐Zogo P, Lester RT, Mills E, Volmink J, et al. The Cameroon mobile phone SMS (CAMPS) trial: a protocol for a randomized controlled trial of mobile phone text messaging versus usual care for improving adherence to highly active anti‐retroviral therapy. Trials 2011;12(1):5.

**Mugo 2016**

- Mugo PM, Wahome EW, Gichuru E, Mwashigadi G, Thiong'o AN, Prins HA, et al. Effect of SMS, phone‐call, and in‐person reminders on repeat HIV test uptake in Kenya. Topics in antiviral medicine 2016;24:420.
- Mugo PM, Wahome EW, Gichuru EN, Mwashigadi GM, Thiong'o AN, Prins HA, et al. Effect of text message, phone call, and in‐person appointment reminders on uptake of repeat HIV testing among outpatients screened for acute HIV infection in Kenya: a randomized controlled trial. PLOS ONE 2016;11(4):e0153612.

**Norton 2014**

- Norton BL, Person AK, Castillo C, Pastrana C, Subramanian M, Stout JE. Barriers to using text message appointment reminders in an HIV clinic. Telemedicine Journal and e‐health 2014;20(1):86‐9.

**Nsagha 2016**

- Nsagha DS, Lange I, Fon PN, Nguedia Assob JC, Tanue EA. A randomized controlled trial on the usefulness of mobile text phone messages to improve the quality of care of HIV and AIDS patients in Cameroon. Open AIDS Journal 2016;10:93‐103.

**Odeny 2012**

- Odeny TA, Bailey RC, Bukusi EA, Simoni JM, Tapia KA, Yuhas K, et al. Effect of text messaging to deter early resumption of sexual activity after male circumcision for HIV prevention: a randomized controlled trial. Journal of Acquired Immune Deficiency Syndromes 2014;65(2):e50‐7.
- Odeny TA, Bailey RC, Bukusi EA, Simoni JM, Tapia KA, Yuhas K, et al. Text messaging to improve attendance at post‐operative clinic visits after adult male circumcision for HIV prevention: a randomized controlled trial. PLOS ONE 2012;7(9):e43832.

**Pop‐Eleches 2011**

- Pop‐Eleches C, Thirumurthy H, Habyarimana JP, Zivin JG, Goldstein MP, de Walque D, et al. Mobile phone technologies improve adherence to antiretroviral treatment in a resource‐limited setting: a randomized controlled trial of text message reminders. AIDS 2011;25(6):825‐34.

**Rutland 2012**

- Rutland E, Roe H, Weaver A. Health promotional messages in short message service (SMS) follow‐up of GU medicine clinic defaulters; a tool to improve subsequent attendance rates? Sexually Transmitted Infections 2012;88(Suppl 1):A4.

**Shet 2014**

- De Costa A, Shet A, Kumarasamy N, Ashorn P, Eriksson B, Bogg L, et al. Design of a randomized trial to evaluate the influence of mobile phone reminders on adherence to first line antiretroviral treatment in South India ‐ the HIVIND study protocol. BMC Medical Research Methodolgy 2010;10:25.
- Erratum: Effect of mobile telephone reminders on treatment outcome in HIV: evidence from a randomised controlled trial in India. BMJ (Online) 2014;349(g5978):no pagination.
- Rodrigues R, Poongulali S, Balaji K, Atkins S, Ashorn P, De Costa A. 'The phone reminder is important, but will others get to know about my illness?' Patient perceptions of an mHealth antiretroviral treatment support intervention in the HIVIND trial in South India. BMJ Open 2015;5(11):e007574.
- Shet A, De Costa A, Kumarasamy N, Rodrigues R, Rewari BB, Ashorn P, et al. Effect of mobile telephone reminders on treatment outcome in HIV: evidence from a randomised controlled trial in India. BMJ 2014;349:g5978.
- Rodrigues R, Shet A, Antony J, Sidney K, Arumugam K, Krishnamurthy S, et al. Supporting adherence to antiretroviral therapy with mobile phone reminders: results from a cohort in South India. PLOS ONE 2012;7(8):e40723.
- Shet A, Arumugam K, Rodrigues R, Rajagopalan N, Shubha K, Raj T, et al. Designing a mobile phone‐based intervention to promote adherence to antiretroviral therapy in South India. AIDS and Behavior 2010;14(3):716‐20.
- Shet A, De Costa A, Kumarasamy N, Rodrigues R, Rewari BB, Ashorn P, et al. Effect of mobile telephone reminders on treatment outcome in HIV: evidence from a randomised controlled trial in India. BMJ 2014;349:g5978.
- Rodrigues R, Poongulali S, Balaji K, Atkins S, Ashorn P, De C. 'The phone reminder is important, but will others get to know about my illness?' Patient perceptions of an mHealth antiretroviral treatment support intervention in the HIVIND trial in South India. BMJ Open 2015;5(11):e007574.

**Smith 2015**

- Smith C, Ngo TD, Gold J, Edwards P, Vannak U, Sokhey L, et al. Effect of a mobile phone‐based intervention on post‐abortion contraception: a randomized controlled trial in Cambodia. Bulletin of the World Health Organization 2015;93(12):842‐50A.
- Smith C, Edwards P, Free C. Assessing the validity and reliability of self‐report data on contraception use in the MObile Technology for Improved Family Planning (MOTIF) randomised controlled trial. Reproductive Health 2018;15(1):50.
- Smith C, Jarvis C, Free C. Assessing loss to follow‐up in the MObile Technology for Improved Family Planning (MOTIF) randomised controlled trial. Trials 2017;18(1):577.
- Smith C, Ly S, Uk V, Warnock R, Edwards P, Free C. Process evaluation of a mobile phone‐based intervention to support post‐abortion contraception in Cambodia. Contraception and Reproductive Medicine 2017;2(1):16.
- Smith C, Ly S, Uk V, Warnock R, Free C. Women’s views and experiences of a mobile phone‐based intervention to support post‐abortion contraception in Cambodia. Reproductive Health 2017;14(1):72.
- Smith C, Ngo TD, Edwards P, Free C. MObile Technology for Improved Family Planning: update to randomised controlled trial protocol. Trials 2014;15(1):440.
- Smith C, Vannak U, Sokhey L, Ngo TD, Gold J, Free C. Mobile Technology for Improved Family Planning (MOTIF): the development of a mobile phone‐based (mHealth) intervention to support post‐abortion family planning (PAFP) in Cambodia. Reproductive Health 2015;13(1):1.
- Smith C, Vannak U, Sokhey L, Ngo TD, Gold J, Khut K, et al. MObile Technology for Improved Family Planning services (MOTIF): study protocol for a randomised controlled trial. Trials 2013;14(1):427.

**Young 2015**

- Young SD, Cumberland WG, Nianogo R, Menacho LA, Galea JT, Coates T. The HOPE social media intervention for global HIV prevention in Peru: a cluster randomised controlled trial. Lancet HIV 2015;2(1):e27‐32.
- Chiu CJ, Menacho L, Fisher C, Young SD. Ethics issues in social media–based HIV prevention in low‐ and middle‐income countries. Cambridge Quarterly of Healthcare Ethics 2015;24(3):303‐10.
- Garett R, Menacho L, Young SD. Ethical issues in using social media to deliver an HIV prevention intervention: results from the HOPE Peru Study. Prevention Science 2017;18(2):225‐32.
- Jaganath D, Gill HK, Cohen AC, Young SD. Harnessing Online Peer Education (HOPE): integrating C‐POL and social media to train peer leaders in HIV prevention. AIDS Care 2012;24(5):593‐600.
- Krueger EA, Chiu CJ, Menacho LA, Young SD. HIV testing among social media‐using Peruvian men who have sex with men: correlates and social context. AIDS Care 2016;28(10):1301‐5.
- Young SD, Holloway I, Jaganath D, Rice E, Westmoreland D, Coates T. Project HOPE: online social network changes in an HIV prevention randomized controlled trial for African American and Latino men who have sex with men. American Journal of Public Health 2014;104(9):1707‐12.
- Young SD, Jaganath D. Online social networking for HIV education and prevention: a mixed methods analysis. Sexually Transmitted Diseases 2013;40(2).
- Menacho LA, Blas MM, Alva IE, Orellana ER. Short text messages to motivate HIV testing among men who have sex with men: a qualitative study in Lima, Peru. Open AIDS Journal 2013;7:1.

#### Pregnant and postpartum women (N = 11)

**Cooper 2015**

- Cooper S, Foster K, Naughton F, Leonardi‐Bee J, Sutton S, Ussher M, et al. Pilot study to evaluate a tailored text message intervention for pregnant smokers (MiQuit): study protocol for a randomised controlled trial. Trials 2015;16:29.
- Naughton F, Cooper S, Foster K, Emery J, Leonardi‐Bee J, Sutton S, et al. Large multi‐centre pilot randomized controlled trial testing a low‐cost, tailored, self‐help smoking cessation text message intervention for pregnant smokers (MiQuit). Addiction 2017;112(7):1238‐49.
- Emery JL, Coleman T, Sutton S, Cooper S, Leonardi‐Bee J, Jones M, et al. Uptake of tailored text message smoking cessation support in pregnancy when advertised on the internet (MiQuit): observational study. Journal of Medical Internet Research 2018;20(4):e146.
- Naughton F, Cooper S, Bowker K, Campbell K, Sutton S, Leonardi‐Bee J, et al. Adaptation and uptake evaluation of an SMS text message smoking cessation programme (MiQuit) for use in antenatal care. BMJ Open 2015;5(10):e008871.
- Naughton F, Prevost AT, Gilbert H, Sutton S. Randomized controlled trial evaluation of a tailored leaflet and SMS text message self‐help intervention for pregnant smokers (MiQuit). Nicotine & Tobacco Research 2012;14(5):569‐77.
- Sloan M, Hopewell S, Coleman T, Cooper S, Naughton F. Smoking cessation support by text message during pregnancy: a qualitative study of views and experiences of the MiQuit intervention. Nicotine & Tobacco Research 2017;19(5):572‐7.

**Evans 2014**

- Evans WD, Wallace B, Szekely D, Nielsen P, Murray E, Abroms L, et al. Initial outcomes from a 4‐week follow‐up study of the Text4baby program in the military women's population: randomized controlled trial. Journal of Medical Internet Research 2014;16(5):e131.
- Evans W, Nielsen PE, Szekely DR, Bihm JW, Murray EA, Snider J, et al. Dose‐response effects of the text4baby mobile health program: randomized controlled trial. JMIR mHealth and uHealth 2015;3(1):e12.
- Evans WD, Abroms LC, Poropatich R, Nielsen PE, Wallace JL. Mobile health evaluation methods: the Text4baby case study. Journal of Health Communication 2012;17(Suppl 1):22‐9.
- Gazmararian JA, Elon L, Yang B, Graham M, Parker R. Text4baby program: an opportunity to reach underserved pregnant and postpartum women? Maternal and Child Health Journal 2014;18(1):223‐32.

**Jareethum 2008**

- Jareethum R, Titapant V, Chantra T, Sommai V, Chuenwattana P, Jirawan C. Satisfaction of healthy pregnant women receiving short message service via mobile phone for prenatal support: a randomized controlled trial. Chotmaihet Thangphaet [Journal of the Medical Association of Thailand] 2008;91(4):458‐63.

**Joshi 2015**

- Joshi S, Patil N, Hegde A. Impact of mHealth initiative on utilization of antenatal care services in rural Maharashtra, India. Indian Journal of Maternal and Child Health 2015;17(2):1‐7.

**Kamau‐Mbuthia 2013**

- Kamau‐Mbuthia E, Mbugua S, Webb G, Kalungu S, Sarange C, Lou W, et al. Cell phone based peer counseling to support exclusive breastfeeding is associated with more frequent help and decreased breastfeeding problems. Annals of Nutrition & Metabolism 2013;63:196‐7.
- Webb G, Kamau‐Mbuthia E, Mbugua S, Kalungu S, Sarange C, Lou W, et al. Infant medication, illness and growth in a randomized controlled trial of exclusive breastfeeding support in Kenya. Annals of Nutrition & Metabolism 2013;63:752.

**Lund 2012**

- Lund S, Hemed M, Nielsen BB, Said A, Said K, Makungu MH, et al. Mobile phones as a health communication tool to improve skilled attendance at delivery in Zanzibar: a cluster‐randomised controlled trial. BJOG: An International Journal of Obstetrics & Gynaecology 2012;119(10):1256‐64.
- Lund S, Nielsen BB, Hemed M, Boas IM, Said A, Said K, et al. Mobile phones improve antenatal care attendance in Zanzibar: a cluster randomized controlled trial. BMC Pregnancy & Childbirth 2014;14:29.
- Lund S, Nielsen BB, Hemed M, Said A, Said K, Makungu MH, et al. Mobile phones as a health communication tool to improve maternal and perinatal health in Zanzibar: a cluster randomised controlled trial. Tropical Medicine & International Health 2013;18:22.
- Lund S, Rasch V, Hemed M, Boas IM, Said A, Said K, et al. Mobile phone intervention reduces perinatal mortality in Zanzibar: secondary outcomes of a cluster randomized controlled trial. JMIR mHealth and uHealth 2014;2(1):e15.

**Maslowsky 2016**

- Maslowsky J, Frost S, Hendrick CE, Trujillo Cruz FO, Merajver SD. Effects of postpartum mobile phone‐based education on maternal and infant health in Ecuador. International Journal of Gynaecology & Obstetrics 2016;134(1):93‐8.

**McConnell 2016**

- McConnell M, Ettenger A, Rothschild CW, Muigai F, Cohen J. Can a community health worker administered postnatal checklist increase health‐seeking behaviors and knowledge?: Evidence from a randomized trial with a private maternity facility in Kiambu County, Kenya. BMC Pregnancy Childbirth 2016;16(1):136.

**Moniz 2013**

- Moniz MH, Hasley S, Meyn LA, Beigi RH. Improving influenza vaccination rates in pregnancy through text messaging: a randomized controlled trial. Obstetrics & Gynecology 2013;121(4):734‐40.

**Omole 2018**

- Omole O, Ijadunola MY, Olotu E, Omotoso O, Bello B, Awoniran O, et al. The effect of mobile phone short message service on maternal health in south‐west Nigeria. International Journal of Health Planning and Management 2018;33(1):155‐70.

**Yudin 2017**

- Yudin MH, Mistry N, De Souza LR, Besel K, Patel V, Blanco Mejia S, et al. Text messages for influenza vaccination among pregnant women: a randomized controlled trial. Vaccine 2017;35(5):842‐8.

#### Parents (N = 14)

**Ahlers‐Schmidt 2012a**

- Ahlers‐Schmidt CR, Chesser AK, Nguyen T, Brannon J, Hart TA, Williams KS, et al. Feasibility of a randomized controlled trial to evaluate Text Reminders for Immunization Compliance in Kids (TRICKs). Vaccine 2012;30(36):5305‐9.

**Bangure 2015**

- Bangure D, Chirundu D, Gombe N, Marufu T, Mandozana G, Tshimanga M, et al. Effectiveness of short message services reminder on childhood immunization programme in Kadoma, Zimbabwe ‐ a randomized controlled trial, 2013. BMC Public Health 2015;15:137.

**Bigna 2015**

- Bigna JJ, Noubiap JJ, Kouanfack C, Plottel CS, Koulla‐Shiro S. Effect of mobile phone reminders on follow‐up medical care of children exposed to or infected with HIV in Cameroon (MORE CARE): a multicentre, single‐blind, factorial, randomised controlled trial. Lancet Infectious Diseases 2014;14(7):600‐8.
- Bigna JJ, Noubiap JJ, Plottel CS, Kouanfack C, Koulla‐Shiro S. Barriers to the implementation of mobile phone reminders in pediatric HIV care: a pre‐trial analysis of the Cameroonian MORE CARE study. BMC Health Services Research 2014;14:523.
- Bigna JJ, Noubiap JJ, Plottel CS, Kouanfack C, Koulla‐Shiro S. Factors associated with non‐adherence to scheduled medical follow‐up appointments among Cameroonian children requiring HIV care: a case‐control analysis of the usual‐care group in the MORE CARE trial. Infectious Diseases of Poverty 2014;3(1):44.
- Bigna JJ. Automated text message reminders to promote good health. Lancet Infectious Diseases 2015;15(1):19‐20.
- Mbuagbaw L. Mobile phone reminders for paediatric HIV follow‐up care. Lancet Infectious Diseases 2014;14(7):540‐1.

**Brown 2016**

- Brown VB, Oluwatosin OA, Akinyemi JO, Adeyemo AA. Effects of community health nurse‐led intervention on childhood routine immunization completion in primary health care centers in Ibadan, Nigeria. Journal of Community Health 2016;41(2):265‐73.
- Brown VB, Oluwatosin A, Ogundeji MO. Experiences, perceptions and preferences of mothers towards childhood immunization reminder/recall in Ibadan, Nigeria: a cross‐sectional study. Pan African Medical Journal 2015;20:243.
- Brown VB, Oluwatosin OA. Feasibility of implementing a cellphone‐based reminder/recall strategy to improve childhood routine immunization in a low‐resource setting: a descriptive report. BMC Health Services Research 2017;17(2):703.

**Domek 2016**

- Domek GJ, Contreras‐Roldan IL, O'Leary ST, Bull S, Furniss A, Kempe A, et al. SMS text message reminders to improve infant vaccination coverage in Guatemala: a pilot randomized controlled trial. Vaccine 2016;34(21):2437‐43.
- Domek GJ, Contreras‐Roldan IL, Asturias EJ, Bronsert M, Ventura GAB, O’Leary ST, et al. Characteristics of mobile phone access and usage in rural and urban Guatemala: assessing feasibility of text message reminders to increase childhood immunizations. mHealth 2018;4:9.

**Eze 2015**

- Eze GU, Adeleye OO. Enhancing routine immunization performance using innovative technology in an urban area of Nigeria. West African Journal of Medicine 2015;34(1):3‐10.

**Gibson 2017**

- Gibson DG, Ochieng B, Kagucia EW, Were J, Hayford K, Moulton LH, et al. Mobile phone‐delivered reminders and incentives to improve childhood immunisation coverage and timeliness in Kenya (M‐SIMU): a cluster randomised controlled trial. Lancet Global Health 2017;5(4):e428‐38.

**Haji 2016**

- Haji A, Lowther S, Ngan'ga Z, Gura Z, Tabu C, Sandhu H, et al. Reducing routine vaccination dropout rates: evaluating two interventions in three Kenyan districts, 2014. BMC Public Health 2016;16:152.

**Hannan 2016**

- Hannan J, Brooten D, Page T, Galindo A, Torres M. Low‐income first‐time mothers: effects of APN follow‐up using mobile technology on maternal and infant outcomes. Global Pediatric Health 2016;3:DOI: 10.1177/2333794X16660234.

**Hofstetter 2015**

- Hofstetter AM, DuRivage N, Vargas CY, Camargo S, Vawdrey DK, Fisher A, et al. Text message reminders for timely routine MMR vaccination: a randomized controlled trial. Vaccine 2015;33(43):5741‐6.

**Jimenez 2017**

- Jimenez ME, DuRivage NE, Bezpalko O, Suh A, Wade R, Blum NJ, et al. A pilot randomized trial of a video patient decision aid to facilitate early intervention referrals from primary care. Clinical Pediatrics 2017;56(3):268‐77.

**Niederhauser 2015**

- Niederhauser V, Johnson M, Tavakoli AS. Vaccines4Kids: assessing the impact of text message reminders on immunization rates in infants. Vaccine 2015;33(26):2984‐9.

**Sharma 2011**

- Sharma R, Hebbal M, Ankola AV, Murugabupathy V. Mobile‐phone text messaging (SMS) for providing oral health education to mothers of preschool children in Belgaum City. Journal of Telemedicine & Telecare 2011;17(8):432‐6.

**Stockwell 2015**

- Stockwell MS, Hofstetter AM, DuRivage N, Barrett A, Fernandez N, Vargas CY, et al. Text message reminders for second dose of influenza vaccine: a randomized controlled trial. Pediatrics 2015;135(1):e83‐91.

#### Mothers living with HIV/AIDS (N = 3)

**Kassaye 2016**

- Kassaye SG, Ong'ech J, Sirengo M, Kose J, Matu L, McOdida P, et al. Cluster‐randomized controlled study of SMS text messages for prevention of mother‐to‐child transmission of HIV in rural Kenya. AIDS Research & Treatment 2016;2016:1289328.

- Jennings L, Ong’ech J, Simiyu R, Sirengo M, Kassaye S. Exploring the use of mobile phone technology for the enhancement of the prevention of mother‐to‐child transmission of HIV program in Nyanza, Kenya: a qualitative study. BMC Public Health 2013;13(1):1131.

**Kebaya 2014**

- Kebaya L, Nduati R, Wamalwa D, Kariuki N, Bashir A. Efficacy of mobile phone use on adherence to nevirapine prophylaxis and retention in care among the HIV‐exposed infants in PMTCT: a randomised controlled trial. Archives of Disease in Childhood 2014;99:A329.
- Kebaya LM, Wamalwa D, Kariuki N, Admani B, Nduati RW. Efficacy of mobile phone use on adherence to nevirapine prophylaxis and retention in care among HIV‐exposed infants. Topics in Antiviral Medicine 2015;23:407.

**Odeny 2014**

- Odeny TA, Bukusi EA, Cohen CR, Yuhas K, Camlin CS, McClelland RS. Texting improves testing: a randomized trial of two‐way SMS to increase postpartum prevention of mother‐to‐child transmission retention and infant HIV testing. AIDS 2014;28(15):2307‐12.
- Odeny TA, Newman M, Bukusi EA, McClelland RS, Cohen CR, Camlin CS. Developing content for a mHealth intervention to promote postpartum retention in prevention of mother‐to‐child HIV transmission programs and early infant diagnosis of HIV: a qualitative study. PLOS ONE 2014;9(9):e106383.

## Characteristics of studies

### Characteristics of included studies [ordered by study ID]

**Table:** Akinfaderin‐Agarau 2012

Country	Nigeria	
Participant group	For adolescent and youth populations as potential users of sexual and reproductive health services For adult populations as potential users of sexual and reproductive health services	
Intervention channel	Mobile phones	
Health topic	Sexual and reproductive health in general	
Data collection methods	Focus group discussions	
Notes

**Table:** Brown 2014

Country	USA	
Participant group	For pregnant and postpartum women (up to 6 weeks) Parents and other caregivers of children under 5 years of age	
Intervention channel	SMS text blasts	
Health topic	Promotion of breastfeeding, effects of breast milk on infant growth and development, information about infant immunisations, and reminders about infant and maternal follow‐up and well‐being	
Data collection methods	Semi‐structured interviews	
Notes

**Table:** Calderón 2017

Country	Peru	
Participant group	Parents and other caregivers of children under 5 years of age	
Intervention channel	SMS	
Health topic	Child health	
Data collection methods	Interviews	
Notes

**Table:** Cates 2015

Country	USA	
Participant group	For adolescent and youth populations as potential users of sexual and reproductive health services	
Intervention channel	SMS	
Health topic	Human papillomavirus (HPV) vaccine	
Data collection methods	Focus group discussions and in class surveys	
Notes

**Table:** Cornelius 2009

Country	USA	
Participant group	For adolescent and youth populations as potential users of sexual and reproductive health services	
Intervention channel	SMS	
Health topic	HIV	
Data collection methods	Focus group discussions	
Notes

**Table:** Curioso 2009

Country	Peru	
Participant group	For adult populations as potential users of sexual and reproductive health services	
Intervention channel	SMS	
Health topic	HIV	
Data collection methods	Focus group discussions	
Notes

**Table:** Entsieh 2015

Country	Ghana	
Participant group	For pregnant and postpartum women (up to 6 weeks) Parents and other caregivers of children under 5 years of age	
Intervention channel	App “Mobile midwife”	
Health topic	Advice on maternal and newborn health	
Data collection methods	Focus group discussions and interviews	
Notes

**Table:** Evans 2016

Country	UK	
Participant group	For adolescent and youth populations as potential users of sexual and reproductive health services For adult populations as potential users of sexual and reproductive health services	
Intervention channel	SMS	
Health topic	HIV	
Data collection methods	Focus group discussions	
Notes

**Table:** Flax 2017

Country	Nigeria	
Participant group	Unclear. Pregnant and breastfeeding women as well as women without children	
Intervention channel	SMS	
Health topic	Breastfeeding	
Data collection methods	Telephone exit interviews, in‐depth interviews, and focus group discussions	
Notes

**Table:** French 2016

Country	UK	
Participant group	For adolescent and youth populations as potential users of sexual and reproductive health services	
Intervention channel	SMS	
Health topic	Sexually transmitted infections	
Data collection methods	Semi‐structured telephone interviews	
Notes

**Table:** Gold 2010

Country	Australia	
Participant group	For adolescent and youth populations as potential users of sexual and reproductive health services	
Intervention channel	SMS	
Health topic	Sexually transmitted infections	
Data collection methods	Focus group discussions	
Notes

**Table:** Goldenberg 2015

Country	USA	
Participant group	For adolescent and youth populations as potential users of sexual and reproductive health services For adult populations as potential users of sexual and reproductive health services	
Intervention channel	SMS	
Health topic	HIV testing reminders	
Data collection methods	Focus group discussions	
Notes

**Table:** Greaney 2014

Country	USA	
Participant group	For adolescent and youth populations as potential users of sexual and reproductive health services For adult populations as potential users of sexual and reproductive health services	
Intervention channel	Interactive voice response messages	
Health topic	Cancer screening	
Data collection methods	Focus group discussions	
Notes

**Table:** Hirsch‐Moverman 2017

Country	Lesotho	
Participant group	For adult populations as potential users of sexual and reproductive health services	
Intervention channel	SMS	
Health topic	HIV	
Data collection methods	In‐depth interviews	
Notes

**Table:** Jalloh‐Vos 2014

Country	Sierra Leone	
Participant group	Pregnant and postpartum women and adults for family planning	
Intervention channel	SMS and voice call	
Health topic	Antenatal care and family planning	
Data collection methods	Semi‐structured interviews	
Notes

**Table:** Jennings 2013

Country	Kenya	
Participant group	For pregnant and postpartum women (up to 6 weeks)	
Intervention channel	SMS	
Health topic	Prevention of mother‐to‐child transmission (PMTCT)	
Data collection methods	Focus group discussions and in‐depth interviews	
Notes

**Table:** Lau 2014

Country	South Africa	
Participant group	For pregnant and postpartum women (up to 6 weeks)	
Intervention channel	SMS	
Health topic	Antenatal health promotion	
Data collection methods	Focus group discussions	
Notes

**Table:** Mbuagbaw 2012

Country	Cameroon	
Participant group	For adult populations as potential users of sexual and reproductive health services	
Intervention channel	SMS	
Health topic	HIV drug adherence	
Data collection methods	Group interviews	
Notes

**Table:** Mbuagbaw 2014

Country	Cameroon	
Participant group	For adult populations as potential users of sexual and reproductive health services	
Intervention channel	SMS	
Health topic	People living with HIV/AIDS	
Data collection methods	Focus group discussions	
Notes

**Table:** Menacho 2013

Country	Peru	
Participant group	For adolescent and youth populations as potential users of sexual and reproductive health services For adult populations as potential users of sexual and reproductive health services	
Intervention channel	SMS	
Health topic	Motivate men who have sex with men to go for HIV testing	
Data collection methods	Focus group discussions	
Notes

**Table:** Missal 2016

Country	India	
Participant group	For pregnant and postpartum women (up to 6 weeks)	
Intervention channel	SMS	
Health topic	Antenatal health promotion	
Data collection methods	Focus group discussions	
Notes

**Table:** Mitchell 2016

Country	USA	
Participant group	For adolescent and youth populations as potential users of sexual and reproductive health services For adult populations as potential users of sexual and reproductive health services	
Intervention channel	An app	
Health topic	To motivate men who have sex with men to go for HIV testing	
Data collection methods	Focus group discussions	
Notes

**Table:** Munro 2017

Country	Canada	
Participant group	For pregnant and postpartum women (up to 6 weeks) Parents and other caregivers of children under 5 years of age	
Intervention channel	SMS “Text4baby program”	
Health topic	Prenatal education	
Data collection methods	Demographic questionnaire and focus group discussions	
Notes

**Table:** Nachega 2016

Country	South Africa	
Participant group	For pregnant and postpartum women (up to 6 weeks) who were HIV‐positive	
Intervention channel	SMS	
Health topic	Antiretroviral therapy adherence to prevent mother‐to‐child transmission of HIV	
Data collection methods	Focus group discussions	
Notes

**Table:** Naughton 2013

Country	UK	
Participant group	For pregnant and postpartum women (up to 6 weeks)	
Intervention channel	SMS	
Health topic	Smoking cessation during pregnancy	
Data collection methods	Focus group discussions and semi‐structured interviews	
Notes

**Table:** Odeny 2014

Country	Kenya	
Participant group	For pregnant and postpartum women (up to 6 weeks)	
Intervention channel	SMS	
Health topic	Early infant HIV testing	
Data collection methods	Focus group discussions	
Notes

**Table:** Perry 2012

Country	USA	
Participant group	For adolescent and youth populations as potential users of sexual and reproductive health services	
Intervention channel	SMS	
Health topic	Preventative sexual health messaging	
Data collection methods	Focus group discussions	
Notes

**Table:** Rana 2015

Country	Uganda	
Participant group	For adolescent and youth populations as potential users of sexual and reproductive health services	
Intervention channel	SMS	
Health topic	HIV antiretroviral therapy adherence	
Data collection methods	Focus group discussions	
Notes

**Table:** Rodrigues 2015

Country	India	
Participant group	For adult populations as potential users of sexual and reproductive health services	
Intervention channel	Interactive voice recordings and SMS	
Health topic	HIV antiretroviral therapy adherence	
Data collection methods	Interviews	
Notes

**Table:** Sloan 2017

Country	UK	
Participant group	For pregnant and postpartum women (up to 6 weeks)	
Intervention channel	SMS	
Health topic	Smoking cessation during pregnancy	
Data collection methods	Semi‐structured interviews	
Notes

**Table:** Smillie 2014

Country	Canada	
Participant group	For adolescent and youth populations as potential users of sexual and reproductive health services For adult populations as potential users of sexual and reproductive health services	
Intervention channel	SMS	
Health topic	HIV	
Data collection methods	Semi‐structured interviews	
Notes

**Table:** Smith 2017

Country	Cambodia	
Participant group	For adult populations as potential users of sexual and reproductive health services	
Intervention channel	Mobile phone voice messaging and counsellor support	
Health topic	Abortion care	
Data collection methods	Semi‐structured interviews	
Notes

**Table:** Ware 2016

Country	Uganda	
Participant group	For adult populations as potential users of sexual and reproductive health services	
Intervention channel	SMS combined with real‐time adherence monitoring	
Health topic	HIV	
Data collection methods	Interviews	
Notes

**Table:** Willoughby 2017

Country	USA	
Participant group	For adult populations as potential users of sexual and reproductive health services	
Intervention channel	SMS	
Health topic	Sexual health promotion	
Data collection methods	Focus group discussions	
Notes

**Table:** Wright 2011

Country	USA	
Participant group	For adult populations as potential users of sexual and reproductive health services	
Intervention channel	SMS	
Health topic	Sexual health promotion	
Data collection methods	Focus group discussions	
Notes		

### Characteristics of excluded studies [ordered by study ID]

**Table:**

Study	Reason for exclusion
Abbass‐Dick 2017	Wrong study design
Abensur 2011	Wrong study design
Abramson 2015	Wrong delivery mechanism
Acevedo 1998	Wrong delivery mechanism
Adanikin 2014	Wrong study design
Agarwal 2014	Wrong study design
Ahlers‐Schmidt 2012	Wrong study design
Alexander 2014	Wrong study population
Aradhya 2013	Wrong study design
Asiodu 2015	Wrong delivery mechanism
Associates 2015	Wrong study design
Associates 2015a	Wrong study design
Azih 2012	Wrong study design
Bacchus 2016	Wrong delivery mechanism
Barnett 2016	Wrong topic of interest
Belzer 2015	Wrong study design
Beratarrechea 2015	Language not spoken by review team members
Biediger‐Friedman 2016	Wrong topic of interest
Birukila 2017	Wrong study design
Blas 2013	Wrong topic of interest
Brayboy 2017	Wrong study design
Brinkel 2017	Wrong intervention (telemedicine)
Broom 2015	Wrong study design
Brüll 2016	Wrong delivery mechanism
Bull 2010	Wrong delivery mechanism
Campbell 2015	Wrong study design
Chang 2013	Wrong topic of interest
Chib 2013	Wrong study design
Cordova 2015	Wrong topic of interest
Cormick 2012	Wrong study design
Cormick 2015	Wrong study design
Curioso 2007	Wrong topic of interest
Dean 2012	Wrong intervention
Devine 2014	Wrong topic of interest
Fletcher 2016	Wrong topic of interest
Fornos 2014	Wrong topic of interest
Gatwood 2014	Wrong study population
Goldenberg 2014	Wrong delivery mechanism
Hearn 2014	Wrong delivery mechanism
Herbec 2014	Wrong delivery mechanism
Hmone 2016	Wrong topic of interest
Holloway 2017	Wrong delivery mechanism
Horvath 2016	Wrong delivery mechanism
Huberty 2015	Wrong topic of interest
Huq 2014	Wrong delivery mechanism
Irons 2015	Wrong study design
Jamison 2013	Wrong intervention
Kharbanda 2009	Wrong study population
Kharbanda 2011	Wrong study population
Labacher 2013	Wrong study design
Lee 2014	Wrong study design
Levine 2008	Wrong study design
Lewis 2013	Wrong study design
Maar 2016	Wrong study population
Marsh 2014	Wrong delivery mechanism
Mbuagbaw 2013	Wrong study design
Michell 2014	Wrong study design
Moskowitz 2009	Wrong delivery mechanism
Muessig 2013	Wrong delivery mechanism
O'donnell 2016	Wrong study design
Price 2009	Wrong study design
Prieto 2016	Wrong intervention
Puccio 2006	Wrong study design
Pérez 2015	Wrong study design
Ramanathan 2013	Wrong intervention
Ranney 2014	Wrong topic of interest
Redfern 2016	Wrong patient population
Saranto 2009	Wrong study design
Schnall 2014	Wrong delivery mechanism
Schnall 2016	Wrong delivery mechanism
Senn 2017	Wrong intervention
Senn 2017a	Wrong study design
Shet 2010	Wrong study design
Sidney 2012	Wrong study design
Siedner 2012	Wrong study design
Skeels 2006	Wrong topic of interest
Smith 2015	Wrong study design
Soltani 2012	Wrong topic of interest
Soltani 2015	Wrong study design
Suwamaru 2012	Wrong study design
Thomas 2017	Wrong topic of interest
Thompson 2016	Wrong study design
Toefy 2016	Wrong study design
Tran 2012	Wrong study design
Uhrig 2012	Wrong study design
Vahdat 2013	Wrong study design
Vyas 2012	Wrong patient population
WHO 2014	Wrong study design
Wilkinson 2017	Wrong study design
Ybarra 2016	Wrong study design
